# Metabolic Rewiring in Radiation Oncology Toward Improving the Therapeutic Ratio

**DOI:** 10.3389/fonc.2021.653621

**Published:** 2021-05-10

**Authors:** Marike W. van Gisbergen, Emma Zwilling, Ludwig J. Dubois

**Affiliations:** ^1^ The M-Lab, Department of Precision Medicine, GROW-School for Oncology and Developmental Biology, Maastricht University, Maastricht, Netherlands; ^2^ Department of Dermatology, GROW-School for Oncology and Developmental Biology, Maastricht University Medical Center+, Maastricht, Netherlands

**Keywords:** radiation, radiotherapy, metabolism, cancer, oncology, drug repurposing

## Abstract

To meet the anabolic demands of the proliferative potential of tumor cells, malignant cells tend to rewire their metabolic pathways. Although different types of malignant cells share this phenomenon, there is a large intracellular variability how these metabolic patterns are altered. Fortunately, differences in metabolic patterns between normal tissue and malignant cells can be exploited to increase the therapeutic ratio. Modulation of cellular metabolism to improve treatment outcome is an emerging field proposing a variety of promising strategies in primary tumor and metastatic lesion treatment. These strategies, capable of either sensitizing or protecting tissues, target either tumor or normal tissue and are often focused on modulating of tissue oxygenation, hypoxia-inducible factor (HIF) stabilization, glucose metabolism, mitochondrial function and the redox balance. Several compounds or therapies are still in under (pre-)clinical development, while others are already used in clinical practice. Here, we describe different strategies from bench to bedside to optimize the therapeutic ratio through modulation of the cellular metabolism. This review gives an overview of the current state on development and the mechanism of action of modulators affecting cellular metabolism with the aim to improve the radiotherapy response on tumors or to protect the normal tissue and therefore contribute to an improved therapeutic ratio.

## Introduction

Cancer incidence is predicted to almost double, from 12.7 million in 2008 to 22.2 million cases in 2030 (1), resulting in a high burden on the healthcare system, but also necessitating the demand for efficient therapies. Although cancer survival rates are improved over the last decades, metastases contribute for the majority of cancer-related deaths (2). Approximately 50% of all cancer patients undergo radiotherapy during their treatment, either as monotherapy, but more frequently in combination with surgery, chemotherapy or immunotherapy, either to treat primary tumors and/or metastasis, or as a palliative treatment (3–11). Different therapies are commonly combined as it improves tumor control, progression-free survival and overall survival rates (12–16). Unfortunately, combination treatments approaches can potentially increase systemic toxicity. In order to overcome this problem, there is a need for a higher therapeutic selectively, which can be accomplished by enhancing tumor treatment sensitivity while reducing adverse effects. This improved therapeutic ratio, is a favorable tradeoff between the tumor control and the radiation-induced toxicity (17).

One of the hallmarks of many cancer cells, namely the reprogramming of the cellular metabolism, has recently gained again the attention of researchers and clinicians. Rewiring the malignant metabolism is an interesting approach to considerably affect the radiotherapy response in order to enhance the therapeutic ratio (18, 19). This review gives an overview on the mechanisms and current status of potential radiosensitizers and protectors in relationship to rewire the cellular metabolism and thereby enhancing the therapeutic ratio (**Tables 1** and **2**).

**Table 1:** Compounds with potentially radiosensitizing properties.

Compound	Mode of action	Specificities	References
**HIF-1 signaling**			
Deguelin, SH-14	Akt inhibition, downregulation HIF-1α, reduced hexokinase expression	Potential CI inhibitor- Development of Parkinson’s disease-like syndrome in rat; SH-14 is a deguelin derivative	(20, 21)
Vandetanib	Inhibition, EGFR, HIF-1α signaling interference	FDA-approved for medullary thyroid cancer therapy	(22–27)
Berberine	Downregulation HIF-1α & VEGF		(28–31)
Rg3	Inhibition NF-κB, decreased expression HIF-1α & VEGF		(32, 33)
**Cellular metabolism**			
*Glucose metabolism*			
BAY-876	GLUT1 inhibition	*In vitro* cisplatin sensitizer, radiosensitizing effects unclear	(34, 35)
WZB117	GLUT1 inhibition		(36, 37)
2-DG, WP1122	Glucose analogue, hexokinase inhibition, radiosensitizing mechanism unclear	Tumor staging and metabolism profiling *via* PET-imaging using 2-DG coupled to positron-emitting isotopes; WP1122 is a 2-DG analogue	(38–41)
Lonidamine	Glycolysis inhibition, TCA cycle & CII interference	Negative results in clinics	(42–44)
Devimistat	Deregulation TCA cycle enzymes, ROS induction	In phase 2/3 trials combined with chemotherapeutics; No studies about combination with radiotherapy	(45, 46)
FH535 and Y3	Distortion mitochondrial membrane potential, apoptosis inducer	Y3 is an FH535-analogue	(47, 48)
Ivosidenib	IDH1_mut_ inhibition	FDA-approved for acute/refractory AML	(49)
Enasidenib	IDH2_mut_ inhibition	FDA-approved for acute/refractory AML	(50, 51)
Vorasidenib	IDH1/2_mut_ inhibition	Clinical trials	(52, 53)
BAY-1436032	IDH1_mut_ inhibition	Clinical trials	(54, 55)
*Complex I*			
Metformin	Inhibition CI, oxygen accumulation and subsequent HIF-1-α destabilization, reduces PI3K/Akt signaling	FDA-approved for anti-diabetes therapy	(56, 57)
Phenformin	CI inhibition	Redrawn, induces lactic acidosis in diabetes patients; Clinical trials phase I	(58, 59)
Papaverine	CI inhibition and PDE10A	FDA-approved as anti-vasospasm therapeutic*; In vivo* radiosensitization	(60, 61)
SMV-32	CI inhibition	Papaverine derivative*; In vivo* radiosensitizing; Clinical trials phase I	(61)
BAY 87 2243		*In vivo* radiosensitizing; Clinical trials status unclear	NCT01297530
IACS-010759		Radiosensitizing effect unclear	NCT03291938 NCT02882321
*Complex III*			
Atovaquone	Complex III inhibition	FDA-approved for anti-malaria therapy; Clinical trials phase I	(62, 63)
Pyrazinib	OCR/ECAR reduction	Precise target unknown	(64)
*Other pathways*			
ADI-PEG	Arginine depletion	Arginine deiminase and polyethylene glycol chimera- Clinical trials in combination with chemotherapy	(65–67)
Orlistat	FASN inhibition	FDA-approved for obesity-management	(68)
Fenofibrate	Activates PPARα, metabolic reprogramming *via* CPT1, AMPK and HK2 Prevention of HIF-1 stabilization	FDA-approved for hypercholesterolemia, mixed dyslipidemia and severe hypertriglyceridemia	(69–71)
**Redox signaling**			
Telaglenastat	GLS inhibition	Improved bioavailability, chemotherapeutic and immunotherapeutic outcomesFDA-approved for advanced renal cell carcinomas	(72–75)
Auranofin	TrX-reductase inhibition	FDA-approved for arthritis therapy	(41, 76)

The compounds are grouped according to their intracellular effects.

**Table 2:** Compounds with potentially radioprotective properties.

Compound	Mode of action	Specificities	References
**Radical scavengers**			
Amifostine, PrC-210	ROS scavenger, accumulates in normal tissue, precise mechanism unknown	FDA approved for radioprotective effectsPrC-210 is an amifostine analogue	(77–81)
MNSOD-PL	Mitochondrial localization		(82)
JP4-039	fusion peptide, Mitochondrial localization		(83, 84)
DSePA	ROS scavenger	Limited tumor uptake accumulation in lung and intestine	(85, 86)
**Inflammation mitigators**			
Celecoxib	COX2 inhibitor	Reduces skin toxicity, phase 2 clinical trialFDA-approved for rheumatoid arthritis	(87)
Vitamin E, γ-tocotrienol	Reduction in radiation-induced lipid peroxidation	Tumor radiosensitizing effectsγ-tocotrienol is a Vitamin E derivative	(88, 89)
Ascorbic acid	Downregulation of MnSOD in tumors		(90, 91)
Curcumin	Blocks NF-κB signaling		(92)
Melatonin	NF-κB downregulation, depleting hydroxyl radicals,stimulation of SOD and GPx		(93–97)
CAPE	Suppression of NF-κB-signaling, ROS disbalance	Tumor radiosensitizing effects	(98, 99)
**DNA repair**			
RSV, HS-1793	Mcl-1 downregulation, cell cycle arrest, DNA repair	HS-1793 is an RSV analogue	(100–102)
**Dietary Intervention**			
STF	Differential stress response,	Phase I/II clinical trial	(103–105) NCT01754350
Ketogenic Diet	Differential stress response	Phase I/II clinical trial- Used in epilepsy treatmentClinical trials for cancer treatment ongoing	(106) NCT03451799 NCT02516501

The compounds are grouped according to their intracellular effects.

## Metabolic Rewiring in Cancer

Metabolic plasticity is crucial to ensure cellular survival since it enables the cell to adapt to changing nutrient demands and oxygen conditions. Therefore, metabolic adjustments are tightly regulated by extrinsic signals, such as growth factors, mediating the cell’s response to changed environmental conditions. In contrast to healthy cells, the metabolism of primary cancer cells or metastatic lesions is, to a certain extent, uncoupled from external stimuli providing continuous resources for proliferation, growth, and metastatic niche formation (107–109). This is caused for instance by oncogene activation and/or loss of function in tumor suppressors. Consequently, malignant cells are depended on anabolic pathways and rewire their metabolism to meet their increasing demand for adenosine triphosphate (ATP), macromolecules and reactive oxygen species (ROS) scavengers due to their high proliferative potential (110). Metabolic patterns vary between cancer cells, depending on several intrinsic and extrinsic factors, such as the oncogene type and its microenvironment. The tumor microenvironment is often, in contrast to normal tissues, deprived from nutrients and oxygen due to a poor and imbalanced vascularization. Tumor cells therefore are capable to adapt their metabolic need by restructuring their metabolism and maintain a high biosynthetic potential by altering their carbon metabolism such as their intracellular glucose or glutamine pathways (111–113).

A prominent example for the rewiring of metabolic pathways in cancer cells is the “aerobe glycolysis,” also named the Warburg effect. Aerobe glycolytic cells display an increased glucose uptake which they convert, in contrast to normal cells, into lactate instead of pyruvate in presence of oxygen (114–116). This seems quite paradoxal since oxidative phosphorylation (OXPHOS) remains possible as oxygen is available and has a higher ATP yield. However, tumor proliferation also depends on anabolic pathways derived from the glycolysis. A balanced exit of glycolytic intermediates ensures that the anabolic pathways are constantly replenished and (intermediate) products are transferred to the pentose‑phosphate-pathway (PPP), fatty acid (FA) synthesis and tricarboxylic acid (TCA) cycle in order to meet tumor’s metabolic need to proliferate. Glucose-6-phosphate is a central glycolytic intermediate as it can either be used by an irreversible oxidative arm and a reversible non-oxidative arm of which the oxidative arm contributes to reduced nicotinamide adenine dinucleotide phosphate (NADPH) production. The metabolic flux of the PPP is important to maintain a redox balance as reduced NADPH is a necessary cofactor for FA-synthesis and glutathione peroxidase (117, 118). The sufficient supply of anabolic pathways with intermediates is ensured by an altered regulation, for example the overexpression of the pyruvate kinase M2 (PKM2) in many tumors. This less efficient splice variant of the pyruvate kinase M1 catalyzes the conversion of phosphoenolpyruvate to pyruvate limiting the influx of pyruvate into the TCA cycle (119–121). Despite the higher fermentation rate, many cancers with an intact mitochondrial function still maintain their ATP pool using the Electron Transport Chain (ETC) and ATP-synthase (122). Due to the limited pyruvate production, alternative anaplerotic pathways are very important to sustain the TCA cycle, which recycles reduction equivalents that are crucial for the redox balance.

Next to glucose, also glutamine can refill the TCA cycle and maintain redox homeostasis. The glutamine pathway fuels the TCA cycle *via* glutamate and α‑KG where oxaloacetate (OAA) gets converted into aspartate in order to support nucleotide synthesis (113). Vazquez et al. has given a comprehensive overview and graphical representation of the different substrates and connections between different oncological metabolic pathways (123). Extracellular glutamine is transported into the cells using transporters like SLC1A5. However, under nutritional stress conditions, tumor cells are also able to acquire glutamine by macromolecule breakdown within the cell (113). The uptake of those macromolecules by macropinocytosis can be stimulated by the oncogene *RAS* (113, 124). A large variety of oncogenes and tumor suppressors (e.g. *MYC, KRAS*, HIF-1α stabilization, *mTOR, P53, PTEN* etc.) have been found to influence the glutamine metabolism and its effector pathways, emphasizing the importance of the glutamine pathway in tumor cell development, expansion and metastatic properties (113).

Cancers also often upregulate the *de novo* FA synthesis to provide enough lipids for membranes and other cellular structures (125). ATP-citrate lyase (ACT) is often upregulated to convert the TCA cycle derived citrate into acetyl-CoA. Consequently, a higher substrate influx into the FA synthesis occurs (126, 127). Lipid droplets are closely related to the fatty acids as they serve as storage depots of fatty acids. Lipid droplets can influence the metabolic regulation of tumor cells and tumor-associated immune cells. A relationship between lipid droplet accumulation, tumor establishment and aggressiveness has been demonstrated in different types of cancer, although this seems tissue type specific. In hypoxic tumor regions specifically, accumulation of lipid droplets has been observed, which is potentially related to an increased Fatty Acid Oxidation (FAO). The role of lipid droplets has been extensively reviewed before (128, 129). Inhibition of lipid droplet formation could potentially serve as a novel therapeutic target to be used in combination with therapies, such as radiotherapy, which modulate metabolism.

All these metabolic alterations are merely examples of metabolic rewiring to facilitate fast proliferation, growth and spread of cancer cells [extensively reviewed by (117, 130, 131)]. Importantly, the metabolic pattern varies between cancer cells and their tumor microenvironmental conditions. Interestingly, it has been suggested that cancer therapy itself, such as radiotherapy, can influence the cellular metabolism, which eventually will affect the cellular response to radiation (132–134). Therefore, a more profound understanding of these interactions is needed in order to enhance the therapeutic ratio.

## Radiotherapy and Metabolism

Radiotherapy is a fundamental treatment for most cancer patients since it enables the local control of many cancer types (3). Radiation results in deoxyribonucleic acid (DNA) double strand breaks (DSBs), single strand breaks (SSBs) and the radiolysis of water and other intracellular molecules, resulting in a ROS burst (135). This causes lipid peroxidation, protein oxidation and DNA damage, all processes massively harming cellular viability (136–141). Some lesions will remain unrepaired, resulting in genomic instability and cell death by mitotic catastrophe, even several mitotic divisions post radiotherapy. Cancer cells are more vulnerable to irradiation than normal cells since their DNA repair machinery is less efficient, making them more prone to genomic instability (142, 143).

Genotoxic effects of radiation are presumed to be caused by direct irradiation of the nucleus and not to the cytoplasm of cells, as direct irradiation of the nucleus is more lethal than the cytoplasmic dose (144). DNA repair is a highly energy demanding process in both tumor and normal tissue cells as interactions have been observed between mitochondrial ATP generation, DNA-repair and cell cycle kinase CDK1 (145, 146). Also, chromatin remodeling is an important mechanism involving DNA repair. Chromatin relaxation is highly ATP dependent and inhibition of glucose uptake can lead to energetic stress that will result to a decreased tumor survival upon radiation (147). DNA folding and remodeling involves acetylation of the DNA and donors for this acetyl-group are derived from acetyl coenzyme A (CoA), which is also required for the TCA cycle. Acetate-derived acetyl-CoA has also been linked to histone acetylation, suggesting that acetyl-CoA is an important substrate for gene regulation (148, 149). Limiting metabolic substrates will therefore have severe implications on the ability of cells to repair their radiation-induced DNA damage (150).

Nonetheless, the success of radiotherapy considerably depends on the therapeutic ratio since the radiation dose given to the tumor is limited by the maximal dose tolerated by the surrounding normal tissue. Both phenomena therefore contribute to the therapeutic ratio and are possible targets to increase radiotherapy efficacy. Radiosensitivity varies between cells, tissues and individuals and is determined by several intrinsic and extrinsic factors. Generally, hypoxia and cellular metabolism are two crucial determinants of cellular radiotherapy-response (151, 152). Hypoxic areas of the tumor can emerge from the immature tumor vascularization and the OXPHOS-dependent increased oxygen consumption rate (OCR), both due to the enhanced proliferative potential of cancer cells. Hypoxic areas reduce the cellular radiosensitivity since cells in hypoxic environments lack oxygen, the main component for ROS formation and inducing radiation-induced genotoxicity (153–155). Tumor survival and re-growth upon radiotherapy is also relying on the formation of new blood vessels. However, the mechanisms behind this new vessel formation are still a matter of debate and can be broadly categorized in: 1) the requirement of bone marrow–derived cells, or 2) the remaining viable endothelial cells form these new vessels  (156–158).

Hypoxia regulates HIF signaling by promoting stabilization of HIF-1α, which can regulate target gene expression by binding to specific regulatory sequences in their promotor, the hypoxia response elements (HRE) (159). Hypoxia contributes to epithelial-mesenchymal transition (EMT) by binding to the HRE in TWIST (160, 161) and ZEB1 (162, 163), thereby supporting tumor invasiveness (164). Also, the downstream mechanisms of HIF-1α signaling influence cellular radioresistance by facilitating a metabolic switch, i.e. stimulation of glycolysis and OXPHOS downregulation, supporting the depletion of ROS and promoting angiogenesis (165–171). Importantly, HIF-1 expression is not only regulated by hypoxia, also the genetic background of tumors influences intracellular HIF-1 levels.

Tumor metabolism may also affect the radiation response since evidence suggests an enhanced radioresistance in cells harboring the Warburg phenotype. Studies observed an upregulation of glycolytic enzymes in Warburg-dependent cells, associated with elevated HIF-1 levels (172). Therefore, it is hypothesized that activated HIF-1 promotes the Warburg effect by e.g. activating glycolytic enzymes (173, 174). Genetic interference with HIF-1–mediated effects on the glycolysis results in radiosensitivity (175, 176).

### Metabolic Rewiring Upon Radiation of Tumors

Although malignant oncogene activation or loss of function of tumor suppressors alters the metabolism, radiation itself may also enhance metabolic alterations by influencing different signaling pathways. Among the several affected pathways, the PI3K/Akt and the NF‑κB pathway play a crucial role in radiation-induced metabolic remodeling and the tissue response to radiotherapy (133, 134). PI3K/Akt are master regulators of glucose uptake. Normally, the PI3K/Akt pathway is activated by external stimuli, however, in many cancers PI3K and its downstream target Akt are constitutively activated due to mutations (177). PI3K can also be indirectly activated by radiation through stimulation of the PI3K upstream epidermal growth factor receptors (EGFR) (132, 178–180). Akt overactivation facilitates glucose uptake and intracellular accumulation of glucose by enhancing the glucose transporter expression and by activating hexokinase and phosphofructokinase 1, respectively. Furthermore, Akt stimulates FA synthesis by activating ATP-citrate lyase (133). These alterations may nurture malignant cells and thus, many radiosensitizers have been used to influence this pathway (181).

NF-κB is a family of five master transcription factors, influencing the expression of various genes, which is deregulated in many cancers with various effects depending on the cellular context. Radiation stimulates the pathway by enhancing the DNA binding affinity of NF-κB, its expression, the dissociation of the IkB complex and consequently its activation (182, 183). Thalidomide is a for multiple myeloma U.S. Food and Drug Administration (FDA) approved NF-κB modulator that interferes with the NF-κB activation and is currently investigated to reduce urinary complications, a normal tissue complication, upon irradiation of the pelvic region (184). As radiation activates NF-κB- and PI3K/Akt-signaling and thereby affects the radiation response modulation of those signaling pathways, thalidomide can contribute to enhance the therapeutic window (185, 186).

Next to radiation, also manganese superoxide dismutase (MnSOD or SOD2) is able to activate NF-κB and contributes as such to an aggressive tumor phenotype (187). MnSOD is a well-known and an important anti-oxidant enzyme located in the mitochondria and is required to scavenge super-oxides generated by the OXPHOS. Due to its function, MnSOD also acts as a tumor suppressor and reduced MnSOD has been shown to contribute to the oncogenic transformation of cells (188). However, elevated MnSOD activity has been reported to be involved in the increased invasion and metastatic potential of tumors (189). MnSOD also seems to play a role in rewiring the tumor’s metabolism upon genotoxic conditions such as radiation exposure. CDK1 is found to contribute to mitochondrial energy production, which contributes to radiation induced DNA damage repair (145). MnSOD is able to interact with and activate these CDKs, and activated CDKs are involved in OXPHOS enhancement [reviewed by (190)]. This suggests that tumors are able to use the mitochondrial metabolism as a substitute metabolic system for glycolysis, when they are in a high metabolic need. Therefore, this phenomenon can contribute to growth, metastatic formation and a radiation resistance phenotype (190–192). Besides the signaling-pathway-mediated and radiation-induced metabolic shift of the tumor cells, also the metabolic rewiring of the TME and CSC population can play an important role in treatment response.

### Metabolic Rewiring Upon Radiation of the Tumor Micro-Environment

As radio-, and/or chemotherapy exert untargeted effects, induced metabolic alterations are not exclusively restricted to the irradiated tumor cells, but also include tissues that are in close proximity to the irradiated tumor tissue, i.e. the tumor micro-environment (TME). This comprises different cell and tissue types, secreted factors, and proteins resulting in a complex ecosystem which is shaped to promote tumor survival and to establish a connection with the whole organism contributing to cancer stemness and metastasis. Here, the malignant metabolism plays a pivotal role as the increased oxygen and nutrient consumption, as well as the release of several (onco-)metabolites and other factors such as growth factors, cytokines and extracellular vesicles, into the TME establishes interactions with neighboring cells, in order to promote tumor growth and therapy resistance (193–195).

#### Cancer-Associated Fibroblasts

One of the components of the TME is the stroma. It is composed of different cell types such as fibroblasts, mesenchymal stem cells, endothelial cells, and lymphocytes. Cancer-associated fibroblasts (CAFs) are a prominent example how cancer cells metabolically modulate the TME to promote tumorigenesis and metastasis. In contrast to normal fibroblasts, CAFs are continuously active, secreting growth factors and cytokines to promote tumor growth. Moreover, the elevated ROS levels in cancer cells mediate metabolic reprogramming of CAFs towards glycolysis followed by an increased MCT4-mediated export of lactate in the TME. The enhanced lactate uptake of aerobic cancer cells by upregulated expression of the MCT1-lactate transporter fuels malignant metabolism (196). This tumor feeding through the TME is involved in tumor metastasis and therapy resistance as it decreases the tumor’s dependency on proper vascularization, which is emphasized by the correlation of the tumor’s CAF infiltration and prognosis (197–201). Radiation has been shown to activate CAFs and enhance their proliferative potential. Co-culture experiments with cancer cells have suggested that CAFs have a radioprotective effect on cancer cells through integrin-signaling, which stimulates the invasive potential of cancer cells (200, 202, 203).

#### Immune Cells and (Onco-)Metabolites

Furthermore, tumors can influence, due to their altered metabolism, the TME to establish an immune suppressive environment. This is important as the TME comprises a variety of immune cells, including tumor-suppressing cells like natural killer (NK) cells, CD4/8^+^ T-cells, proinflammatory M1 macrophages, dendritic cells and tumor-promoting immune cells *e.g.*, Foxp3^+^ regulatory T-cells (Tregs), tumor-associated macrophages and myeloid-derived suppressor cells (MDSCs). The high anabolic rate of tumors results in hypoxic and nutrient-poor areas, which influence immune cell types massively as their function is determined by their metabolic program (204, 205). Glucose-deprivation results in T-cell and macrophage exhaustion as these cells depend on glycolysis to cover their demand for metabolic intermediates and energy (206). Moreover, the TME is a selective pressure for tumor-promoting immune cell types. Naïve T-cells favor the differentiation into Treg cells rather than into T-helper cells in a glucose and glutamine deficient TME as Tregs mainly rely on OXPHOS and FAO to meet their energy demand (207–209). The enhanced production of kynurenine caused by the expression of the tryptophan catabolizing IDO1 enzyme in cancer cells, cancer-derived vesicles and several other immune and stroma cells in the TME has immunosuppressing effects and is a predictor for poor prognosis in different cancer types. IDO1 stimulates the Treg-dependent recruitment and activation of MDSCs and the differentiation of CD4^+^ to Tregs (210–214). Concomitantly, IDO1 inhibits tumor-antagonists like CD8^+^ T-cells and NK cells (215, 216).

Radiation of the TME however provokes ambiguous responses, which is likely due to the heterogeneity in TME composition and the radiotherapy dosage (217). For example, radiation can exert immunosuppressive effects by decreasing the relative abundance of immunoreactive lymphocytes due to their inert higher radiosensitivity compared to immunosuppressive cells like MDSCs and Tregs (218–221). MDSCs exert immunosuppressive functions, amongst others, by depleting amino acids such as cysteine and arginine from the TME which impairs the function and activity of T-cells (222–224). Studies with high-dose or hypofractionated radiotherapy suggest that radiation potentially triggers an anti-tumor immune response. The release of cytokines, damage associated molecular patterns, tumor associated antigens and other factors by dying cells upon radiation activates CD8^+^ T-cells, although this seems to be dependent on radiation-induced conventional DC1 activation which mediates the cross-priming of CD8^+^ T-cells in the lymph nodes (225–228). The treatment with adjuvants rescued the absence of an improved radiation response in tumors with no DC1 activation. These observed differences may be due to the TME as for instance cancer cells are proposed to reduce the activity and functionality of DCs by inducing a Msr-mediated lipid uptake, which results in lipid accumulation and decreased antigen presentation (229, 230).

Another mechanism of tumors to interact with neighboring cells is the secretion of (onco-)metabolites from the TME, which are associated with tumor-promoting effects (231). The release of lactate, the end product of glycolysis, is promoted through hypoxia-mediated HIF-1 stabilization which results in the enhanced expression of monocarboxylate transporter 4 (MCT4). This leads to an increased acidification of the TME. Lactate exerts tumor-protecting effects by its inhibitory effects on T-cells, dendritic cells, natural killer cells, and tumor-associated macrophages (232–235) and its contribution to the induction of CD4^+^ Treg cells (234). Tumors with loss or gain of function mutations in genes encoding TCA cycle enzymes such as succinate dehydrogenase (SDH) and isocitrate dehydrogenase (IDH) respectively, exhibit an accumulation of the oncometabolites succinate or 2-hydroxyglutarate (2-HG). Both promote tumorigenesis and metastasis formation by epigenetically initiating EMT as inhibitors of a-KG dependent dioxygenases and by their release into the TME (236). Succinate induces macrophage polarization into M2-like tumor-associated macrophages *via* succinate receptor activation and the PI3K-HIF-1α axis promoting metastasis (237). 2-HG is released by IDH-negative cancer cells and is proposed to affect T-cells as IDH-negative tumors display a significantly lower T-cell infiltration compared to IDH-wildtype tumors (238, 239). 2-HG uptake induces HIF-1α destabilization and a shift towards OXPHOS in T-cells which is associated with lower levels of T-helper cells and a higher Treg abundance (217). In mice, CD4^+^ T cells display a decreased secretion of cytokines under hypoxia and 2-HG treatment (240). This exemplifies how the tumor metabolism participates in the shaping of its protective niche and how this reduces its radiotherapy response.

### TME Involvement in Metabolic Rewiring of Cancer Stem Cells

The dynamic TME leads, amongst others, to a high heterogeneity of metabolic profiles within tumors (241). Particularly, differences between normal cancer and cancer stem cell populations need to be considered for efficient therapeutic interventions as cancer stem cells (CSCs) drive cancer progression and recurrence. Mostly, glycolysis-dependent CSCs have been observed but there are also reports about mitochondrial driven CSCs, which is likely to depend on the genetic background, TME and the proliferative capacity (242–248). CSCs seem to be metabolically flexible, switching from OXPHOS to glycolysis or vice versa upon inhibition of one of these pathways (248). Moreover, evidence suggests that the metabolic profile between normal cancer cells and CSCs varies so that effective therapies and relapse prevention potentially requires the interference with different pathways (248–251). Highly aggressive cancers such as triple negative breast cancer or glioblastoma are suggested to exhibit CSC populations and to mainly rely on a mitochondrial energy metabolism (251–254). Therefore, it may be necessary to combine mitochondrial and glycolysis metabolism inhibition, in order reduce the potential radioresistance and have a successful treatment.

## Improving Radiotherapy Response by Tumor Sensitization

**Figure 1:**
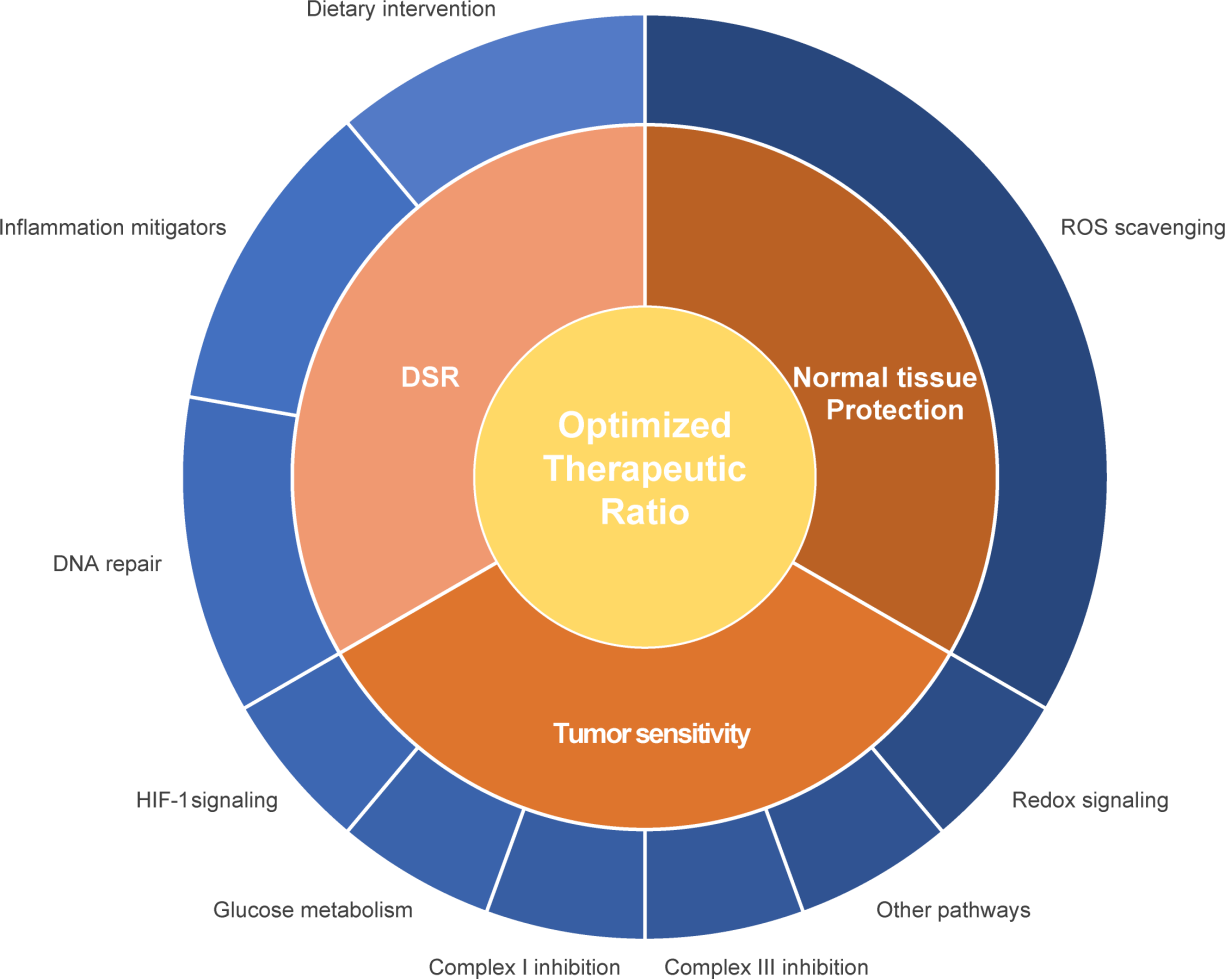
Schematic representation of different interventions to improve the therapeutic ratio. Interventions can either improve the radiosensitivity of the primary tumor or metastatic lesion or protect the healthy tissue. Influencing the differential stress response between tumor and normal healthy tissue can contribute to an enhanced therapeutic ratio. Different pathways and proteins can be influenced in relationship to enhancement of the therapeutic ratio, such as mitochondrial and glucose metabolism, ROS scavenging and redox signaling, hypoxia response, DNA repair capacity and inflammation.

Radiosensitizers aim to improve local tumor control and curation by enhancing radiotherapy-induced mitotic catastrophe of tumor cells without affecting normal cells. Different strategies to enhance radiosensitivity have been explored, such as 1) the physical amplification of the irradiation intensity e.g. by nanoparticles (255), 2) interventions to selectively enhance the radiation-induced ROS production for instance by increasing intracellular oxygen levels (175) and 3) suppression of survival pathways such as ROS degradation pathways or DNA repair pathways (175, 256). As metabolism and radiation response are linked, modulation of the cellular metabolism is another promising strategy to increase tumor’s radiosensitivity (**Figure 1**; **Table 1**).

### Modulation of HIF-1 Signaling

Mutations in the TCA cycle enzymes IDH, SDH and fumarate hydratase (FH) lead to HIF-1 accumulation (257–259). Tumors with mutations causing SDH or FH deficiency exhibit TCA cycle disruption and a disbalanced redox status caused by a disrupted NADPH-recycling. Due to these mutations levels of succinate and fumarate augment and thereby influence cellular metabolism also through phosphorylation-mediated downregulation of pyruvate dehydrogenase (260). Next to hypoxia, also mutations in SDH, FH and IDH are involved in EMT of cells and contribute to the formation of metastasis. Furthermore, these enzymes enhance the metabolic shift towards glycolysis, as they also inhibit prolyl hydroxylases leading to stabilization of HIF-1 (261, 262). Therefore, compounds interfering with HIF-1 signaling might enhance radiosensitivity.

The radiosensitizer deguelin is a rotenoid naturally produced by *Leguminosae* and has been proposed to augment the tumor chemo- and radiotherapy response (263). It targets multiple signaling pathways including STAT3, c-myc and E-cadherin to de-regulate proliferation, angiogenesis and metastasis capability. Furthermore, deguelin facilitates apoptosis by promoting cell cycle arrest and Akt inhibition (20, 264, 265). Inactivation of Akt destabilized the anti-apoptosis factors XIAP, Mcl-1, and survivin, downregulated HIF-1 signaling and reduced hexokinase expression. The deguelin-caused Akt dysregulation has been shown to radiosensitize breast cancer cells, associated with increased G_2_/M- arrest and caspase-dependent apoptosis (263). The mechanism of selective apoptosis induction of deguelin is unclear, but supposedly different caspase levels in normal and cancer cells influence the selectivity of deguelin (266). The use of deguelin is currently limited due to its assumed function as OXPHOS Complex I (CI) inhibitor (267). Caboni et al. demonstrated that rats receiving subcutaneous deguelin doses developed Parkinson’s disease-like syndrome (267). *In vitro* results suggested similar molecular effects of the deguelin derivative SH-14 but less toxicity and higher solubility which potentially could be an alternative (21).

Vandetanib, a FDA-approved medullary thyroid cancer therapy that inhibits EGFR (22), impairs the HIF-1 pathway by targeting the mTOR–HIF-1α–VEGF signaling axis in breast cancer cells (23) and increases survival in advanced medullary thyroid carcinoma (268). Vandetanib is able to radiosensitize and improve overall survival in xenografts (24). Phase I clinical trials proved Vandetanib’s safety in a (chemo)radiotherapy regimen for head and neck cancers (25) and brain metastasis of melanomas, although without differences for progression free survival (PFS) or overall survival (OS) (26). Additionally, adult SDH_mut_ gastrointestinal stromal tumor patients displayed treatment-related toxicities without partial or complete responses (27). The alkaloid Berberine also downregulated HIF-1-α and its downstream target vascular endothelial growth factor (VEGF) and reduced tumor invasiveness resulting in an improved radiotherapy response (28, 29, 269). Furthermore, berberine treatment results in decreased levels of RAD51, part of the homologous recombination mediated repair of irradiation-induced DSBs (30, 270) and induces a dose-dependent cell cycle arrest (31). Similarly, succinate and fumarate can, as both are competitive inhibitors of α-KG-dependent dioxygenases which control chromatin-methylation status (e.g. histone- and DNA-demethylases) (271), influence gene expression and DNA‑repair mechanisms; such as hypermethylation of the O6-methylguanine-DNA methyltransferase promoter (272–274). Also, ginsenoside Rg3, a ginseng extract, modulates HIF-1α stabilization, VEGF expression and NF-κB activation upon hypoxia exposure and sensitizes tumor cell lines and xenograft to radiation (32, 33).

### Altering Cellular Metabolism

The metabolism of malignant cells, especially pathways which are involved in the utilization of carbon as these are crucial for maintenance of cancer cell survival and proliferation, can be targeted directly. Prominent targets are the glucose metabolism, mitochondrial metabolism and antioxidant metabolism.

#### Glucose Metabolism Targeting

Glucose has a central function in the cellular metabolism and therefore several compounds have been developed which target the glucose import to reduce the glycolytic rate. BAY-876 is a highly selective small molecule inhibitor of the glucose transporter 1 (GLUT1), upregulated in many tumor cells and predictive for poor survival (34, 275–277). Moreover, BAY-876 demonstrated sensitizing effects towards cisplatin treatment in esophageal cancer cell lines (35). Its usefulness as a radiosensitizer needs to be investigated. WZB117, another promising GLUT1 inhibitor, re-sensitizes therapy-resistant breast cancer cells to radiotherapy and demonstrates anti-cancer effects in glioblastoma cells (36, 37).

The glucose analogue 2-deoxyglucose (2-DG) influences the radiotherapy response of cancer cells by interfering with glycolysis (38–40). Glucose and 2-DG compete for GLUT-mediated transmembrane transport. Both molecules are phosphorylated by hexokinase (HK) followed by phosphoglucose isomerase dependent metabolism, only possible for glucose. 2-DG, however, is not sensitive for the enzyme resulting in its accumulation and inhibition of HK. 2‑DG mediated downregulation of glycolysis therefore reduces cell growth and proliferation of radioresistant cells (41) and reduces angiogenesis and invasiveness of tumor cells (278). However, the mechanism of 2-DG-mediated cellular radiosensitivity remains to be elucidated. Studies proposed that 2‑DG reversed the radiation-induced cell cycle arrest which may contribute to later occurring cell death by mitotic catastrophe (38). Additionally, 2-DG-mediated disbalance of the cellular redox balance seems to promote radiation-induced cell death. Concurrent radiotherapy and 2-DG treatment led to 50% reduction of GSH levels while thiol-antioxidants reversed the 2-DG-induced radiosensitizing effects (39). Rashmi et al. showed that co‑treatment with GSH (buthionine sulphoximine) and thioredoxin synthesis (auranofin) inhibitors enhanced the radiosensitizing effects of 2-DG *via* adenosine monophosphate-activated protein kinase (AMPK) stimulation and subsequently autophagic cell death (41).

Treatment with MLN4924, a SCF E3 ligase inhibitor, synergized with 2-DG and radiotherapy in breast cancer cells (279). On the other hand, combination of 2-DG with radioimmunotherapy has antagonistic effects and indicated that the treatment effect may rely on glucose availability and level of hypoxia (280). WP1122 is an analogue of 2-DG having an extended half‑life and good oral bioavailability resulting into two-fold higher plasma concentrations compared to 2-DG and is capable of passing the blood-brain barrier. However, its effect on radiosensitivity remain to be elucidated (281).

Lonidamine 1-(2,4-dichlorobenzyl)-1H-indazole-3-carboxylic acid interferes with cellular metabolism in a very diverse manner. It can inhibit glycolysis *via* interference with hexokinase, contributing to intracellular lactate accumulation. Lonidamine also inhibits malate and fumarate formation in the citric acid cycle and it influences the ETC function by disturbing the mitochondrial transmembrane potential through inhibition of Complex II (CII) and the pentose phosphate pathway causing a decreased NADPH and glutathione pool (42, 282). Clinical studies in Head and Neck cancer showed improved survival rates (283). However, a prospective clinical trial for unresectable non-small-cell lung carcinoma (NSCLC) was negative for lonidamine in combination with radiotherapy versus radiotherapy alone (284). The lack of radiosensitization may be explained by the wide range of metabolic profiles observed in NSCLC (42, 43), although also the shift to the mitochondrial respiration due to potential tumor metabolic flexibility could be a potential explanation (285, 286). Also, lonidamine did not show any clinical effects in a large randomized trial in combination with chemotherapy (NCT00237536) (44).

#### Mitochondrial Metabolism Targeting

Beyond targeting glycolysis, the selective disruption of the mitochondrial metabolism might be another interesting approach. Devimistat (CPI-613), which recently received FDA fast-track designation for metastatic pancreatic cancer (NCT03504423), is a lipoate-analogue which dysregulates the TCA cycle through inhibition of the pyruvate dehydrogenase (PDH) and ROS induced inhibition of ketoglutarate dehydrogenase (KGDH) (45, 287). CPI-613 also activates AMPK signaling *via* stimulated ROS production (e.g. at KGDH), resulting in deactivation of acetyl-carboxylase and FA-synthesis (288). Combination of CPI-613 with PDK1 activators led to a reduced mitochondrial membrane potential and stimulated mitochondrial autophagy (289). Demonstrating tolerable side effects, CPI-613 is now under investigation in combination with different chemotherapeutics in phase 2/3 trials, but its effect in combination with radiotherapy is yet to be examined (46, 290, 291). Similarly, FH535, and its analogue Y3, disrupts the mitochondrial membrane potential which results in a decreased ATP production, induces apoptosis and reduces migration and invasiveness. FH535 has shown promising results *in vitro* and *in vivo* for different tumor types however, to date no clinical studies are available (47, 48, 292–295).

Also, IDH has been investigated as target for metabolic interventions, as it is frequently mutated in many aggressive cancers such as gliomas and AML (296, 297). Next to TCA cycle and redox-balance disruption, gain of function IDH mutations result in the production of the oncometabolite (D)-2-HG, which inhibits α-KG-dependent dioxygenases and thus, influencing the gene expression of metabolism regulators such as TP53-induced glycolysis and apoptosis regulator (TIGAR) (298). TIGAR regulates the cellular NADPH levels by controlling PPP supply with the glycolytic glucose-6-phosphate, which is required for the production of ROS scavengers as reviewed by Trachootham et al. (299). Cells with low TIGAR levels are proposed to be more susceptible to radiation, as ROS scavenging could be influenced (298, 300). Furthermore, reduced DNA repair was observed in presence of IDH_mut_, though inhibition of IDH1/2_mut_ did neither alter the radiation sensitivity nor radiation resistance of chondrosarcoma cell lines (298, 301). Comparing the treatment response between IDH_wt_ and IDH_mut_ AML and glioma patients upon salvage and chemoradiotherapy suggested an IDH mutation status independent treatment response (302, 303). However, targeting these variants specifically also has shown promising results. Ivosidenib and Enasidenib (AG-221) inhibit IDH1_mut_ and IDH2_mut_ respectively and are both FDA approved for treatment of acute or refractory AML (49, 50) and reduce invasiveness (109). Especially patients, who are not eligible for cytotoxic treatments, can benefit (51). However, adaptive IDH mutations can result in resistance to Enasidenib and Ivosidenib, causing a decrease in glioma treatment efficacy (52, 304). Therefore, other IDH inhibitors are under development. Vorasidenib has been shown to be a good candidate for glioma treatment as it passes the blood-brain-barrier (52, 305). Its safety and efficacy is currently investigated in multiple clinical trials (53, 306). Furthermore, the IDH1_mut_ inhibitor BAY-1436032 delays xenograft growth (54) and has also entered clinical trials (55, 307).

Also, inhibitors of different OXPHOS complexes are capable to enhance radiation response, this related to improved tumor reoxygenation. As they increase intracellular oxygen availability due to inhibition of OXPHOS, a decreased oxygen consumption may result in higher oxygen levels eventually re-sensitizing tumors to radiotherapy (308). Consequently, many OXPHOS inhibitors are tested for their potential radiosensitizing effects of tumors.

##### Complex I Inhibitors

The two CI (NADH:ubiquinone oxidoreductase) inhibitors metformin and papaverine are FDA-approved for non-cancerous diseases and are currently investigated for their radiosensitizing properties. Metformin is an anti-diabetic treatment and has already shown anti-cancer efficacy (309) and the potential to reduce metastatic potential (310, 311). Metformin was described to target the IGF receptor and consequently, indirectly mediates the downregulation of PI3K/Akt signaling (56, 312, 313). Additionally, metformin inhibits CI of the ETC resulting in a reduced ATP/AMP ratio (56, 313) and stimulates AMPK to promote cell cycle arrest and autophagy (314, 315). While impairing OXPHOS function, metformin administration results in oxygen accumulation and subsequently destabilization of (HIF-1-α) (316–318) and favors the formation of ROS after irradiation (319). Several *in vitro* studies suggested that metformin enhances the radiosensitivity of cancer cells. Zannella et al. demonstrated that metformin treatment prior to radiotherapy increased the intra-tumor oxygen levels in colorectal carcinoma xenografts, which enhanced the radiotherapy response (320). In contrast, De Bruycker et al. stated an anti-hypoxic effect of metformin in colorectal cancer xenografts, but a metformin-mediated enhanced radiotherapy response was not observed (321). These contradicting reports about metformin’s radiosensitizing effects may be caused by different experimental conditions since it is suggested that metformin only exerts its radiosensitizing effects in p53 mutated genetic contexts (322). Indeed, differences in mutations between HCT116 and Colo205 corroborate these findings (320, 321).

Completed Phase I trials of metformin combined with radiochemotherapy in head and neck squamous cell carcinoma patients are promising with a two years OS of 90%, a PFS of 84% and manageable toxicity, compared to historical control rates of 80% (OS) and 65% (PFS), although only a small cohort was included (57). Currently, metformin is investigated in several phase II trials in combination with different treatments (NCT02945813, NCT04275713, NCT02186847). Metformin’s tissue-specific uptake depends on the expression of organic cation transporter 1 (OCT1) transporters, explaining its low bioavailability (58, 323). The related biguanide, but more lipophilic, phenformin is suggested to have a higher bioavailability, does not rely on OCT1 expression for uptake and has a similar effect on cellular metabolism as metformin (58, 324, 325). However, it has been redrawn from the marked in the 1970s as it causes lactic acidosis in diabetic patients (326). Nevertheless, it may be a promising anti-cancer drug since it re-sensitizes resistant cells to chemo‑ and radiotherapy (327, 328) and synergizes with chemotherapeutics allowing a lower dosage of chemotherapeutics (329). Its safety as an anti-cancer drug is currently being investigated in phase I trials (59).

Papaverine (PPV) inhibits phosphodiesterase 10A (PDE10A) and is an FDA-approved vasospasm treatment (60). As it also targets CI of the ETC, it might exhibit anti-tumor properties. PPV sensitizes cells and xenografts to radiation, explained by a decreased OCR (61). The PPV derivative SMV-32 has been developed as a specific CI inhibitor, without affecting PDE10A activity. Its administration results in decreased tumor hypoxia leading to an improved radiotherapy response with acceptable toxicity profile in xenografts (61). Currently, the safety and tolerability of SMV-32 is assessed in a Phase I trial (NCT03824327).

Next to metformin and PPV, the small molecule CI inhibitors BAY 87-2243 and IACS-010759 presented anti-cancer effects *in vitro* and *in vivo* (330–332). BAY 87-2243 exhibited radiosensitizing effects in xenografts and is suggested to synergize with serine-threonine kinase inhibitors in BRAF_mut_ xenografts (333, 334). A phase 1 study with BAY 87-2243 was initiated in 2011 but terminated with an unclear status (NCT01297530). IACS-010759 has entered phase 1 trials investigating its safety and tolerability in solid tumors, lymphoma and leukemia (NCT03291938, NCT02882321). However, its treatment sensitizing effects remains to be elucidated.

##### Complex III Inhibitors

Atovaquone (ATO) inhibits Complex III (CIII) of the ETC and is FDA-approved as an anti-malaria treatment. In xenografts, radiotherapy combined with ATO delays tumor growth, which has been associated with reduced hypoxia. ATO-treated xenografts display a significant higher reduction in OCR as compared to metformin treatment (73.7% vs 43.1%). Additionally, ATO targeted the pyrimidine synthesis enzyme dihydroorotate dehydrogenase (DHODH) (62). It is assumed that ATO mainly exerts its radiosensitizing effects *via* CIII inhibition since the potency against DHODH was markedly lower (335–337). The results of a first phase 1 study investigating the anti-hypoxic effects of ATO in NSCLC patients are yet to be published (63). Hypoxia mitigating effects of inhibitors of the ETC complexes II, IV and V are also investigated (338–346).

The small molecule inhibitor pyrazinib (P3) reduces OCR and extracellular acidification rate (ECAR) in zebrafish and is associated with an improved radiosensitivity in esophageal cancer cell lines, although the precise mechanism remains to be elucidated. Buckley at al. hypothesized that the P3 target lies upstream of glycolysis and OXPHOS. Furthermore, they suggested that P3 acts as a radiosensitizer under hypoxic conditions (64).

##### Other Metabolic Pathways

Modulation of the arginine metabolism is suggested to increase the cellular sensitivity to anti-cancer therapies. ADI-PEG 20, a chimera of the arginine deiminase (ADI) and polyethylene glycol (PEG) (347), consumes the cellular arginine by catalyzing the conversion of arginine to citrulline, a clinical marker for radiation-induced tissue toxicity (348). Moreover, ASS1-deficient pancreatic cancer cells displayed enhanced radiosensitivity through arginine depletion after ADI-PEG 20 treatment (65).

Arginino succinate synthetase (ASS1) produces endogenous arginine from citrulline, however, many cancer cells exhibit arginine auxotrophy e.g., due to ASS1 deficiency (349–351). Being the precursor for various molecules including pyrimidines, cells display an enhanced vulnerability in arginine-depleted environments (352, 353). ASS1-deficient cancer cells show synthetic lethality and an inhibited Warburg phenotype under arginine depletion (66). Phase 1 trials in combination with chemotherapeutics in different cancer types reveal tolerable side effects and hint towards a treatment response (354, 355). Interestingly the response depends on the ASS1 status (67, 356). Currently, phase 2 trials investigate the combination of different chemotherapeutics in several cancer types including pleural mesothelioma and hepatocarcinoma [NCT02102022, NCT02709512 (357, 358)].

Manipulation of the FA metabolism can increase the efficacy of chemotherapy and radiotherapy. Different compounds modulating FA metabolism have been investigated for their potential applications in cancer treatment by either improving therapeutic responses, reducing tumor progression or metastatic formation. The FDA-approved obesity therapeutic agent orlistat inhibits FA synthase blocking a crucial anabolic pathway (68) and is associated with an enhanced response to chemotherapeutic treatments and radiotherapy and experimental reduced metastasis formation (359–361). Fenofibrate (FEN), a PPARα agonist, reverses metabolic reprogramming of cancer cells leading to enhanced radiosensitivity *in vitro* and *in vivo* under hypoxic conditions (69, 70) and also reduces metastasis formation of melanomas (362). FEN activates AMPK signaling and FA oxidation *via* carnitine palmitoyltransferase 1 (CPT1) and inhibits PI3K/Akt signaling resulting in reduced hexokinase 2 levels and glycolysis (71). FEN also prevents HIF-1 stabilization (69, 70) and the disruption of the HIF-1α/VEGF axis may contribute, when combined with radiotherapy, to G_2_/M-phase arrest (70, 363).

### Influencing Redox Signaling in Tumors

The indirect ROS-mediated DNA damage is believed to be a main factor to activate cell death pathways upon radiotherapy (364, 365). Since proliferating cells have a naturally higher ROS production, cancer cells need to compensate this by simultaneously increasing the production of protective antioxidants (366). Hence, the impairment of anti-oxidants increases the radiotherapy effect (367). Moreover, excessive ROS levels induced by mutations and/or radiotherapy are suggested to induce ferroptosis, a phenomenon describing the Fe^2+^-dependent cell death induced by high levels of peroxidized lipids (368). Radiotherapy induced ferroptosis by further enhancing ROS formation and activating long-chain-fatty-acid CoA ligase 4 (ASCL4) to promote the formation of polyunsaturated fatty acid phospholipids (PUFA-PL) (368). Contrary, Lei et al. has described reduced ferroptosis upon irradiation, depending on the genetic background through induction of glutathione peroxidase (GPX) and SCL7A11 (part of X_c_-antiporter), which increased the reduction equivalent pool (368). Others however reported that radiotherapy suppresses SLC7A11 expression indirectly *via* ATM downregulation and thus limits the import of cysteine, a GSH precursor (369). Therefore, manipulation of this pathway is associated with radiation sensitivity. Firstly, it is stated that the inhibition of X_c_ or GPX induces ferroptosis and sensitizes cell lines and xenografts to radiotherapy due to a lack of glutathione (GSH) (369–371). Second, many neoplasms increase their glutaminolysis e.g., by oncogene (*myc*)-driven glutaminase (GLS) overexpression.

GLS produces the GSH precursor glutamate and its inhibition resulted in tumor growth delay (252). Although several GLS inhibitors synergize with anti-cancer treatments (72, 372, 373), their application in clinical practice is often limited due to its low bioavailability (374). Telaglenastat (CB-839) demonstrates improved bioavailability and synergizes in xenografts with radio- and chemotherapy (72, 73). In clinical trials combined with chemo- and immunotherapeutics it has demonstrated promising results with tolerable side effects (74, 75, 375, 376). Thirdly, supplementation of PUFAs pre-, during or post radiotherapy demonstrates synergism in rat astrocytoma cells and xenografts (377–379) as a result of COX2 downregulation and reduced vascularization (379).

Moreover, inhibitors of enzymes maintaining the redox balance, such as thioredoxin reductase (TrXR), are considered potential targets. Auranofin is a TrXR inhibitor already implemented as arthritis therapy. Its administration sensitized cancer cells to radiation preventing ROS degradation (76).

## Protecting Normal Tissue During Radiotherapy

Radiotherapy cannot always be applied in curative doses as damage in the adjacent normal tissues is dose limiting. Additionally, the bystander effect, i.e. the phenomenon that irradiated cells negatively influence non-irradiated cells, also limits radiotherapy efficacy (380). Normal tissue cellular damage affects quality of life post radiotherapy tremendously. Therefore, it is crucial to identify strategies to protect normal tissues without reducing the efficacy of radiotherapy. Interventions aim to selectively augment the normal tissue cellular survival pathways, e.g., ROS depletion and DNA repair pathways counteracting the radiation-induced ROS burst and DNA lesions (**Figure 1**). The differential metabolic pattern between malignant and healthy cells represents a suitable target to modulate these pathways selectively within normal tissues. The NF-κB signaling pathway plays a pivotal role in the response of tissues to radiation. Its activation results in stimulation of inflammatory and apoptosis pathways in normal tissues, promoting tissue damage and cell death and therefore limits the applied radiotherapy dose (381). Consequently, many interventions aim to modulate NF-κB signaling counteracting the unwanted side effects. Here, we focus on radioprotectors influencing the metabolic pathways attenuating radiotherapy-induced effects in normal tissues. These can be broadly classified based on their effect into radical scavengers, inflammation mitigators and DNA repair modulators (**Table 2**).

### Radical Scavengers

The IR-induced ROS burst diminishes levels of endogenous ROS scavengers which has to be recycled. Therefore, agents that chemically reduce ROS or activate pathways/molecules facilitating ROS depletion selectively in normal cells can ameliorate radiation-induced damage (**Figure 1**).

Amifostine, a ROS scavenger, is FDA‑approved for its radioprotective effects in ovarian and head and neck cancer patients. It is activated *via* dephosphorylation by alkaline phosphatases, which are highly expressed on the surface of normal cells; thus, amifostine accumulates preferentially in normal tissues (77, 382). The precise mechanism of action is unknown, but several have been proposed. Firstly, its active form harbors free thiol groups to reduce intracellular ROS-induced damage and to prevent delayed genomic instability in cells (383, 384). Second, amifostine reduces oxygen consumption and induces HIF-1, which correlated with its radioprotective effect. Koukourakis et al. argued that this oxygen-depletion may result from the reaction between oxygen and the free thiol, which leads to hypoxia for a short time, inducing HIF-1. However, the precise mechanism of amifostine-induced HIF-1 stabilization is unclear (385). Thirdly, its administration was associated with DNA condensation reducing damage efficiency (386). Fourthly, amifostine may alter lipid membrane dynamics affecting membrane proteins and therefore it can influence downstream signaling pathways of transmembrane receptors (387).

The results of clinical trials using amifostine are ambiguous, some describing radioprotective effects whereas some did not observe any effect (388). Prostate cancer patients receiving radiotherapy combined with amifostine produced significant improvements in acute and late bowel quality of life (up to 1 year after therapy), measured using the Expanded Prostate Cancer Index Composite (EPIC) score. Differences between dose groups are evident from week 7 onwards. The RTOG gastrointestinal (GI) toxicity score mirrored these results stating a lower GI toxicity in probands receiving a higher amifostine dose, although in a non-significant manner (78). Dose-related adverse effects induced by amifostine include nausea and hypotension and seems to be affected by the route of administration. Bardet et al. compared daily intravenous (iv) with subcutaneous (sc) administration of amifostine prior to radiotherapy and reported higher occurrence of hypertension upon iv injection, while a higher number of patients suffering from xerostomia upon sc administration (79). Another study observed lower rectal mucositis after intrarectal amifostine administration before radiotherapy, while sc application resulted in a lower urinary toxicity (389). Aminothiol analogues of amifostine, such as PrC-210, demonstrate less adverse effects in rodents and significantly prolongs the survival of P53^−/−^ mice upon irradiation (80, 81, 390).

Another possibility to mitigate radiation-induced damage of normal tissue is the intracellular stabilization of ROS scavenging enzyme levels, which are inactivated after irradiation due to a ROS burst and infiltration of neutrophils. Administration of DNA-sequences encoding ROS-scavenging enzymes ameliorate radiotherapy-induced cell damage (391). Ingestion of manganese superoxide dismutase- plasmid liposomes (MnSOD-PL) has been shown to prolong mice survival after total body irradiation without protecting the tumor (392, 393). Mitochondrial localization of MnSOD-PL seems to be crucial for its efficient radioprotection (82). In order to further exploit anti-oxidant therapies, fusion peptides (nitroxides) with a high mitochondrial localization rate have been developed. JP4-039 stabilizes mitochondrial ROS scavenger levels reducing radiation induced anti-oxidant depletion and mitigating radiotherapy-induced damage in normal tissues (83, 84, 394), while a tumor-protective effect has been excluded (84, 395). Other studies have shown protective effects on normal tissues by recovery of stem cell function and differentiation (396, 397). Di‑seleno‑di‑propionic acid (DSePA) is able to maintain the levels of ROS scavengers in irradiated mice, mitigates the ROS burst, reduces radiation‑induced expression of pro-inflammatory cytokines, oxidative stress, pneumonitis and inflammation responses (85, 398). In mice oral administration is effective, contributing to potential improvement of patient compliance (85). The effects on normal tissue may be explained by the limited uptake of DSePA in tumors and the preferential accumulation in lung, intestine and kidney (86). Therefore, it is mainly investigated as co-therapy for cancer patients facing upper body radiotherapy.

### Inflammation Mitigators

Other approaches aim to exploit the differential response of normal and cancer cells to stress (DSR) (**Figure 1**). These treatments amplify radiation-induced stress in tumor tissues whereas they ameliorate the normal tissue reaction. TNFα activation mediates the expression of NADPH-oxidases, promoting oxidative stress which damages healthy tissues and severely affects the patient’s quality of life. Therefore, these molecular pathways are investigated as a potential treatment target.

Cyclooxygenase-2 (COX2), involved in the prostaglandin synthesis, is overexpressed in many cancers and is associated with chemoradioresistance. Radiotherapy further enhances its expression *via* NF-κB signaling pathways. Consequently, blocking of COX2 and NF-κB-signaling improves radiotherapy response (399). Elshawi et al. demonstrated in mice that COX2 inhibition with a benzopyran compound mitigated radiation-induced NF-κB and COX2 activity. The treatment also ameliorated other radiotherapy-induced effects such as the increase of cytokines and decrease of liver enzymes (400). Celecoxib, another COX2 inhibitor, reduces radiation-induced skin toxicity in mice (401), which has been confirmed by other studies, reporting that celecoxib treatment enhances radiosensitization and reduces tumor growth (399, 402, 403). The results of a phase 2 trial investigating the effects of celecoxib combined with radiochemotherapy in NSCLC patients however were inconclusive (404). A second study treating colorectal cancer patients with celecoxib and chemoradiation stated an ameliorating effect on skin toxicity compared to earlier studies (87).

Naturally occurring compounds such as ascorbic acid, curcumin, melatonin, caffeic acid phenol ester and vitamin E are associated with radioprotective effects on normal tissues whereas they stimulate the radiosensitivity of tumor tissues. High doses of vitamin E and ascorbic acid demonstrated radiosensitizing effects in multiple cancer types (88–91). Intravenous administered ascorbate increases the therapeutic ratio by increasing radiation-induced DNA damage in pancreatic tumors and simultaneously decreasing DNA lesions in a non-carcinogenic tissues (90). Furthermore, ascorbate is suggested to downregulate the expression of the ROS scavenger MnSOD in cancer cells by controlling the NF-κB component RelB, whereas it upregulates MnSOD expression in normal cells (91). In agreement, Alexander et al. reported that supplementation of ascorbate mitigates the decrease of ROS scavengers in normal tissue of mice (90). Furthermore, they state in phase 1 trials that ascorbate supplemented to radiochemotherapy for pancreatic cancer patients is safe and potentially enhances treatment efficacy (90).

Curcumin blocks NF-κB signaling by inhibiting IκBα dissociation and TNFα‑dependent pathway activation (92). In agreement, Cho et al. described in rats that curcumin counteracts the IR-induced TNFα expression and NF-κB translocation to the nucleus, which eventually alleviates radiotherapy-induced pneumonitis (405). This is substantiated by a study stating that curcumin reduced IL4 and NADPH-oxidase levels post-IR, which was associated with lower pneumonitis levels (406).

Melatonin (MLT) ameliorates radiation-induced oxidative stress by depleting hydroxyl radicals directly and by stimulating the activity of GPx and SOD, whereas it reduces the activity of ROS producing enzymes (NOS, NOX2/4) in normal cells (93, 407–409). Additionally, MLT downregulates NF-κB-signaling, mitigating an inflammatory response and enhances expression of DNA-repair genes contributing to genomic stability in normal cells (410–412). However, evidence implies that MLT combined with metformin exerted both synergizing and antagonizing effects in healthy rodents in a tissue-specific manner (94, 413). Findings in xenografts suggested that MLT exerts tumor-sensitizing effects by reducing DNA repair and stimulating OXPHOS in malignant cells intensifying the oxidative stress. Simultaneously it reverses the Warburg effect by potentially inhibiting mitochondrial PDK (414–416). Clinical trials on MLT however reported heterogeneous results. Onseng et al. examined the effects of MLT supplementation to chemoradiotherapy of head and neck cancer patients reporting a delayed onset of grade 3 oral mucositis (95), however without differences in mucositis incidence and quality of life. Treating breast cancer patients with a MLT-emulsion resulted in a lower dermatitis incidence (96). Post-radiotherapy MLT treatment is proposed to mitigate long-term radiation effects (417). Others did not observe synergistic effects with radiotherapy, although only upon comparison with controls from other studies (97).

Caffeic acid phenyl ester (CAPE) may exert its radiosensitizing effect in cancer cells by suppression of NF-κB-signaling and is associated with decreased glutathione-reductase levels and increased glutathione-peroxidase levels. As a result of this redox-imbalance ROS levels are increased (418, 419). Moreover, it re-sensitizes radiation-resistant breast cancer cells by impairing the DNA repair and thus, enhancing IR-induced genomic instability (420). In normal cells on the other hand, CAPE mitigates cellular oxidative stresses by enhancing ROS scavengers expression levels and by interference with radiation-induced NF‑κB‑signaling (98, 99). Also reduced expression of cytokines preventing fibrosis of lung tissue post-radiation has been observed (98).

Vitamin E and its derivates are proposed to cause a differential stress response between tumor and normal tissues (421). In combination with radiochemotherapy, vitamin E ameliorates treatment-induced mucositis in head and neck cancer patients (89). Especially, the vitamin E derivative γ-tocotrienol received attention as anti-cancer treatment having based on its superior antioxidant capacity. Kumar et al. reported that the lipid peroxidation levels in tumor tissue increases under γ-tocotrienol administration, whereas specific adjacent tissues are protected (88). Moreover, they observe a reduction in radiation-induced lipid peroxidation in a tissue-dependent manner. The relatively low bioavailability of tocotrienols may be enhanced by optimizing administration schedule (422).

### DNA Repair/Genomic Maintenance

Radiation and ROS-induced DSBs activate ataxia telangiectasia mutated (ATM) signaling promoting p53-induced cell cycle arrest and epigenetic marking of DSBs to facilitate DNA repair plays a central role in the decision whether to promote survival or induce cell death to prevent tumorigenesis (423, 424). It is proposed that polyphenol resveratrol (RSV) provokes a differential stress response and radiosensitizes cancer cells *via* interference with ATM-signaling and apoptosis cascade. It inhibits the expression of Mcl-1 by downregulation of STAT3 signaling (100, 425). Vendrely et al. demonstrated that RSV combined with capsaicin and radiotherapy inhibits ATM-based DNA repair in pancreatic cancer cells and increases phosphorylated p53. Activation of p53 results in cell cycle arrest and promotes apoptosis by increasing the Bcl-2/Bax ratio (101). Several other studies substantiated this, reporting RSV-associated G_0_/G_1_ arrest (426), a cell cycle phase with higher radiosensitivity compared to S-phase cells (426, 427).

The tumor suppressor p53 is required for maintenance of a G_1_ arrest and determines the cellular fate. Serine/threonine kinase (Akt) influences p53-mediated effects by decreasing its pro-apoptotic effects. Thus, high pAkt levels favor cellular survival whereas low pAkt levels favor apoptosis (428). Interestingly, RSV is associated with downregulation of E2F1 and its downstream target pAkt (429–431). Multiple studies on RSV and radiotherapy have demonstrated its radiosensitizing effects in several carcinoma cells and *in vivo* (101, 429, 432–434). On the other hand, RSV and 3,3′-diindolylmethane combination treatment prior to radiotherapy stabilizes the levels of radical scavenging enzymes, reduces genomic alterations such as micronuclei formation and mitigated radiation-induced normal tissue damage in mice (435–437). Moreover, it directly activates ATM signaling in a context of oxidative stress, which may explain the opposing effect in cancer and normal cells (438). However, its clinical use is limited by its metabolic instability, low bioavailability (439) and photosensitivity (440). More stable RSV analogues such as HS-1793 have been associated with an improved radiotherapy response in xenografts by modulating the anti-cancer immune response (102).

## Dietary Interventions Influencing the Radiotherapy Response

Not only drugs and compounds could be of benefit to improve the therapeutic window, also dietary interventions could contribute to a more favorable treatment outcome (**Figure 1**, **Table 2**). Malignant cells display different metabolic needs than normal cells, because of their uncontrolled proliferative potential and often impaired OXPHOS, which results in elevated ROS levels. Short-term fasting (STF) and ketogenic diet (KD) exploit the difference in tolerability of oxidative stress between cancers cells and normal tissues by reducing global plasma glucose levels and increasing ketone body levels (441–443). A minimum of 24 h fasting prior treatment sensitized xenografts to radio-/chemotherapy (103, 104, 444). KD had a similar effect in xenografts exposed to radiochemotherapy and prolonged the overall survival co-occurring with enhanced protein oxidation, indicating that high ketone and low glucose levels amplified the ROS-induced damage in malignant cells (445). Moreover, KD may intensify energetic stress in tumors which display TCA cycle mutations, since these tumors are not able to utilize acetyl-CoA derived from the β-oxidation. Furthermore, evidence points towards a higher production of the oncometabolite 2-HG concurrent to increased β-oxidation in IDH1_mut_ glioblastoma cells (446).

Normal tissues react differently to glucose deprivation in presence of ketone bodies as they display a higher metabolic flexibility, reducing their proliferative potential to remain in G_0_-phase. Furthermore, they circumvent an energy deficit relying on FA-oxidation and OXPHOS (442). Abdelwahab et al. argues that there is no correlation between plasma glucose levels and cellular survival *in vivo*, indicating that metabolic reprogramming itself does not exert significant anti-tumor effects (442). Using pancreatic cancer xenografts, they demonstrate that 24 h fasting prior to radiotherapy prolongs survival and protects small intestinal stem cells optimizing the regeneration of adjacent, damaged tissues without tumor protection (104) Furthermore, they argue that this effect may occur due to a reduced apoptosis rate in fasted animals, since fasted animals display significantly lower cleaved caspase-3 levels. The combination of cisplatin treatment and caloric restriction also provoked a DSR in xenografts (447). Shi et al. demonstrates that fasting led to AMPK and ATM/p53 signaling in both normal and cancer cells (447). However, normal cells display increased levels of phosphorylated p53 resulting in G_0_/G_1_ phase arrest and less vulnerability to cisplatin treatment. Cancer cells maintain normal levels of phosphorylated p53 and cell cycle progression enhancing their sensitivity against cisplatin-induced damage compared to unfasted controls (447). Despite these results, the authors conclude that the introduction of caloric reduction into clinics is not advisable since many cancer patients already suffer from cachexia (104, 448).

KD mirrors the molecular effects of fasting, but does not enhance the cachexic phenotype of patients in phase 1 trials (443, 449, 450). These studies suggest that the combination of KD and radiotherapy synergizes in xenografts, but large cohort studies are currently lacking. Furthermore, they report that patient diet compliance is difficult with a lot of drop-out in these clinical studies. In addition, it is until now unclear what the best KD administration starting point before treatment is. A different route of administration *via* PEG tubes may facilitate treatment compliance (449). Another possibility may be mimicking the molecular effects using different drugs, such as metformin (104, 451). Cuyàs et al. reports an increase of ketone bodies and α-KG in HER2-positive breast cancer patients treated with metformin and chemotherapy (451).

## Conclusion and Perspectives

Modulating cellular metabolism to increase anti-cancer therapy efficacy is a powerful strategy, evidenced by the clinical implementation of some of these modulators. However, the utility of most of these metabolism-modulating compounds is limited due to low bioavailability, adverse and off-target effects. Adverse effects may occur less or more depending on the delivery route as seen for amifostine. Therefore, it is crucial to identify for every compound the optimal conditions for administration. Often alternative administration methods mitigate adverse effects and slightly enhance the bioavailability. Hence, attempts are made to develop derivatives and analogues of these compounds which mirror the effect of their parent compound and potentially reducing the binding to off-targets and subsequently reduce adverse effects. The success of metabolic interventions depends on the metabolic pattern of the primary tumor, metastatic lesions and the tumor’s micro-environment, which shows a large intra- and intercellular variability due to different nutritional requirements for proliferation/invasion and metastasis formation, and thus requires understanding and assessment of this pattern. Influencing the primary tumor’s metabolism could potentially also increase EMT, thereby causing a higher invasiveness potential or a more radioresistant phenotype of the metastasis.

As the tumor and TME metabolism are very dynamic processes, interactions between substrate availability and different metabolic pathways are very complex. There are close relationships reported between e.g. glycolysis, PPP, glutamine metabolism, FAO, TCA cycle, and OXPHOS as often substrates, by-products, and end-products often interact with multiple metabolic and signaling pathways. Rewiring the tumor’s metabolism is therefore very challenging. Combining rewiring metabolism with radiotherapy creates challenges and opportunities for successful implementation in clinical practice. Creating more therapeutic resistant tumors, increasing their metastatic potential, or induce adverse normal tissue effects needs to be prevented. Therefore, more research on this topic is needed to elucidate these risks.

Overall, the malignant, metabolic rewiring and its implications on treatment response is complex. However, first attempts exploiting this phenomenon demonstrate promising results to further optimize our current anti-cancer therapies and to improve the therapeutic window for patients.

## Author Contributions

Conceptualization of the review was performed by MG and LD. MG and EZ wrote the first draft. All authors revised and edited the manuscript. All authors contributed to the article and approved the submitted version.

## Funding

Financial support was provided by The Dutch Cancer Society (KWF UM 2015-7635).

## Conflict of Interest

The authors declare that the research was conducted in the absence of any commercial or financial relationships that could be construed as a potential conflict of interest.

## References

[1] Bray F Jemal A Grey N Ferlay J Forman D. Global cancer transitions according to the Human Development Index (2008-2030): A population-based study. Lancet Oncol (2012) 13(8):790–801. 10.1016/S1470-2045(12)70211-5 22658655

[2] Dillekas H Rogers MS Straume O. Are 90% of deaths from cancer caused by metastases? Cancer Med (2019) 8(12):5574–6. 10.1002/cam4.2474 PMC6745820 31397113

[3] Delaney G Jacob S Featherston C Barton M. The role of radiotherapy in cancer treatment: Estimating optimal utilization from a review of evidence-based clinical guidelines. Cancer (2005) 6:1129–37. 10.1002/cncr.21324 16080176

[4] Formenti SC Demaria S. Combining radiotherapy and cancer immunotherapy: a paradigm shift. J Natl Cancer Inst (2013) 105(4):256–65. 10.1093/jnci/djs629 PMC3576324 23291374

[5] Weichselbaum RR Liang H Deng L Fu YX. Radiotherapy and immunotherapy: a beneficial liaison? Nat Rev Clin Oncol (2017) 14(6):365–79. 10.1038/nrclinonc.2016.211 28094262

[6] Dovedi SJ Cheadle EJ Popple AL Poon E Morrow M Stewart R. Fractionated Radiation Therapy Stimulates Antitumor Immunity Mediated by Both Resident and Infiltrating Polyclonal T-cell Populations when Combined with PD-1 Blockade. Clin Cancer Res (2017) 23(18):5514–26. 10.1158/1078-0432.CCR-16-1673 28533222

[7] Vatner RE Cooper BT Vanpouille-Box C Demaria S Formenti SC. Combinations of immunotherapy and radiation in cancer therapy. Front Oncol (2014) 4:325. 10.3389/fonc.2014.00325 25506582 PMC4246656

[8] Kang J Demaria S Formenti S. Current clinical trials testing the combination of immunotherapy with radiotherapy. J Immunother Cancer (2016) 4:51. 10.1186/s40425-016-0156-7 27660705 PMC5028964

[9] Shaverdian N Lisberg AE Bornazyan K Veruttipong D Goldman JW Formenti SC. Previous radiotherapy and the clinical activity and toxicity of pembrolizumab in the treatment of non-small-cell lung cancer: a secondary analysis of the KEYNOTE-001 phase 1 trial. Lancet Oncol (2017) 18(7):895–903. 10.1016/S1470-2045(17)30380-7 28551359 PMC5538772

[10] Vanpouille-Box C Alard A Aryankalayil MJ Sarfraz Y Diamond JM Schneider RJ. DNA exonuclease Trex1 regulates radiotherapy-induced tumour immunogenicity. Nat Commun (2017) 8:15618. 10.1038/ncomms15618 28598415 PMC5472757

[11] Formenti SC Rudqvist NP Golden E Cooper B Wennerberg E Lhuillier C. Radiotherapy induces responses of lung cancer to CTLA-4 blockade. Nat Med (2018) 24(12):1845–51. 10.1038/s41591-018-0232-2 PMC6286242 30397353

[12] Stupp R Mason WP van den Bent MJ Weller M Fisher B Taphoorn MJB. Radiotherapy plus Concomitant and Adjuvant Temozolomide for Glioblastoma. New Engl J Med (2005) 352(10):987–96. 10.1056/NEJMoa043330 15758009

[13] Katanyoo K Tangjitgamol S Chongthanakorn M Tantivatana T Manusirivithaya S Rongsriyam K. Treatment outcomes of concurrent weekly carboplatin with radiation therapy in locally advanced cervical cancer patients. Gynecol Oncol (2011) 123(3):571–6. 10.1016/j.ygyno.2011.09.001 21955483

[14] Suntharalingam M Winter K Ilson D Dicker AP Kachnic L Konski A. Effect of the addition of cetuximab to paclitaxel, cisplatin, and radiation therapy for patients with esophageal cancer the NRG oncology rtog 0436 phase 3 randomized clinical trial. JAMA Oncol (2017) 3(11):1520–8. 10.1001/jamaoncol.2017.1598 PMC5710193 28687830

[15] Rose PG Bundy BN Watkins EB Thigpen JT Deppe G Maiman MA. Concurrent Cisplatin-Based Radiotherapy and Chemotherapy for Locally Advanced Cervical Cancer. New Engl J Med (1999) 340(15):1144–53. 10.1056/NEJM199904153401502 10202165

[16] Lawrence TS Eisbruch A Shewach DS. Gemcitabine-mediated radiosensitization. Semin Oncol (1997) 24(2 Suppl 7):S7–24–S7–8. 9194476

[17] Zindler JD Thomas CR Jr Hahn SM Hoffmann AL Troost EG Lambin P. Increasing the Therapeutic Ratio of Stereotactic Ablative Radiotherapy by Individualized Isotoxic Dose Prescription. J Natl Cancer Inst (2016) 108(2):djv305. 10.1093/jnci/djv305 26476075

[18] Zhao Y Butler EB Tan M. Targeting cellular metabolism to improve cancer therapeutics. Cell Death Dis (2013) 4:e532. 10.1038/cddis.2013.60 23470539 PMC3613838

[19] Citrin DE. Recent Developments in Radiotherapy. N Engl J Med (2017) 377(11):1065–75. 10.1056/NEJMra1608986 28902591

[20] Li W Gao F Ma X Wang R Dong X Wang W. Deguelin inhibits non-small cell lung cancer via down-regulating Hexokinases II-mediated glycolysis. Oncotarget (2017) 8(20):32586–99. 10.18632/oncotarget.15937 PMC5464811 28427230

[21] Kim WY Chang DJ Hennessy B Kang HJ Yoo J Han SH. A novel derivative of the natural agent deguelin for cancer chemoprevention and therapy. Cancer Prev Res (2008) 1(7):577–87. 10.1158/1940-6207.CAPR-08-0184 PMC2738643 19139008

[22] Fallahi P Ferrari SM Elia G Ragusa F Paparo SR Ruffilli I. Evaluating vandetanib in the treatment of medullary thyroid cancer: Patient-reported outcomes. Cancer Manag Res (2019)11:7893–907. 10.2147/CMAR.S127848 PMC6708888 31686907

[23] Li L Yu J Jiao S Wang W Zhang F Sun S. Vandetanib (ZD6474) induces antiangiogenesis through mTOR–HIF-1 alpha–VEGF signaling axis in breast cancer cells. OncoTargets Ther (2018) 11:8543–53. 10.2147/OTT.S175578 PMC6278704 30555244

[24] Znati S Carter R Vasquez M Westhorpe A Shahbakhti H Prince J. Radiosensitisation of Hepatocellular Carcinoma Cells by Vandetanib. Cancers (Basel) (2020) 12(7):1878. 10.3390/cancers12071878 PMC7408860 32668592

[25] Papadimitrakopoulou VA Frank SJ Cohen EW Hirsch FR Myers JN Heymach JV. Phase I study of vandetanib with radiation therapy with or without cisplatin in locally advanced head and neck squamous cell carcinoma. Head Neck (2016) 38(3):439–47. 10.1002/hed.23922 PMC4414661 25352401

[26] Gupta A Roberts C Tysoe F Goff M Nobes J Lester J. RADVAN: a randomised phase 2 trial of WBRT plus vandetanib for melanoma brain metastases - results and lessons learnt. Br J Cancer (2016) 115(10):1193–200. 10.1038/bjc.2016.318 PMC5104891 27711083

[27] Glod J Arnaldez FI Wiener L Spencer M Killian JK Meltzer P. A Phase II trial of vandetanib in children and adults with succinate dehydrogenase-deficient gastrointestinal stromal tumor. Clin Cancer Res (2019) 25(21):6302–8. 10.1158/1078-0432.CCR-19-0986 PMC6825553 31439578

[28] Yang X Yang B Cai J Zhang C Zhang Q Xu L. Berberine enhances radiosensitivity of esophageal squamous cancer by targeting HIF-1α in vitro and in vivo. Cancer Biol Ther (2013) 14(11):1068–73. 10.4161/cbt.26426 PMC3925662 24025355

[29] Zhang Q Zhang C Yang X Yang B Wang J Kang Y. Berberine inhibits the expression of hypoxia induction factor-1alpha and increases the radiosensitivity of prostate cancer. Diagn Pathol (2014) 9(1):98–8. 10.1186/1746-1596-9-98 PMC4051149 24886405

[30] Liu Q Jiang H Liu Z Wang Y Zhao M Hao C. Berberine Radiosensitizes Human Esophageal Cancer Cells by Downregulating Homologous Recombination Repair Protein RAD51. PloS One (2011) 6(8):e23427–7. 10.1371/journal.pone.0023427 PMC3152570 21858113

[31] Lu W Du S Wang J. Berberine inhibits the proliferation of prostate cancer cells and induces G0/G1 or G2/M phase arrest at different concentrations. Mol Med Rep (2015) 11(5):3920–4. 10.3892/mmr.2014.3139 25572870

[32] Wang J Tian L Khan MN Zhang L Chen Q Zhao Y. Ginsenoside Rg3 sensitizes hypoxic lung cancer cells to cisplatin via blocking of NF-kappaB mediated epithelial-mesenchymal transition and stemness. Cancer Lett (2018) 415:73–85. 10.1016/j.canlet.2017.11.037 29199005

[33] Wang L Li X Song YM Wang B Zhang FR Yang R. Ginsenoside Rg3 sensitizes human non-small cell lung cancer cells to gamma-radiation by targeting the nuclear factor-kappaB pathway. Mol Med Rep (2015) 12(1):609–14. 10.3892/mmr.2015.3397 25738799

[34] Siebeneicher H Cleve A Rehwinkel H Neuhaus R Heisler I Müller T. Identification and Optimization of the First Highly Selective GLUT1 Inhibitor BAY-876. ChemMedChem (2016) 11(20):2261–71. 10.1002/cmdc.201600276 PMC5095872 27552707

[35] Sawayama H Ogata Y Ishimoto T Mima K Hiyoshi Y Iwatsuki M. Glucose transporter 1 regulates the proliferation and cisplatin sensitivity of esophageal cancer. Cancer Sci (2019) 110(5):1705–14. 10.1111/cas.13995 PMC6500964 30861255

[36] Zhao F Ming J Zhou Y Fan L. Inhibition of Glut1 by WZB117 sensitizes radioresistant breast cancer cells to irradiation. Cancer Chemother Pharmacol (2016) 77(5):963–72. 10.1007/s00280-016-3007-9 27011212

[37] Peng Y Xing SN Tang HY Wang CD Yi FP Liu GL. Influence of glucose transporter 1 activity inhibition on neuroblastoma in vitro. Gene (2019) 689:11–7. 10.1016/j.gene.2018.12.010 30553996

[38] Rae C Sey CHC Mairs RJ. Radiosensitization of Prostate Cancer Cells by 2-Deoxyglucose. Madridge J Oncogenesis (2018) 2(1):30–4. 10.18689/mjo-1000105

[39] Lin X Zhang F Bradbury CM Kaushal A Li L Spitz DR. 2-Deoxy-D-glucose-induced cytotoxicity and radiosensitization in tumor cells is mediated via disruptions in thiol metabolism. Cancer Res (2003) 63(12):3413–7. 12810678

[40] Coleman MC Asbury CR Daniels D Du J Aykin-Burns N Smith BJ. 2-Deoxy-d-glucose causes cytotoxicity, oxidative stress, and radiosensitization in pancreatic cancer. Free Radical Biol Med (2008) 44(3):322–31. 10.1016/j.freeradbiomed.2007.08.032 18215740

[41] Rashmi R Huang X Floberg JM Elhammali AE McCormick ML Patti GJ. Radioresistant cervical cancers are sensitive to inhibition of glycolysis and redox metabolism. Cancer Res (2018) 78(6):1392–403. 10.1158/0008-5472.CAN-17-2367 PMC5856626 29339540

[42] Meijer TWH Peeters WJM Dubois LJ van Gisbergen MW Biemans R Venhuizen JH. Targeting glucose and glutamine metabolism combined with radiation therapy in non-small cell lung cancer. Lung Cancer (2018) 126:32–40. 10.1016/j.lungcan.2018.10.016 30527190

[43] Vanhove K Graulus GJ Mesotten L Thomeer M Derveaux E Noben JP. The Metabolic Landscape of Lung Cancer: New Insights in a Disturbed Glucose Metabolism. Front Oncol (2019) 9:1215. 10.3389/fonc.2019.01215 31803611 PMC6873590

[44] Grasso D Zampieri LX Capeloa T Van de Velde JA Sonveaux P. Mitochondria in cancer. Cell Stress (2020) 4(6):114–46. 10.15698/cst2020.06.221 PMC7278520 32548570

[45] Zachar Z Marecek J Maturo C Gupta S Stuart SD Howell K. Non-redox-active lipoate derivates disrupt cancer cell mitochondrial metabolism and are potent anticancer agents in vivo. J Mol Med (2011) 89(11):1137–48. 10.1007/s00109-011-0785-8 21769686

[46] A Study of CPI-613 With Gemcitabine and Nab-paclitaxel for Patients With Advanced or Metastatic Pancreatic Cancer, Full Text View - ClinicalTrials.gov: NCT03435289.

[47] Gustafson CT Mamo T Shogren KL Maran A Yaszemski MJ. FH535 Suppresses Osteosarcoma Growth In Vitro and Inhibits Wnt Signaling through Tankyrases. Front Pharmacol (2017) 8:285. 10.3389/fphar.2017.00285 28588489 PMC5440578

[48] Turcios L Marti F Watt DS Kril LM Khurana A Chapelin F. Mitochondrial uncoupling and the disruption of the metabolic network in hepatocellular carcinoma. Oncotarget (2020) 11(31):3013–24. 10.18632/oncotarget.27680 PMC7415405 32821346

[49] Dhillon S. Ivosidenib: First Global Approval. Drugs (2018) 78(14):1509–16. 10.1007/s40265-018-0978-3 PMC6315051 30209701

[50] Stein EM DiNardo CD Pollyea DA Fathi AT Roboz GJ Altman JK. Enasidenib in mutant IDH2 relapsed or refractory acute myeloid leukemia. Blood (2017) 130(6):722–31. 10.1182/blood-2017-04-779405 PMC5572791 28588020

[51] Pollyea DA Tallman MS de Botton S Kantarjian HM Collins R Stein AS. Enasidenib, an inhibitor of mutant IDH2 proteins, induces durable remissions in older patients with newly diagnosed acute myeloid leukemia. Leukemia (2019) 33(11):2575–84. 10.1038/s41375-019-0472-2 PMC9724489 30967620

[52] Golub D Iyengar N Dogra S Wong T Bready D Tang K. Mutant Isocitrate Dehydrogenase Inhibitors as Targeted Cancer Therapeutics. Front Oncol (2019) 9:417. 10.3389/fonc.2019.00417 31165048 PMC6534082

[53] Study of Orally Administered AG-881 in Patients With Advanced Solid Tumors, Including Gliomas, With an IDH1 and/or IDH2 Mutation, Full Text View - ClinicalTrials.gov: NCT02481154.

[54] Chaturvedi A Herbst L Pusch S Klett L Goparaju R Stichel D. Pan-mutant-IDH1 inhibitor BAY1436032 is highly effective against human IDH1 mutant acute myeloid leukemia in vivo. Leukemia (2017) 31(10):2020–8. 10.1038/leu.2017.46 PMC5629366 28232670

[55] BAY1436032 in Patients With Mutant IDH1(mIDH1) Advanced Acute Myeloid Leukemia (AML), Full Text View - ClinicalTrials.gov: NCT03127735.

[56] Owen MR Doran E Halestrap AP. Evidence that metformin exerts its anti-diabetic effects through inhibition of complex 1 of the mitochondrial respiratory chain. Biochem J (2000) 348(3):607–14. 10.1042/bj3480607 PMC1221104 10839993

[57] Gulati S Desai J Palackdharry SM Morris JC Zhu Z Jandarov R. Phase 1 dose-finding study of metformin in combination with concurrent cisplatin and radiotherapy in patients with locally advanced head and neck squamous cell cancer. Cancer (2020) 126(2):354–62. 10.1002/cncr.32539 PMC10402880 31626727

[58] Segal ED Yasmeen A Beauchamp MC Rosenblatt J Pollak M Gotlieb WH. Relevance of the OCT1 transporter to the antineoplastic effect of biguanides. Biochem Biophys Res Commun (2011) 414(4):694–9. 10.1016/j.bbrc.2011.09.134 21986525

[59] Clinical Trial of Phenformin in Combination With Dabrafenib and Trametinib for Patients With BRAF-mutated Melanoma, Full Text View - ClinicalTrials.gov: NCT03026517.

[60] Chappie TA Humphrey JM Allen MP Estep KG Fox CB Lebel LA. Discovery of a series of 6,7-dimethoxy-4-pyrrolidylquinazoline PDE10A inhibitors. J Medicinal Chem (2007) 50(2):182–5. 10.1021/jm060653b 17228859

[61] Benej M Hong X Vibhute S Scott S Wu J Graves E. Papaverine and its derivatives radiosensitize solid tumors by inhibiting mitochondrial metabolism. Proc Natl Acad Sci U S A (2018) 115(42):10756–61. 10.1073/pnas.1808945115 PMC6196495 30201710

[62] Ashton TM Fokas E Kunz-Schughart LA Folkes LK Anbalagan S Huether M. The anti-malarial atovaquone increases radiosensitivity by alleviating tumour hypoxia. Nat Commun (2016) 7:12308. 10.1038/ncomms12308 27453292 PMC4962491

[63] Atovaquone as Tumour HypOxia Modifier, Full Text View - ClinicalTrials.gov: NCT02628080.

[64] Buckley AM Dunne MR Lynam-Lennon N Kennedy SA Cannon A Reynolds AL. Pyrazinib (P3), [(E)-2-(2-Pyrazin-2-yl-vinyl)-phenol], a small molecule pyrazine compound enhances radiosensitivity in oesophageal adenocarcinoma. Cancer Lett (2019) 447:115–29. 10.1016/j.canlet.2019.01.009 30664962

[65] Singh PK Deorukhkar AA Venkatesulu BP Li X Tailor R Bomalaski JS. Exploiting arginine auxotrophy with pegylated arginine deiminase (ADI-PEG20) to sensitize pancreatic cancer to radiotherapy via metabolic dysregulation. Mol Cancer Ther (2019) 18(12):2381–93. 10.1158/1535-7163.MCT-18-0708 PMC6891156 31395686

[66] Kremer JC Van Tine BA. Therapeutic arginine starvation in ASS1-deficient cancers inhibits the Warburg effect. Mol Cell Oncol (2017) 4(3):e1295131. 10.1080/23723556.2017.1295131 28616574 PMC5462516

[67] Lowery MA Yu KH Kelsen DP Harding JJ Bomalaski JS Glassman DC. A phase 1/1B trial of ADI-PEG 20 plus nab-paclitaxel and gemcitabine in patients with advanced pancreatic adenocarcinoma. Cancer (2017) 123(23):4556–65. 10.1002/cncr.30897 28832976

[68] Kridel SJ Axelrod F Rozenkrantz N Smith JW. Orlistat Is a Novel Inhibitor of Fatty Acid Synthase with Antitumor Activity. Cancer Res (2004) 64(6):2070–5. 10.1158/0008-5472.CAN-03-3645 15026345

[69] Ge Y Liu J Yang X Zhu H Yang B Zhao K. Fenofibrate enhances radiosensitivity of esophageal squamous cell carcinoma by suppressing hypoxia-inducible factor-1α expression. Tumor Biol (2014) 35(11):10765–71. 10.1007/s13277-014-2149-9 25073512

[70] Li XQ Zhou JD Zou ST Yu J Meng XJ Wu JC. Enhancement of radiosensitivity in human esophageal carcinoma cells by fenofibrate and its potential mechanism. Tumori (2015) 101(1):123–30. 10.5301/tj.5000228 25712601

[71] Chen L Peng J Wang Y Jiang H Wang W Dai J. Fenofibrate-induced mitochondrial dysfunction and metabolic reprogramming reversal: the anti-tumor effects in gastric carcinoma cells mediated by the PPAR pathway. Am J Trans Res (2020) 12(2):428–8. PMC7061836 32194894

[72] Boysen G Jamshidi-Parsian A Davis MA Siegel ER Simecka CM Kore RA. Glutaminase inhibitor CB-839 increases radiation sensitivity of lung tumor cells and human lung tumor xenografts in mice. Int J Radiat Biol (2019) 95(4):436–42. 10.1080/09553002.2018.1558299 PMC6622448 30557074

[73] Gross MI Demo SD Dennison JB Chen L Chernov-Rogan T Goyal B. Antitumor activity of the glutaminase inhibitor CB-839 in triple-negative breast cancer. Mol Cancer Ther (2014) 13(4):890–901. 10.1158/1535-7163.MCT-13-0870 24523301

[74] CB-839 + Capecitabine in Solid Tumors and Fluoropyrimidine Resistant PIK3CA Mutant Colorectal Cancer, Full Text View - ClinicalTrials.gov: NCT02861300.

[75] Glutaminase Inhibitor CB-839 Hydrochloride and Osimertinib in Treating Patients With EGFR-Mutated Stage IV Non-small Cell Lung Cancer, Full Text View - ClinicalTrials.gov: NCT03831932.

[76] Wang H Bouzakoura S de Mey S Jiang H Law K Dufait I. Auranofin radiosensitizes tumor cells through targeting thioredoxin reductase and resulting overproduction of reactive oxygen species. Oncotarget (2017) 8(22):35728–42. 10.18632/oncotarget.16113 PMC5482612 28415723

[77] Pauwels B Korst AEC De Pooter CMJ Lambrechts HAJ Pattyn GGO Lardon F. The radiosensitising effect of gemcitabine and the influence of the rescue agent amifostine in vitro. Eur J Cancer (2003) 39(6):838–46. 10.1016/S0959-8049(03)00002-9 12651211

[78] Simone NL Ménard C Soule BP Albert PS Guion P Smith S. Intrarectal Amifostine During External Beam Radiation Therapy for Prostate Cancer Produces Significant Improvements in Quality of Life Measured by EPIC Score. Int J Radiat Oncol Biol Phys (2008) 70(1):90–5. 10.1016/j.ijrobp.2007.05.057 PMC2267374 17855015

[79] Bardet E Martin L Calais G Alfonsi M Feham NE Tuchais C. Subcutaneous compared with intravenous administration of amifostine in patients with head and neck cancer receiving radiotherapy: Final results of the GORTEC 2000-02 phase III randomized trial. J Clin Oncol (2011) 29(2):127–33. 10.1200/JCO.2009.25.5638 21115863

[80] Peebles DD Soref CM Copp RR Thunberg AL Fahl WE. ROS-Scavenger and Radioprotective Efficacy of the New PrC-210 Aminothiol. Radiat Res (2012) 178(1):57–68. 10.1667/RR2806.1 22702647 PMC3409661

[81] Fahl WE Jermusek F Guerin T Albrecht DM Fahl CJS Dreischmeier E. Impact of the PrC-210 Radioprotector Molecule on Cancer Deaths in p53-Deficient Mice. Radiat Res (2019) 193(1):88–8. 10.1667/RR15439.1 31738662

[82] Epperly MW Gretton JE Sikora CA Jefferson M Bernarding M Nie S. Mitochondrial Localization of Superoxide Dismutase is Required for Decreasing Radiation-Induced Cellular Damage. Radiat Res (2003) 160(5):568–78. 10.1667/RR3081 14565825

[83] Greenberger JS Shinde A Berhane H Dixon T Franicola D Li S. Mitochondrial Localization of GS-Nitroxide JP4-039 Delivered in Intraoral Emulsion Ameliorates Radiation Mucositis in Fanconi Anemia (FA) Fancd2-/- Mice. Int J Radiat Oncol Biol Physics (2015) 93(3):E540–1. 10.1016/j.ijrobp.2015.07.1931

[84] Shinde A Berhane H Rhieu BH Kalash R Xu K Goff J. Intraoral Mitochondrial-Targeted GS-Nitroxide, JP4-039, Radioprotects Normal Tissue in Tumor-Bearing Radiosensitive Fancd2(-/-) (C57BL/6) Mice. Radiat Res (2016) 185(2):134–50. 10.1667/RR14035.1 PMC4773657 26789701

[85] Gandhi KA Goda JS Gandhi VV Sadanpurwala A Jain VK Joshi K. Oral administration of 3,3′-diselenodipropionic acid prevents thoracic radiation induced pneumonitis in mice by suppressing NF-kB/IL-17/G-CSF/neutrophil axis. Free Radical Biol Med (2019) 145:8–19. 10.1016/j.freeradbiomed.2019.09.009 31521664

[86] Gota V Goda JS Doshi K Patil A Sunderajan S Kumar K. Biodistribution and Pharmacokinetic Study of 3,3′ Diseleno Dipropionic Acid (DSePA), A Synthetic Radioprotector. Mice Eur J Drug Metab Pharmacokinet (2016) 41(6):839–44. 10.1007/s13318-015-0301-6 26446594

[87] Araujo-Mino EP Patt YZ Murray-Krezan C Hanson JA Bansal P Liem BJ. Phase II Trial Using a Combination of Oxaliplatin, Capecitabine, and Celecoxib with Concurrent Radiation for Newly Diagnosed Resectable Rectal Cancer. Oncologist (2018) 23(1):2–2. 10.1634/theoncologist.2017-0474 29158365 PMC5759821

[88] Kumar KS Raghavan M Hieber K Ege C Mog S Parra N. Preferential radiation sensitization of prostate cancer in nude mice by nutraceutical antioxidant γ-tocotrienol. Life Sci (2006) 78(18):2099–104. 10.1016/j.lfs.2005.12.005 16413038

[89] Sayed R El Wakeel L Saad AS Kelany M El-Hamamsy M. Pentoxifylline and vitamin E reduce the severity of radiotherapy-induced oral mucositis and dysphagia in head and neck cancer patients: a randomized, controlled study. Med Oncol (2020) 37(1):8–8. 10.1007/s12032-019-1334-5 31748905

[90] Alexander MS Wilkes JG Schroeder SR Buettner GR Wagner BA Du J. Pharmacologic ascorbate reduces radiation-induced normal tissue toxicity and enhances tumor radiosensitization in pancreatic cancer. Cancer Res (2018) 78(24):6838–51. 10.1158/0008-5472.CAN-18-1680 PMC6295907 30254147

[91] Wei X Xu Y Xu FF Chaiswing L Schnell D Noel T. RelB expression determines the differential effects of ascorbic acid in normal and cancer cells. Cancer Res (2017) 77(6):1345–56. 10.1158/0008-5472.CAN-16-0785 PMC5354963 28108513

[92] Singh S Aggarwal BB. Activation of transcription factor NF-κB is suppressed by curcumin (diferulolylmethane). J Biol Chem (1995) 270(42):24995–5000. 10.1074/jbc.270.42.24995 7559628

[93] Fardid R Salajegheh A Mosleh-Shirazi MA Sharifzadeh S Okhovat MA Najafi M. Melatonin ameliorates the production of COX-2, iNOS, and the formation of 8-OHdG in non-targeted lung tissue after pelvic irradiation. Cell J (2017) 19(2):324–31. 10.22074/cellj.2016.3857 PMC5412791 28670525

[94] Farhood B Aliasgharzadeh A Amini P Rezaeyan A Tavassoli A Motevaseli E. Mitigation of radiation-induced lung pneumonitis and fibrosis using metformin and melatonin: A histopathological study. Medicina (Lithuania) (2019) 55(8):417. 10.3390/medicina55080417 PMC6722577 31366142

[95] Onseng K Johns NP Khuayjarernpanishk T Subongkot S Priprem A Hurst C. Beneficial Effects of Adjuvant Melatonin in Minimizing Oral Mucositis Complications in Head and Neck Cancer Patients Receiving Concurrent Chemoradiation. J Altern Complement Med (2017) 23(12):957–63. 10.1089/acm.2017.0081 28657801

[96] Ben-David MA Elkayam R Gelernter I Pfeffer RM. Melatonin for prevention of breast radiation dermatitis: A phase II, prospective, double-blind randomized trial. Israel Med Assoc J (2016) 18(3-4):188–92. 27228641

[97] Berk L Berkey B Rich T Hrushesky W Blask D Gallagher M. Randomized Phase II Trial of High-Dose Melatonin and Radiation Therapy for RPA Class 2 Patients With Brain Metastases (RTOG 0119). Int J Radiat Oncol Biol Phys (2007) 68(3):852–7. 10.1016/j.ijrobp.2007.01.012 PMC2709786 17418968

[98] Yildiz OG Soyuer S Saraymen R Eroglu C. Protective effects of caffeic acid phenethyl ester on radiation induced lung injury in rats. Clin Invest Med (2008) 31(5):E24–7. 10.25011/cim.v31i5.4870 18980713

[99] Chen MF Keng PC Lin PY Yang CT Liao SK Chen WC. Caffeic acid phenethyl ester decreases acute pneumonitis after irradiation in vitro and in vivo. BMC Cancer (2005) 5:158. 10.1186/1471-2407-5-158 16336675 PMC1325253

[100] Song L Turkson J Karras JG Jove R Haura EB. Activation of Stat3 by receptor tyrosine kinases and cytokines regulates survival in human non-small cell carcinoma cells. Oncogene (2003) 22(27):4150–65. 10.1038/sj.onc.1206479 12833138

[101] Vendrely V Amintas S Noel C Moranvillier I Lamrissi I Rousseau B. Combination treatment of resveratrol and capsaicin radiosensitizes pancreatic tumor cells by unbalancing DNA repair response to radiotherapy towards cell death. Cancer Lett (2019) 451:1–10. 10.1016/j.canlet.2019.02.038 30849482

[102] Kim JS Jeong SK Oh SJ Lee CG Kang YR Jo WS. The resveratrol analogue, HS-1793, enhances the effects of radiation therapy through the induction of anti-tumor immunity in mammary tumor growth. Int J Oncol (2020) 56(6):1405–16. 10.3892/ijo.2020.5017 PMC7170036 32236622

[103] Safdie F Brandhorst S Wei M Wang W Lee C Hwang S. Fasting Enhances the Response of Glioma to Chemo- and Radiotherapy. PloS One (2012) 7(9):e44603. 10.1371/journal.pone.0044603 22984531 PMC3439413

[104] de la Cruz Bonilla M Stemler KM Jeter-Jones S Fujimoto TN Molkentine J Asencio Torres GM. Fasting Reduces Intestinal Radiotoxicity, Enabling Dose-Escalated Radiation Therapy for Pancreatic Cancer. Int J Radiat Oncol Biol Phys (2019) 105(3):537–47. 10.1016/j.ijrobp.2019.06.2533 PMC6754784 31271824

[105] Voss M Wagner M von Mettenheim N Harter PN Wenger KJ Franz K. ERGO2: A Prospective, Randomized Trial of Calorie-Restricted Ketogenic Diet and Fasting in Addition to Reirradiation for Malignant Glioma. Int J Radiat Oncol Biol Phys (2020) 108(4):987–95. 10.1016/j.ijrobp.2020.06.021 32619561

[106] Weber DD Aminzadeh-Gohari S Tulipan J Catalano L Feichtinger RG Kofler B. Ketogenic diet in the treatment of cancer - Where do we stand? Mol Metab (2020) 33:102–21. 10.1016/j.molmet.2019.06.026 PMC7056920 31399389

[107] Hanahan D Weinberg RA. Hallmarks of cancer: The next generation. Cell Press (2011)144:646–74. 10.1016/j.cell.2011.02.013 21376230

[108] Schafer ZT Grassian AR Song L Jiang Z Gerhart-Hines Z Irie HY. Antioxidant and oncogene rescue of metabolic defects caused by loss of matrix attachment. Nature (2009) 461(7260):109–13. 10.1038/nature08268 PMC2931797 19693011

[109] Wei Q Qian Y Yu J Wong CC. Metabolic rewiring in the promotion of cancer metastasis: mechanisms and therapeutic implications. Oncogene (2020) 39(39):6139–56. 10.1038/s41388-020-01432-7 PMC7515827 32839493

[110] Pavlova NN Thompson CB. The Emerging Hallmarks of Cancer Metabolism. Cell Press (2016)23(1) 27–47. 10.1016/j.cmet.2015.12.006 PMC4715268 26771115

[111] Yuneva MO Fan TW Allen TD Higashi RM Ferraris DV Tsukamoto T. The metabolic profile of tumors depends on both the responsible genetic lesion and tissue type. Cell Metab (2012) 15(2):157–70. 10.1016/j.cmet.2011.12.015 PMC3282107 22326218

[112] DeBerardinis RJ Mancuso A Daikhin E Nissim I Yudkoff M Wehrli S. Beyond aerobic glycolysis: transformed cells can engage in glutamine metabolism that exceeds the requirement for protein and nucleotide synthesis. Proc Natl Acad Sci USA (2007) 104(49):19345–50. 10.1073/pnas.0709747104 PMC2148292 18032601

[113] Altman BJ Stine ZE Dang CV. From Krebs to clinic: glutamine metabolism to cancer therapy. Nat Rev Cancer (2016) 16(10):619–34. 10.1038/nrc.2016.71 PMC5484415 27492215

[114] Racker E. Bioenergetics and the problem of tumor growth. Am Scientist (1972) 60(1):56–63. 4332766

[115] Liberti MV Locasale JW. The Warburg Effect: How Does it Benefit Cancer Cells? Elsevier Ltd (2016)41(3): 211–8. 10.1016/j.tibs.2015.12.001 PMC4783224 26778478

[116] Warburg O. On the origin of cancer cells. Science (1956) 123(3191):309–14. 10.1126/science.123.3191.309 13298683

[117] Payen VL Porporato PE Baselet B Sonveaux P. Metabolic changes associated with tumor metastasis, part 1: tumor pH, glycolysis and the pentose phosphate pathway. Cell Mol Life Sci (2016) 73(7):1333–48. 10.1007/s00018-015-2098-5 PMC11108399 26626411

[118] Koundouros N Poulogiannis G. Reprogramming of fatty acid metabolism in cancer. Br J Cancer (2020) 122(1):4–22. 10.1038/s41416-019-0650-z 31819192 PMC6964678

[119] Hacker HJ Steinberg P Bannasch P. Pyruvate kinase isoenzyme shift from L-type to M2-type is a late event in hepatocarcinogenesis induced in rats by a choline-deficient/DL-ethionine-supplemented diet. Carcinogenesis (1998) 19(1):99–107. 10.1093/carcin/19.1.99 9472700

[120] Mellati AA Yücel M Altinörs N Gündüz U. Regulation of M2-type pyruvate kinase from human meningioma by allosteric effectors fructose 1,6 diphosphate and L-alanine. Cancer Biochem Biophys (1992) 13(1):33–41. 1343845

[121] Oremek GM Teigelkamp S Kramer W Eigenbrodt E Usadel KH. The pyruvate kinase isoenzyme tumor M2 (Tu M2-PK) as a tumor marker for renal carcinoma. Anticancer Res (1999) 19(4A):2599–601. 10470201

[122] Zu XL Guppy M. Cancer metabolism: Facts, fantasy, and fiction. Academic Press Inc (2004)313(3):459–65. 10.1016/j.bbrc.2003.11.136 14697210

[123] Vazquez A Kamphorst JJ Markert EK Schug ZT Tardito S Gottlieb E. Cancer metabolism at a glance. J Cell Sci (2016) 129(18):3367–73. 10.1242/jcs.181016 PMC6518336 27635066

[124] Yang L Venneti S Nagrath D. Glutaminolysis: A Hallmark of Cancer Metabolism. Annu Rev Biomed Eng (2017) 19(1):163–94. 10.1146/annurev-bioeng-071516-044546 28301735

[125] Röhrig F Schulze A. The multifaceted roles of fatty acid synthesis in cancer. Nature Publishing Group (2016)16(11):732–49. 10.1038/nrc.2016.89 27658529

[126] Bauer DE Hatzivassiliou G Zhao F Andreadis C Thompson CB. ATP citrate lyase is an important component of cell growth and transformation. Oncogene (2005) 24(41):6314–22. 10.1038/sj.onc.1208773 16007201

[127] Teng L Chen Y Cao Y Wang W Xu Y Wang Y. Overexpression of ATP citrate lyase in renal cell carcinoma tissues and its effect on the human renal carcinoma cells in vitro. Oncol Lett (2018) 15(5):6967–74. 10.3892/ol.2018.8211 PMC5920499 29725424

[128] Li Z Liu H Luo X. Lipid droplet and its implication in cancer progression. Am J Cancer Res (2020) 10(12):4112–22. PMC7783747 33414989

[129] Cruz ALS Barreto EA Fazolini NPB Viola JPB Bozza PT. Lipid droplets: platforms with multiple functions in cancer hallmarks. Cell Death Dis (2020) 11(2):105. 10.1038/s41419-020-2297-3 32029741 PMC7005265

[130] Cairns RA Harris IS Mak TW. Regulation of cancer cell metabolism. Nat Rev Cancer (2011) 11(2):85–95. 10.1038/nrc2981 21258394

[131] Porporato PE Payen VL Baselet B Sonveaux P. Metabolic changes associated with tumor metastasis, part 2: Mitochondria, lipid and amino acid metabolism. Cell Mol Life Sci (2016) 73(7):1349–63. 10.1007/s00018-015-2100-2 PMC11108268 26646069

[132] Edwards E Geng L Tan J Onishko H Donnelly E Hallahan DE. Phosphatidylinositol 3-Kinase/Akt Signaling in the Response of Vascular Endothelium to Ionizing Radiation. Cancer Res (2002) 62(16):4671 LP–4677. 12183424

[133] Berwick DC Hers I Heesom KJ Kelly Moule S Tavaré JM. The identification of ATP-citrate lyase as a protein kinase B (Akt) substrate in primary adipocytes. J Biol Chem (2002) 277(37):33895–900. 10.1074/jbc.M204681200 12107176

[134] Xia L Tan S Zhou Y Lin J Wang H Oyang L. Role of the NFκB-signaling pathway in cancer. OncoTargets Ther (2018) 11:2063–73. 10.2147/OTT.S161109 PMC5905465 29695914

[135] Bielski BHJ Cabelli DE Arudi RL Ross AB. Reactivity of HO2/O–2 Radicals in Aqueous Solution. J Phys Chem Reference Data (1985) 14(4):1041–100. 10.1063/1.555739

[136] Shadyro OI Yurkova IL Kisel MA. Radiation-induced peroxidation and fragmentation of lipids in a model membrane. Int J Radiat Biol (2002) 78(3):211–7. 10.1080/09553000110104065 11869476

[137] Dobrzyńska I Szachowicz-Petelska B Skrzydlewska E Figaszewski ZA. Effects of UVB Radiation on the Physicochemical Properties of Fibroblasts and Keratinocytes. J Membrane Biol (2016) 249(3):319–25. 10.1007/s00232-016-9870-9 26809654

[138] Güler G Tomruk A Ozgur E Sahin D Sepici A Altan N. The effect of radiofrequency radiation on DNA and lipid damage in female and male infant rabbits. Int J Radiat Biol (2012) 88(4):367–73. 10.3109/09553002.2012.646349 22145622

[139] Sudprasert W Navasumrit P Ruchirawat M. Effects of low-dose gamma radiation on DNA damage, chromosomal aberration and expression of repair genes in human blood cells. Int J Hygiene Environ Health (2006) 209(6):503–11. 10.1016/j.ijheh.2006.06.004 16872898

[140] Mozdarani H Nasirian B Haeri SA. In vivo γ-rays Induced Initial DNA Damage and the Effect of Famotidine in Mouse Leukocytes as Assayed by the Alkaline Comet Assay. J Radiat Res (2007) 48(2):129–34. 10.1269/jrr.06055 17299251

[141] Ramkumar S Fujii N Fujii N Thankappan B Sakaue H Ingu K. Comparison of effect of gamma ray irradiation on wild-type and N-terminal mutants of αA-crystallin. Mol Vision (2014) 20:1002–16. PMC4087120 25018622

[142] Parshad R Sanford KK Jones GM. Chromatid damage after G2 phase x-irradiation of cells from cancer-prone individuals implicates deficiency in DNA repair. Proc Natl Acad Sci U S A (1983) 80(181):5612–6. 10.1073/pnas.80.18.5612 PMC384308 6577447

[143] Shahidi M Mozdarani H Bryant PE. Radiation sensitivity of leukocytes from healthy individuals and breast cancer patients as measured by the alkaline and neutral comet assay. Cancer Lett (2007) 257(2):263–73. 10.1016/j.canlet.2007.08.002 17881118

[144] Pajic J Rovcanin B. Ionizing radiation-induced genotoxic and oxidative damage in peripheral lymphocytes and plasma of healthy donors. Mutat Research/Genetic Toxicol Environ Mutagenesis (2021) 863–4:503313. 10.1016/j.mrgentox.2021.503313 33678245

[145] Qin L Fan M Candas D Jiang G Papadopoulos S Tian L. CDK1 Enhances Mitochondrial Bioenergetics for Radiation-Induced DNA Repair. Cell Rep (2015) 13(10):2056–63. 10.1016/j.celrep.2015.11.015 PMC4684969 26670043

[146] Tang L Wei F Wu Y He Y Shi L Xiong F. Role of metabolism in cancer cell radioresistance and radiosensitization methods. J Exp Clin Cancer Res (2018) 37(1):87. 10.1186/s13046-018-0758-7 29688867 PMC5914062

[147] Dittmann K Mayer C Rodemann HP Huber SM. EGFR cooperates with glucose transporter SGLT1 to enable chromatin remodeling in response to ionizing radiation. Radiother Oncol (2013) 107(2):247–51. 10.1016/j.radonc.2013.03.016 23602371

[148] Mews P Donahue G Drake AM Luczak V Abel T Berger SL. Acetyl-CoA synthetase regulates histone acetylation and hippocampal memory. Nature (2017) 546(7658):381–6. 10.1038/nature22405 PMC5505514 28562591

[149] Turgeon MO Perry NJS Poulogiannis G Damage DNA. Repair, and Cancer Metabolism. Front Oncol (2018) 8:15. 10.3389/fonc.2018.00015 29459886 PMC5807667

[150] Wang P Yuan D Guo F Chen X Zhu L Zhang H. Chromatin remodeling modulates radiosensitivity of the daughter cells derived from cell population exposed to low- and high-LET irradiation. Oncotarget (2017) 8(32):52823–36. 10.18632/oncotarget.17275 PMC5581073 28881774

[151] Horsman MR Overgaard J. The impact of hypoxia and its modification of the outcome of radiotherapy. J Radiat Res (2016) 57 Suppl 1(Suppl 1):i90–8. 10.1093/jrr/rrw007 PMC4990104 26983987

[152] Lin J Xia L Liang J Han Y Wang H Oyang L. The roles of glucose metabolic reprogramming in chemo- and radio-resistance. BioMed Central Ltd (2019)38(1):1–13. 10.1186/s13046-019-1214-z PMC6533757 31122265

[153] Dhani N Fyles A Hedley D Milosevic M. The clinical significance of hypoxia in human cancers. Semin Nucl Med (2015) 45(2):110–21. 10.1053/j.semnuclmed.2014.11.002 25704384

[154] Gilkes DM Semenza GL Wirtz D. Hypoxia and the extracellular matrix: drivers of tumour metastasis. Nat Rev Cancer (2014) 14(6):430–9. 10.1038/nrc3726 PMC4283800 24827502

[155] Jordan BF Sonveaux P. Targeting tumor perfusion and oxygenation to improve the outcome of anticancer therapy. Front Pharmacol (2012) 3:94. 10.3389/fphar.2012.00094 22661950 PMC3357106

[156] Kozin SV Duda DG Munn LL Jain RK. Neovascularization after irradiation: what is the source of newly formed vessels in recurring tumors? J Natl Cancer Inst (2012) 104(12):899–905. 10.1093/jnci/djs239 22572994 PMC3379722

[157] Goedegebuure RSA de Klerk LK Bass AJ Derks S Thijssen V. Combining Radiotherapy With Anti-angiogenic Therapy and Immunotherapy; A Therapeutic Triad for Cancer? Front Immunol (2018) 9:3107. 10.3389/fimmu.2018.03107 30692993 PMC6339950

[158] Thanik VD Chang CC Lerman OZ Greives MR Le H Warren SM. Cutaneous low-dose radiation increases tissue vascularity through upregulation of angiogenic and vasculogenic pathways. J Vasc Res (2010) 47(6):472–80. 10.1159/000313875 20431296

[159] Rankin EB Giaccia AJ. Hypoxic control of metastasis. Science (2016) 352(6282):175–80. 10.1126/science.aaf4405 PMC4898055 27124451

[160] Yang MH Wu MZ Chiou SH Chen PM Chang SY Liu CJ. Direct regulation of TWIST by HIF-1alpha promotes metastasis. Nat Cell Biol (2008) 10(3):295–305. 10.1038/ncb1691 18297062

[161] Sun S Ning X Zhang Y Lu Y Nie Y Han S. Hypoxia-inducible factor-1alpha induces Twist expression in tubular epithelial cells subjected to hypoxia, leading to epithelial-to-mesenchymal transition. Kidney Int (2009) 75(12):1278–87. 10.1038/ki.2009.62 19279556

[162] Zhang W Shi X Peng Y Wu M Zhang P Xie R. HIF-1alpha Promotes Epithelial-Mesenchymal Transition and Metastasis through Direct Regulation of ZEB1 in Colorectal Cancer. PloS One (2015) 10(6):e0129603. 10.1371/journal.pone.0129603 26057751 PMC4461314

[163] Bery F Figiel S Kouba S Fontaine D Gueguinou M Potier-Cartereau M. Hypoxia Promotes Prostate Cancer Aggressiveness by Upregulating EMT-Activator Zeb1 and SK3 Channel Expression. Int J Mol Sci (2020) 21(13):4786. 10.3390/ijms21134786 PMC7369999 32640738

[164] Tam SY Wu VWC Law HKW. Hypoxia-Induced Epithelial-Mesenchymal Transition in Cancers: HIF-1alpha and Beyond. Front Oncol (2020) 10:486. 10.3389/fonc.2020.00486 32322559 PMC7156534

[165] Semenza GL Jiang BH Leung SW Passantino R Concordat JP Maire P. Hypoxia response elements in the aldolase A, enolase 1, and lactate dehydrogenase a gene promoters contain essential binding sites for hypoxia-inducible factor 1. J Biol Chem (1996) 271(51):32529–37. 10.1074/jbc.271.51.32529 8955077

[166] Zhang H Bosch-Marce M Shimoda LA Yee ST Jin HB Wesley JB. Mitochondrial autophagy is an HIF-1-dependent adaptive metabolic response to hypoxia. J Biol Chem (2008) 283(16):10892–903. 10.1074/jbc.M800102200 PMC2447655 18281291

[167] Kim JW Tchernyshyov I Semenza GL Dang CV. HIF-1-mediated expression of pyruvate dehydrogenase kinase: A metabolic switch required for cellular adaptation to hypoxia. Cell Metab (2006) 3(3):177–85. 10.1016/j.cmet.2006.02.002 16517405

[168] Stegen S Van Gastel N Eelen G Ghesquière B D’Anna F Thienpont B. HIF-1α promotes glutamine-mediated redox homeostasis and glycogen-dependent bioenergetics to support postimplantation bone cell survival. Cell Metab (2016) 23(2):265–79. 10.1016/j.cmet.2016.01.002 PMC7611069 26863487

[169] Carmeliet P Dor Y Herber JM Fukumura D Brusselmans K Dewerchin M. Role of HIF-1α in hypoxiamediated apoptosis, cell proliferation and tumour angiogenesis. Nature (1998) 394(6692):485–90. 10.1038/28867 9697772

[170] Elvidge GP Glenny L Appelhoff RJ Ratcliffe PJ Ragoussis J Gleadle JM. Concordant regulation of gene expression by hypoxia and 2-oxoglutarate-dependent dioxygenase inhibition: The role of HIF-1α, HIF-2α, and other pathways. J Biol Chem (2006) 281(22):15215–26. 10.1074/jbc.M511408200 16565084

[171] Papandreou I Cairns RA Fontana L Lim AL Denko NC. HIF-1 mediates adaptation to hypoxia by actively downregulating mitochondrial oxygen consumption. Cell Metab (2006) 3(3):187–97. 10.1016/j.cmet.2006.01.012 16517406

[172] Qiu MZ Han B Luo HY Zhou ZW Wang ZQ Wang FH. Expressions of hypoxia-inducible factor-1α and hexokinase-II in gastric adenocarcinoma: The impact on prognosis and correlation to clinicopathologic features. Tumor Biol (2011) 32(1):159–66. 10.1007/s13277-010-0109-6 20845004

[173] Cheung EC Ludwig RL Vousden KH. Mitochondrial localization of TIGAR under hypoxia stimulates HK2 and lowers ROS and cell death. Proc Natl Acad Sci U States America (2012) 109(50):20491–6. 10.1073/pnas.1206530109 PMC3528527 23185017

[174] Nakashima R Goto Y Koyasu S Kobayashi M Morinibu A Yoshimura M. UCHL1-HIF-1 axis-mediated antioxidant property of cancer cells as a therapeutic target for radiosensitization. Sci Rep (2017) 7(1):6879–9. 10.1038/s41598-017-06605-1 PMC5537219 28761052

[175] Peña-Rico MA Calvo-Vidal MN Villalonga-Planells R Martínez-Soler F Giménez-Bonafé P Navarro-Sabaté À. TP53 induced glycolysis and apoptosis regulator (TIGAR) knockdown results in radiosensitization of glioma cells. Radiother Oncol (2011) 101(1):132–9. 10.1016/j.radonc.2011.07.002 21864926

[176] Lan F Qin Q Yu H Yue X. Effect of glycolysis inhibition by miR-448 on glioma radiosensitivity. J Neurosurg (2019) p:1–9. 10.3171/2018.12.JNS181798 31003211

[177] Zhao L Vogt PK. Class I PI3K in oncogenic cellular transformation. Oncogene (2008) 27(41):5486–96. 10.1038/onc.2008.244 PMC2757120 18794883

[178] Bjorge JD Chan TO Antczak M Kung HJ Fujita DJ. Activated type I phosphatidylinositol kinase is associated with the epidermal growth factor (EGF) receptor following EGF stimulation. Proc Natl Acad Sci U States America (1990) 87(10):3816–20. 10.1073/pnas.87.10.3816 PMC53994 2160078

[179] Goldkorn T Balaban N Shannon M Matsukuma K. EGF receptor phosphorylation is affected by ionizing radiation. Biochim Biophys Acta - Mol Cell Res (1997) 1358(3):289–99. 10.1016/S0167-4889(97)00063-3 9366260

[180] Balaban N Moni J Shannon M Dang L Murphy E Goldkorn T. The effect of ionizing radiation on signal transduction: Antibodies to EGF receptor sensitize A431 cells to radiation. Biochim Biophys Acta - Mol Cell Res (1996) 1314(1-2):147–56. 10.1016/S0167-4889(96)00068-7 8972728

[181] Chang L Graham PH Hao J Ni J Bucci J Cozzi PJ. PI3K/Akt/mTOR pathway inhibitors enhance radiosensitivity in radioresistant prostate cancer cells through inducing apoptosis, reducing autophagy, suppressing NHEJ and HR repair pathways. Cell Death Dis (2014) 5:e1437. 10.1038/cddis.2014.415 25275598 PMC4237243

[182] Yakovlev VA Barani IJ Rabender CS Black SM Leach JK Graves PR. Tyrosine nitration of IκBα: A novel mechanism for NF-κB activation. Biochemistry (2007) 46(42):11671–83. 10.1021/bi701107z PMC2678910 17910475

[183] Brach MA Hass R Sherman ML Gunji H Weichselbaum R Kufe D. Ionizing radiation induces expression and binding activity of the nuclear factor κB. J Clin Invest (1991) 88(2):691–5. 10.1172/JCI115354 PMC295415 1864978

[184] Kowaliuk J Sarsarshahi S Hlawatsch J Kastsova A Kowaliuk M Krischak A. Translational Aspects of Nuclear Factor-Kappa B and Its Modulation by Thalidomide on Early and Late Radiation Sequelae in Urinary Bladder Dysfunction. Int J Radiat Oncol Biol Phys (2020) 107(2):377–85. 10.1016/j.ijrobp.2020.01.028 32035188

[185] Wang Z Huang Y Zhang J. Molecularly targeting the PI3K-Akt-mTOR pathway can sensitize cancer cells to radiotherapy and chemotherapy. Cell Mol Biol Lett (2014) 19(2):233–42. 10.2478/s11658-014-0191-7 PMC6275747 24728800

[186] Criswell T Leskov K Miyamoto S Luo G Boothman DA. Transcription factors activated in mammalian cells after clinically relevant doses of ionizing radiation. Oncogene (2003) 22(37):5813–27. 10.1038/sj.onc.1206680 12947388

[187] Chen PM Wu TC Wang YC Cheng YW Sheu GT Chen CY. Activation of NF-kappaB by SOD2 promotes the aggressiveness of lung adenocarcinoma by modulating NKX2-1-mediated IKKbeta expression. Carcinogenesis (2013) 34(11):2655–63. 10.1093/carcin/bgt220 23784082

[188] Oberley LW McCormick ML Sierra-Rivera E Kasemset-St Clair D. Manganese superoxide dismutase in normal and transformed human embryonic lung fibroblasts. Free Radic Biol Med (1989) 6(4):379–84. 10.1016/0891-5849(89)90083-X 2540070

[189] Connor KM Hempel N Nelson KK Dabiri G Gamarra A Belarmino J. Manganese superoxide dismutase enhances the invasive and migratory activity of tumor cells. Cancer Res (2007) 67(21):10260–7. 10.1158/0008-5472.CAN-07-1204 17974967

[190] Huang J Li JJ. Cell repopulation, rewiring metabolism, and immune regulation in cancer radiotherapy. Radiat Med Prot (2020) 1(1):24–30. 10.1016/j.radmp.2020.02.001

[191] LeBleu VS O’Connell JT Gonzalez Herrera KN Wikman H Pantel K Haigis MC. PGC-1alpha mediates mitochondrial biogenesis and oxidative phosphorylation in cancer cells to promote metastasis. Nat Cell Biol (2014) 16(10):992–1003, 1-15. 10.1038/ncb3039 25241037 PMC4369153

[192] Grasso D Medeiros HCD Zampieri LX Bol V Danhier P van Gisbergen MW. Fitter Mitochondria Are Associated With Radioresistance in Human Head and Neck SQD9 Cancer Cells. Front Pharmacol (2020) 11:263. 10.3389/fphar.2020.00263 32231567 PMC7082361

[193] Zonneveld MI Keulers TGH Rouschop KMA. Extracellular Vesicles as Transmitters of Hypoxia Tolerance in Solid Cancers. Cancers (Basel) (2019) 11(2):154. 10.3390/cancers11020154 PMC6406242 30699970

[194] Krisnawan VE Stanley JA Schwarz JK DeNardo DG. Tumor Microenvironment as a Regulator of Radiation Therapy: New Insights into Stromal-Mediated Radioresistance. Cancers (Basel) (2020) 12(10):2916. 10.3390/cancers12102916 PMC7600316 33050580

[195] Castells M Thibault B Delord JP Couderc B. Implication of tumor microenvironment in chemoresistance: tumor-associated stromal cells protect tumor cells from cell death. Int J Mol Sci (2012) 13(8):9545–71. 10.3390/ijms13089545 PMC3431813 22949815

[196] Whitaker-Menezes D Martinez-Outschoorn UE Lin Z Ertel A Flomenberg N Witkiewicz AK. Evidence for a stromal-epithelial “lactate shuttle” in human tumors: MCT4 is a marker of oxidative stress in cancer-associated fibroblasts. Cell Cycle (2011) 10(11):1772–83. 10.4161/cc.10.11.15659 PMC3142461 21558814

[197] Migneco G Whitaker-Menezes D Chiavarina B Castello-Cros R Pavlides S Pestell RG. Glycolytic cancer associated fibroblasts promote breast cancer tumor growth, without a measurable increase in angiogenesis: evidence for stromal-epithelial metabolic coupling. Cell Cycle (2010) 9(12):2412–22. 10.4161/cc.9.12.11989 20562527

[198] Alcaraz J Carrasco JL Millares L Luis IC Fernandez-Porras FJ Martinez-Romero A. Stromal markers of activated tumor associated fibroblasts predict poor survival and are associated with necrosis in non-small cell lung cancer. Lung Cancer (2019) 135:151–60. 10.1016/j.lungcan.2019.07.020 31446988

[199] Ni WD Yang ZT Cui CA Cui Y Fang LY Xuan YH. Tenascin-C is a potential cancer-associated fibroblasts marker and predicts poor prognosis in prostate cancer. Biochem Biophys Res Commun (2017) 486(3):607–12. 10.1016/j.bbrc.2017.03.021 28341124

[200] Ohuchida K Mizumoto K Murakami M Qian LW Sato N Nagai E. Radiation to stromal fibroblasts increases invasiveness of pancreatic cancer cells through tumor-stromal interactions. Cancer Res (2004) 64(9):3215–22. 10.1158/0008-5472.CAN-03-2464 15126362

[201] Cavaco A Rezaei M Niland S Eble JA. Collateral Damage Intended-Cancer-Associated Fibroblasts and Vasculature Are Potential Targets in Cancer Therapy. Int J Mol Sci (2017) 18(11):2355. 10.3390/ijms18112355 PMC5713324 29112161

[202] Mantoni TS Lunardi S Al-Assar O Masamune A Brunner TB. Pancreatic stellate cells radioprotect pancreatic cancer cells through beta1-integrin signaling. Cancer Res (2011) 71(10):3453–8. 10.1158/0008-5472.CAN-10-1633 PMC3097171 21558392

[203] Attieh Y Clark AG Grass C Richon S Pocard M Mariani P. Cancer-associated fibroblasts lead tumor invasion through integrin-beta3-dependent fibronectin assembly. J Cell Biol (2017) 216(11):3509–20. 10.1083/jcb.201702033 PMC5674886 28931556

[204] MacIver NJ Michalek RD Rathmell JC. Metabolic regulation of T lymphocytes. Annu Rev Immunol (2013) 31:259–83. 10.1146/annurev-immunol-032712-095956 PMC3606674 23298210

[205] van der Windt GJ Pearce EL. Metabolic switching and fuel choice during T-cell differentiation and memory development. Immunol Rev (2012) 249(1):27–42. 10.1111/j.1600-065X.2012.01150.x 22889213 PMC3645891

[206] Geeraerts X Bolli E Fendt SM Van Ginderachter JA. Macrophage Metabolism As Therapeutic Target for Cancer, Atherosclerosis, and Obesity. Front Immunol (2017) 8:289. 10.3389/fimmu.2017.00289 28360914 PMC5350105

[207] Michalek RD Gerriets VA Jacobs SR Macintyre AN MacIver NJ Mason EF. Cutting edge: distinct glycolytic and lipid oxidative metabolic programs are essential for effector and regulatory CD4+ T cell subsets. J Immunol (2011) 186(6):3299–303. 10.4049/jimmunol.1003613 PMC3198034 21317389

[208] Klysz D Tai X Robert PA Craveiro M Cretenet G Oburoglu L. Glutamine-dependent alpha-ketoglutarate production regulates the balance between T helper 1 cell and regulatory T cell generation. Sci Signal (2015) 8(396):ra97. 10.1126/scisignal.aab2610 26420908

[209] Kouidhi S Elgaaied AB Chouaib S. Impact of Metabolism on T-Cell Differentiation and Function and Cross Talk with Tumor Microenvironment. Front Immunol (2017) 8:270. 10.3389/fimmu.2017.00270 28348562 PMC5346542

[210] Isla Larrain MT Rabassa ME Lacunza E Barbera A Creton A Segal-Eiras A. IDO is highly expressed in breast cancer and breast cancer-derived circulating microvesicles and associated to aggressive types of tumors by in silico analysis. Tumour Biol (2014) 35(7):6511–9. 10.1007/s13277-014-1859-3 24687552

[211] Weinlich G Murr C Richardsen L Winkler C Fuchs D. Decreased serum tryptophan concentration predicts poor prognosis in malignant melanoma patients. Dermatology (2007) 214(1):8–14. 10.1159/000096906 17191041

[212] Okamoto A Nikaido T Ochiai K Takakura S Saito M Aoki Y. Indoleamine 2,3-dioxygenase serves as a marker of poor prognosis in gene expression profiles of serous ovarian cancer cells. Clin Cancer Res (2005) 11(16):6030–9. 10.1158/1078-0432.CCR-04-2671 16115948

[213] Brandacher G Perathoner A Ladurner R Schneeberger S Obrist P Winkler C. Prognostic value of indoleamine 2,3-dioxygenase expression in colorectal cancer: effect on tumor-infiltrating T cells. Clin Cancer Res (2006) 12(4):1144–51. 10.1158/1078-0432.CCR-05-1966 16489067

[214] Holmgaard RB Zamarin D Li Y Gasmi B Munn DH Allison JP. Tumor-Expressed IDO Recruits and Activates MDSCs in a Treg-Dependent Manner. Cell Rep (2015) 13(2):412–24. 10.1016/j.celrep.2015.08.077 PMC5013825 26411680

[215] Peng YP Zhang JJ Liang WB Tu M Lu ZP Wei JS. Elevation of MMP-9 and IDO induced by pancreatic cancer cells mediates natural killer cell dysfunction. BMC Cancer (2014) 14:738. 10.1186/1471-2407-14-738 25274283 PMC4287420

[216] Mellor AL Sivakumar J Chandler P Smith K Molina H Mao D. Prevention of T cell-driven complement activation and inflammation by tryptophan catabolism during pregnancy. Nat Immunol (2001) 2(1):64–8. 10.1038/83183 11135580

[217] Bottcher M Renner K Berger R Mentz K Thomas S Cardenas-Conejo ZE. D-2-hydroxyglutarate interferes with HIF-1alpha stability skewing T-cell metabolism towards oxidative phosphorylation and impairing Th17 polarization. Oncoimmunology (2018) 7(7):e1445454. 10.1080/2162402X.2018.1445454 29900057 PMC5993507

[218] Kachikwu EL Iwamoto KS Liao YP DeMarco JJ Agazaryan N Economou JS. Radiation enhances regulatory T cell representation. Int J Radiat Oncol Biol Phys (2011) 81(4):1128–35. 10.1016/j.ijrobp.2010.09.034 PMC3117954 21093169

[219] Qu Y Jin S Zhang A Zhang B Shi X Wang J. Gamma-ray resistance of regulatory CD4+CD25+Foxp3+ T cells in mice. Radiat Res (2010) 173(2):148–57. 10.1667/RR0978.1 20095846

[220] Heylmann D Ponath V Kindler T Kaina B. Comparison of DNA repair and radiosensitivity of different blood cell populations. Sci Rep (2021) 11(1):2478. 10.1038/s41598-021-81058-1 33510180 PMC7843614

[221] Heylmann D Rodel F Kindler T Kaina B. Radiation sensitivity of human and murine peripheral blood lymphocytes, stem and progenitor cells. Biochim Biophys Acta (2014) 1846(1):121–9. 10.1016/j.bbcan.2014.04.009 24797212

[222] Srivastava MK Sinha P Clements VK Rodriguez P Ostrand-Rosenberg S. Myeloid-derived suppressor cells inhibit T-cell activation by depleting cystine and cysteine. Cancer Res (2010) 70(1):68–77. 10.1158/0008-5472.CAN-09-2587 20028852 PMC2805057

[223] Geiger R Rieckmann JC Wolf T Basso C Feng Y Fuhrer T. L-Arginine Modulates T Cell Metabolism and Enhances Survival and Anti-tumor Activity. Cell (2016) 167(3):829–842 e13. 10.1016/j.cell.2016.09.031 27745970 PMC5075284

[224] Vasquez-Dunddel D Pan F Zeng Q Gorbounov M Albesiano E Fu J. STAT3 regulates arginase-I in myeloid-derived suppressor cells from cancer patients. J Clin Invest (2013) 123(4):1580–9. 10.1172/JCI60083 PMC3613901 23454751

[225] Blair TC Bambina S Alice AF Kramer GF Medler TR Baird JR. Dendritic Cell Maturation Defines Immunological Responsiveness of Tumors to Radiation Therapy. J Immunol (2020) 204(12):3416–24. 10.4049/jimmunol.2000194 PMC7571541 32341058

[226] Roberts EW Broz ML Binnewies M Headley MB Nelson AE Wolf DM. Critical Role for CD103(+)/CD141(+) Dendritic Cells Bearing CCR7 for Tumor Antigen Trafficking and Priming of T Cell Immunity in Melanoma. Cancer Cell (2016) 30(2):324–36. 10.1016/j.ccell.2016.06.003 PMC5374862 27424807

[227] Lugade AA Moran JP Gerber SA Rose RC Frelinger JG Lord EM. Local radiation therapy of B16 melanoma tumors increases the generation of tumor antigen-specific effector cells that traffic to the tumor. J Immunol (2005) 174(12):7516–23. 10.4049/jimmunol.174.12.7516 15944250

[228] Gupta A Probst HC Vuong V Landshammer A Muth S Yagita H. Radiotherapy promotes tumor-specific effector CD8+ T cells via dendritic cell activation. J Immunol (2012) 189(2):558–66. 10.4049/jimmunol.1200563 22685313

[229] Herber DL Cao W Nefedova Y Novitskiy SV Nagaraj S Tyurin VA. Lipid accumulation and dendritic cell dysfunction in cancer. Nat Med (2010) 16(8):880–6. 10.1038/nm.2172 PMC2917488 20622859

[230] Gao F Liu C Guo J Sun W Xian L Bai D. Radiation-driven lipid accumulation and dendritic cell dysfunction in cancer. Sci Rep (2015) 5:9613. 10.1038/srep09613 25923834 PMC4413852

[231] de Goede KE Driessen AJM Van den Bossche J. Metabolic Cancer-Macrophage Crosstalk in the Tumor Microenvironment. Biol (Basel) (2020) 9(11):380. 10.3390/biology9110380 PMC7694986 33171762

[232] Brand A Singer K Koehl GE Kolitzus M Schoenhammer G Thiel A. LDHA-Associated Lactic Acid Production Blunts Tumor Immunosurveillance by T and NK Cells. Cell Metab (2016) 24(5):657–71. 10.1016/j.cmet.2016.08.011 27641098

[233] Caronni N Simoncello F Stafetta F Guarnaccia C Ruiz-Moreno JS Opitz B. Downregulation of Membrane Trafficking Proteins and Lactate Conditioning Determine Loss of Dendritic Cell Function in Lung Cancer. Cancer Res (2018) 78(7):1685–99. 10.1158/0008-5472.CAN-17-1307 29363545

[234] Raychaudhuri D Bhattacharya R Sinha BP Liu CSC Ghosh AR Rahaman O. Lactate Induces Pro-tumor Reprogramming in Intratumoral Plasmacytoid Dendritic Cells. Front Immunol (2019) 10:1878. 10.3389/fimmu.2019.01878 31440253 PMC6692712

[235] Husain Z Seth P Sukhatme VP. Tumor-derived lactate and myeloid-derived suppressor cells: Linking metabolism to cancer immunology. Oncoimmunology (2013) 2(11):e26383. 10.4161/onci.26383 24404426 PMC3881600

[236] Leca J Fortin J Mak TW. Illuminating the cross-talk between tumor metabolism and immunity in IDH-mutated cancers. Curr Opin Biotechnol (2020) 68:181–5. 10.1016/j.copbio.2020.11.013 33360716

[237] Wu JY Huang TW Hsieh YT Wang YF Yen CC Lee GL. Cancer-Derived Succinate Promotes Macrophage Polarization and Cancer Metastasis via Succinate Receptor. Mol Cell (2020) 77(2):213–27.e5. 10.1016/j.molcel.2019.10.023 31735641

[238] Kohanbash G Carrera DA Shrivastav S Ahn BJ Jahan N Mazor T. Isocitrate dehydrogenase mutations suppress STAT1 and CD8+ T cell accumulation in gliomas. J Clin Invest (2017) 127(4):1425–37. 10.1172/JCI90644 PMC5373859 28319047

[239] Berghoff AS Kiesel B Widhalm G Wilhelm D Rajky O Kurscheid S. Correlation of immune phenotype with IDH mutation in diffuse glioma. Neuro Oncol (2017) 19(11):1460–8. 10.1093/neuonc/nox054 PMC5737620 28531337

[240] Tyrakis PA Palazon A Macias D Lee KL Phan AT Velica P. S-2-hydroxyglutarate regulates CD8(+) T-lymphocyte fate. Nature (2016) 540(7632):236–41. 10.1038/nature20165 PMC5149074 27798602

[241] Hensley CT Faubert B Yuan Q Lev-Cohain N Jin E Kim J. Metabolic Heterogeneity in Human Lung Tumors. Cell (2016) 164(4):681–94. 10.1016/j.cell.2015.12.034 PMC4752889 26853473

[242] Liu G Luo Q Li H Liu Q Ju Y Song G. Increased Oxidative Phosphorylation Is Required for Stemness Maintenance in Liver Cancer Stem Cells from Hepatocellular Carcinoma Cell Line HCCLM3 Cells. Int J Mol Sci (2020) 21(15):5276. 10.3390/ijms21155276 PMC7432880 32722385

[243] Jones RA Robinson TJ Liu JC Shrestha M Voisin V Ju Y. RB1 deficiency in triple-negative breast cancer induces mitochondrial protein translation. J Clin Invest (2016) 126(10):3739–57. 10.1172/JCI81568 PMC5096803 27571409

[244] Goidts V Bageritz J Puccio L Nakata S Zapatka M Barbus S. RNAi screening in glioma stem-like cells identifies PFKFB4 as a key molecule important for cancer cell survival. Oncogene (2012) 31(27):3235–43. 10.1038/onc.2011.490 22056879

[245] Wang Y Liu Y Malek SN Zheng P Liu Y. Targeting HIF1alpha eliminates cancer stem cells in hematological malignancies. Cell Stem Cell (2011) 8(4):399–411. 10.1016/j.stem.2011.02.006 21474104 PMC3084595

[246] Robey IF Lien AD Welsh SJ Baggett BK Gillies RJ. Hypoxia-inducible factor-1alpha and the glycolytic phenotype in tumors. Neoplasia (2005) 7(4):324–30. 10.1593/neo.04430 PMC1501147 15967109

[247] Sun X Wang M Wang M Yu X Guo J Sun T. Metabolic Reprogramming in Triple-Negative Breast Cancer. Front Oncol (2020) 10:428. 10.3389/fonc.2020.00428 32296646 PMC7136496

[248] Vlashi E Lagadec C Vergnes L Matsutani T Masui K Poulou M. Metabolic state of glioma stem cells and nontumorigenic cells. Proc Natl Acad Sci USA (2011) 108(38):16062–7. 10.1073/pnas.1106704108 PMC3179043 21900605

[249] Bhola NE Jansen VM Koch JP Li H Formisano L Williams JA. Treatment of Triple-Negative Breast Cancer with TORC1/2 Inhibitors Sustains a Drug-Resistant and Notch-Dependent Cancer Stem Cell Population. Cancer Res (2016) 76(2):440–52. 10.1158/0008-5472.CAN-15-1640-T PMC4715956 26676751

[250] Lee KM Giltnane JM Balko JM Schwarz LJ Guerrero-Zotano AL Hutchinson KE. MYC and MCL1 Cooperatively Promote Chemotherapy-Resistant Breast Cancer Stem Cells via Regulation of Mitochondrial Oxidative Phosphorylation. Cell Metab (2017) 26(4):633–47.e7. 10.1158/1538-7445.AM2016-3328 28978427 PMC5650077

[251] Sharanek A Burban A Laaper M Heckel E Joyal JS Soleimani VD. OSMR controls glioma stem cell respiration and confers resistance of glioblastoma to ionizing radiation. Nat Commun (2020) 11(1):4116. 10.1038/s41467-020-17885-z 32807793 PMC7431428

[252] Lee P Malik D Perkons N Huangyang P Khare S Rhoades S. Targeting glutamine metabolism slows soft tissue sarcoma growth. Nat Commun (2020) 11(1):498. 10.1038/s41467-020-14374-1 31980651 PMC6981153

[253] Han S Wei R Zhang X Jiang N Fan M Huang JH. CPT1A/2-Mediated FAO Enhancement-A Metabolic Target in Radioresistant Breast Cancer. Front Oncol (2019) 9:1201. 10.3389/fonc.2019.01201 31803610 PMC6873486

[254] Janiszewska M Suva ML Riggi N Houtkooper RH Auwerx J Clement-Schatlo V. Imp2 controls oxidative phosphorylation and is crucial for preserving glioblastoma cancer stem cells. Genes Dev (2012) 26(17):1926–44. 10.1101/gad.188292.112 PMC3435496 22899010

[255] Kim MS Lee EJ Kim JW Chung US Koh WG Keum KC. Gold nanoparticles enhance anti-tumor effect of radiotherapy to hypoxic tumor. Radiat Oncol J (2016) 34(3):230–8. 10.3857/roj.2016.01788 PMC5066449 27730800

[256] Souers AJ Leverson JD Boghaert ER Ackler SL Catron ND Chen J. ABT-199, a potent and selective BCL-2 inhibitor, achieves antitumor activity while sparing platelets. Nat Med (2013) 19(2):202–8. 10.1038/nm.3048 23291630

[257] Roh TH Yim H Roh J Lee KB Park SH Jeong SY. The loss of succinate dehydrogenase B expression is frequently identified in hemangioblastoma of the central nervous system. Sci Rep (2019) 9(1):5873. 10.1038/s41598-019-42338-z 30971719 PMC6458311

[258] Zhao S Lin Y Xu W Jiang W Zha Z Wang P. Glioma-derived mutations in IDH1 dominantly inhibit IDH1 catalytic activity and induce HIF-1alpha. Science (2009) 324(5924):261–5. 10.1126/science.1170944 PMC3251015 19359588

[259] Sudarshan S Sourbier C Kong HS Block K Valera Romero VA Yang Y. Fumarate hydratase deficiency in renal cancer induces glycolytic addiction and hypoxia-inducible transcription factor 1alpha stabilization by glucose-dependent generation of reactive oxygen species. Mol Cell Biol (2009) 29(15):4080–90. 10.1128/MCB.00483-09 PMC2715796 19470762

[260] Gonçalves E Sciacovelli M Costa ASH Tran MGB Johnson TI Machado D. Post-translational regulation of metabolism in fumarate hydratase deficient cancer cells. Metab Eng (2018) 45:149–57. 10.1016/j.ymben.2017.11.011 PMC5805855 29191787

[261] Selak MA Armour SM MacKenzie ED Boulahbel H Watson DG Mansfield KD. Succinate links TCA cycle dysfunction to oncogenesis by inhibiting HIF-α prolyl hydroxylase. Cancer Cell (2005) 7(1):77–85. 10.1016/j.ccr.2004.11.022 15652751

[262] Isaacs JS Yun JJ Mole DR Lee S Torres-Cabala C Chung YL. HIF overexpression correlates with biallelic loss of fumarate hydratase in renal cancer: Novel role of fumarate in regulation of HIF stability. Cancer Cell (2005) 8(2):143–53. 10.1016/j.ccr.2005.06.017 16098467

[263] Yi T Li H Wang X Wu Z. Enhancement radiosensitization of breast cancer cells by deguelin. Cancer Biother Radiopharm (2008) 23(3):355–62. 10.1089/cbr.2007.0452 18593368

[264] Kang W Zheng X Wang P Guo S. Deguelin exerts anticancer activity of human gastric cancer MGC-803 and MKN-45 cells in vitro. Int J Mol Med (2018) 41(6):3157–66. 10.3892/ijmm.2018.3532 PMC5881843 29512685

[265] Gao F Yu X Li M Zhou L Liu W Li W. Deguelin suppresses non-small cell lung cancer by inhibiting EGFR signaling and promoting GSK3β/FBW7-mediated Mcl-1 destabilization. Cell Death Dis (2020) 11(2):14. 10.1038/s41419-020-2344-0 32081857 PMC7035355

[266] Peng XH Karna P O’Regan RM Liu XJ Naithani R Moriarty RM. Down-regulation of inhibitor of apoptosis proteins by deguelin selectively induces apoptosis in breast cancer cells. Mol Pharmacol (2007) 71(1):101–11. 10.1124/mol.106.027367 17035597

[267] Caboni P Sherer TB Zhang N Taylor G Na HM Greenamyre JT. Rotenone, deguelin, their metabolites, and the rat model of Parkinson’s disease. Chem Res Toxicol (2004) 17(11):1540–8. 10.1021/tx049867r 15540952

[268] Wells SA, Jr Robinson BG Gagel RF Dralle H Fagin JA Santoro M. Vandetanib in patients with locally advanced or metastatic medullary thyroid cancer: a randomized, double-blind phase III trial. J Clin Oncol (2012) 30(2):134–41. 10.1200/JCO.2011.35.5040 PMC3675689 22025146

[269] Wang J Kang M Wen Q Qin YT Wei ZX Xiao JJ. Berberine sensitizes nasopharyngeal carcinoma cells to radiation through inhibition of Sp1 and EMT. Oncol Rep (2017) 37(4):2425–32. 10.3892/or.2017.5499 28350122

[270] Wang J Liu Q Yang Q. Radiosensitization effects of berberine on human breast cancer cells. Int J Mol Med (2012) 30(5):1166–72. 10.3892/ijmm.2012.1095 22895634

[271] Xiao M Yang H Xu W Ma S Lin H Zhu H. Inhibition of α-KG-dependent histone and DNA demethylases by fumarate and succinate that are accumulated in mutations of FH and SDH tumor suppressors. Genes Dev (2012) 26(12):1326–38. 10.1101/gad.191056.112 PMC3387660 22677546

[272] Qiao B Zhang Z Li Y. Association of MGMT promoter methylation with tumorigenesis features in patients with ovarian cancer: A systematic meta-analysis. Mol Genet Genomic Med (2018) 6(1):69–76. 10.1002/mgg3.349 29195029 PMC5823672

[273] Van Den Bent MJ Dubbink HJ Sanson M Van Der Lee-Haarloo CR Hegi M Jeuken JWM. MGMT promoter methylation is prognostic but not predictive for outcome to adjuvant PCV chemotherapy in anaplastic oligodendroglial tumors: A report from EORTC brain tumor group study 26951. J Clin Oncol (2009) 27(35):5881–6. 10.1200/JCO.2009.24.1034 PMC2793037 19901104

[274] Wick W Hartmann C Engel C Stoffels M Felsberg J Stockhammer F. NOA-04 randomized phase III trial of sequential radiochemotherapy of anaplastic glioma with procarbazine, lomustine, and vincristine or temozolomide. J Clin Oncol Off J Am Soc Clin Oncol (2009) 27(35):5874–80. 10.1200/JCO.2009.23.6497 19901110

[275] Wang J Ye C Chen C Xiong H Xie B Zhou J. Glucose transporter GLUT1 expression and clinical outcome in solid tumors: A systematic review and meta-analysis. Oncotarget (2017) 8(10):16875–86. 10.18632/oncotarget.15171 PMC5370007 28187435

[276] Younes M Brown RW Mody DR Fernandez L Laucirica R. GLUT1 expression in human breast carcinoma: Correlation with known prognostic markers. Anticancer Res (1995) 15(6 B):2895–8. 8669885

[277] Amann T Maegdefrau U Hartmann A Agaimy A Marienhagen J Weiss TS. GLUT1 expression is increased in hepatocellular carcinoma and promotes tumorigenesis. Am J Pathol (2009) 174(4):1544–52. 10.2353/ajpath.2009.080596 PMC2671384 19286567

[278] Singh S Pandey S Chawla AS Bhatt AN Roy BG Saluja D. Dietary 2-deoxy-D-glucose impairs tumour growth and metastasis by inhibiting angiogenesis. Eur J Cancer (2019) 123:11–24. 10.1016/j.ejca.2019.09.005 31670076

[279] Oladghaffari M Shabestani Monfared A Farajollahi A Baradaran B Mohammadi M Shanehbandi D. MLN4924 and 2DG combined treatment enhances the efficiency of radiotherapy in breast cancer cells. Int J Radiat Biol (2017) 93(6):590–9. 10.1080/09553002.2017.1294272 28291374

[280] Dearling JLJ Qureshi U Begent RHJ Pedley RB. Combining radioimmunotherapy with antihypoxia therapy 2-deoxy-D-glucose results in reduction of therapeutic efficacy. Clin Cancer Res (2007) 13(6):1903–10. 10.1158/1078-0432.CCR-06-2094 17363547

[281] Priebe W Zielinski R Fokt I Felix E Radjendirane V Arumugam J. Design and Evaluation of Wp1122, an Inhibitor of Glycolysis with Increased Cns Uptake. Neuro-Oncology (2018) 20:86–6. 10.1093/neuonc/noy148.356

[282] Nath K Guo L Nancolas B Nelson DS Shestov AA Lee SC. Mechanism of antineoplastic activity of lonidamine. Biochim Biophys Acta (2016) 1866(2):151–62. 10.1016/j.bbcan.2016.08.001 PMC5138080 27497601

[283] Magno L Terraneo F Bertoni F Tordiglione M Bardelli D Rosignoli MT. Double-blind randomized study of lonidamine and radiotherapy in head and neck cancer. Int J Radiat Oncol Biol Phys (1994) 29(1):45–55. 10.1016/0360-3016(94)90225-9 8175445

[284] Scarantino CW McCunniff AJ Evans G Young CW Paggiarino DA. A prospective randomized comparison of radiation therapy plus lonidamine versus radiation therapy plus placebo as initial treatment of clinically localized but nonresectable nonsmall cell lung cancer. Int J Radiat Oncol Biol Phys (1994) 29(5):999–1004. 10.1016/0360-3016(94)90394-8 8083102

[285] Feinberg T Herbig J Kohl I Las G Cancilla JC Torrecilla JS. Cancer metabolism: the volatile signature of glycolysis-in vitro model in lung cancer cells. J Breath Res (2017) 11(1):016008. 10.1088/1752-7163/aa51d6 28068289

[286] Porporato PE Filigheddu N Pedro JMB Kroemer G Galluzzi L. Mitochondrial metabolism and cancer. Cell Res (2018) 28(3):265–80. 10.1038/cr.2017.155 PMC5835768 29219147

[287] Stuart SD Schauble A Gupta S Kennedy AD Keppler BR Bingham PM. A strategically designed small molecule attacks alpha-ketoglutarate dehydrogenase in tumor cells through a redox process. Cancer Metab (2014) 2(1):4–4. 10.1186/2049-3002-2-4 24612826 PMC4108059

[288] Gao L Xu Z Huang Z Tang Y Yang D Huang J. CPI-613 rewires lipid metabolism to enhance pancreatic cancer apoptosis via the AMPK-ACC signaling. J Exp Clin Cancer Res (2020) 39:73. 10.1186/s13046-020-01579-x 32345326 PMC7187515

[289] Mordhorst BR Kerns KC Schauflinger M Zigo M Murphy SL Ross RM. Pharmacologic treatment with CPI-613 and PS48 decreases mitochondrial membrane potential and increases quantity of autolysosomes in porcine fibroblasts. Sci Rep (2019) 9(1):1–11. 10.1038/s41598-019-45850-4 31263141 PMC6603033

[290] Alistar A Morris BB Desnoyer R Klepin HD Hosseinzadeh K Clark C. Safety and tolerability of the first-in-class agent CPI-613 in combination with modified FOLFIRINOX in patients with metastatic pancreatic cancer: a single-centre, open-label, dose-escalation, phase 1 trial. Lancet Oncol (2017) 18(6):770–8. 10.1016/S1470-2045(17)30314-5 PMC5635818 28495639

[291] Philip PA Buyse ME Alistar AT Lima CM Luther S Pardee TS. A phase III open-label trial to evaluate efficacy and safety of CPI-613 plus modified FOLFIRINOX (mFFX) versus FOLFIRINOX (FFX) in patients with metastatic adenocarcinoma of the pancreas. Future Oncol (2019) 15(28):3189–96. 10.2217/fon-2019-0209 PMC6854438 31512497

[292] Pratheeshkumar P Divya SP Parvathareddy SK Alhoshani NM Al-Badawi IA Tulbah A. FoxM1 and beta-catenin predicts aggressiveness in Middle Eastern ovarian cancer and their co-targeting impairs the growth of ovarian cancer cells. Oncotarget (2018) 9(3):3590–604. 10.18632/oncotarget.23338 PMC5790485 29423068

[293] Su H Jin X Zhang X Zhao L Lin B Li L. FH535 increases the radiosensitivity and reverses epithelial-to-mesenchymal transition of radioresistant esophageal cancer cell line KYSE-150R. J Transl Med (2015) 13:104. 10.1186/s12967-015-0464-6 25888911 PMC4384308

[294] Turcios L Chacon E Garcia C Eman P Cornea V Jiang J. Autophagic flux modulation by Wnt/beta-catenin pathway inhibition in hepatocellular carcinoma. PloS One (2019) 14(2):e0212538. 10.1371/journal.pone.0212538 30794613 PMC6386480

[295] Iida J Dorchak J Lehman JR Clancy R Luo C Chen Y. FH535 inhibited migration and growth of breast cancer cells. PloS One (2012) 7(9):e44418. 10.1371/journal.pone.0044418 22984505 PMC3439405

[296] Patel JP Gönen M Figueroa ME Fernandez H Sun Z Racevskis J. Prognostic relevance of integrated genetic profiling in acute myeloid leukemia. New Engl J Med (2012) 366(12):1079–89. 10.1056/NEJMoa1112304 PMC3545649 22417203

[297] Parsons DW Jones S Zhang X Lin JCH Leary RJ Angenendt P. An integrated genomic analysis of human glioblastoma multiforme. Science (2008) 321(5897):1807–12. 10.1126/science.1164382 PMC2820389 18772396

[298] Yin N Xie T Zhang H Chen J Yu J Liu F. IDH1−R132H mutation radiosensitizes U87MG glioma cells via epigenetic downregulation of TIGAR. Oncol Lett (2020) 19(2):1322–30. 10.3892/ol.2019.11148 PMC6956398 31966064

[299] Trachootham D Lu W Ogasawara MA Valle NRD Huang P. IRedox regulation of cell survival. Antioxid Redox Signal (2008) 10(8):1343–74. 10.1089/ars.2007.1957 PMC2932530 18522489

[300] Zhang H Gu C Yu J Wang Z Yuan X Yang L. Radiosensitization of glioma cells by TP53-induced glycolysis and apoptosis regulator knockdown is dependent on thioredoxin-1 nuclear translocation. Free Radical Biol Med (2014) 69:239–48. 10.1016/j.freeradbiomed.2014.01.034 24509157

[301] de Jong Y Ingola M Briaire-de Bruijn IH Kruisselbrink AB Venneker S Palubeckaite I. Radiotherapy resistance in chondrosarcoma cells; a possible correlation with alterations in cell cycle related genes. Clin Sarcoma Res (2019) 9(1):9–9. 10.1186/s13569-019-0119-0 31160965 PMC6540537

[302] Buckner J Shaw E Pugh S Gilbert M Barger G Coons S. ATCT-09IDH1 r132h mutations in nrg oncology/rtog 9802: phase iii study of radiation therapy (rt) alone vs rt plus procarbazine, ccnu, and vincristine (pcv) in patients with low grade glioma (lgg). Neuro-Oncology (2015) 17(suppl_5):v3–3. 10.1093/neuonc/nov206.09

[303] Dinardo CD Ravandi F Agresta S Konopleva M Takahashi K Kadia T. Characteristics, clinical outcome, and prognostic significance of IDH mutations in AML. Am J Hematol (2015) 90(8):732–6. 10.1002/ajh.24072 PMC4612499 26016821

[304] Intlekofer AM Shih AH Wang B Nazir A Rustenburg AS Albanese SK. Acquired resistance to IDH inhibition through trans or cis dimer-interface mutations. Nature (2018) 559(7712):125–9. 10.1038/s41586-018-0251-7 PMC6121718 29950729

[305] Konteatis Z Artin E Nicolay B Straley K Padyana AK Jin L. Vorasidenib (AG-881): A First-in-Class, Brain-Penetrant Dual Inhibitor of Mutant IDH1 and 2 for Treatment of Glioma. ACS Medicinal Chem Lett (2020) 11(2):101–7. 10.1021/acsmedchemlett.9b00509 PMC7025383 32071674

[306] Study of AG-120 and AG-881 in Subjects With Low Grade Glioma, Full Text View - ClinicalTrials.gov: NCT03343197.

[307] Phase I Study of BAY1436032 in IDH1-mutant Advanced Solid Tumors, Full Text View - ClinicalTrials.gov: NCT02746081.

[308] Ashton TM Gillies McKenna W Kunz-Schughart LA Higgins GS. Oxidative Phosphorylation as an Emerging Target in Cancer Therapy. Clin Cancer Res (2018) 24(11):2482–90. 10.1158/1078-0432.CCR-17-3070 29420223

[309] Zhao B Luo J Yu T Zhou L Lv H Shang P. Anticancer mechanisms of metformin: A review of the current evidence. Life Sci (2020) 254:117717–7. 10.1016/j.lfs.2020.117717 32339541

[310] Xie J Xia L Xiang W He W Yin H Wang F. Metformin selectively inhibits metastatic colorectal cancer with the KRAS mutation by intracellular accumulation through silencing MATE1. Proc Natl Acad Sci USA (2020) 117(23):13012–22. 10.1073/pnas.1918845117 PMC7293710 32444490

[311] Wang JC Li GY Wang B Han SX Sun X Jiang YN. Metformin inhibits metastatic breast cancer progression and improves chemosensitivity by inducing vessel normalization via PDGF-B downregulation. J Exp Clin Cancer Res (2019) 38(1):235. 10.1186/s13046-019-1211-2 31164151 PMC6549289

[312] Cao H Dong W Qu X Shen H Xu J Zhu L. Metformin Enhances the Therapy Effects of Anti-IGF-1R mAb Figitumumab to NSCLC. Sci Rep (2016) 6(1):1–12. 10.1038/srep31072 27488947 PMC4973270

[313] Wheaton WW Weinberg SE Hamanaka RB Soberanes S Sullivan LB Anso E. Metformin inhibits mitochondrial complex I of cancer cells to reduce tumorigenesis. eLife (2014) 2014(3):e02242. 10.7554/eLife.02242 PMC4017650 24843020

[314] Storozhuk Y Hopmans SN Sanli T Barron C Tsiani E Cutz JC. Metformin inhibits growth and enhances radiation response of non-small cell lung cancer (NSCLC) through ATM and AMPK. Br J Cancer (2013) 108(10):2021–32. 10.1038/bjc.2013.187 PMC3670487 23632475

[315] Wang Y Xu W Yan Z Zhao W Mi J Li J. Metformin induces autophagy and G0/G1 phase cell cycle arrest in myeloma by targeting the AMPK/mTORC1 and mTORC2 pathways. J Exp Clin Cancer Res (2018) 37(1):63. 10.1186/s13046-018-0731-5 29554968 PMC5859411

[316] Zhou X Chen J Yi G Deng M Liu H Liang M. Metformin suppresses hypoxia-induced stabilization of HIF-1’ through reprogramming of oxygen metabolism in hepatocellular carcinoma. Oncotarget (2016) 7(1):873–84. 10.18632/oncotarget.6418 PMC4808039 26621849

[317] Huang R Zhou P-K. HIF-1 signaling: A key orchestrator of cancer radioresistance. Radiat Med Prot (2020) 1(1):7–14. 10.1016/j.radmp.2020.01.006

[318] van Gisbergen MW Offermans K Voets AM Lieuwes NG Biemans R Hoffmann RF. Mitochondrial Dysfunction Inhibits Hypoxia-Induced HIF-1alpha Stabilization and Expression of Its Downstream Targets. Front Oncol (2020) 10:770. 10.3389/fonc.2020.00770 32509579 PMC7248342

[319] Kim EH Kim M-S Cho C-K Jung W-G Jeong YK Jeong J-H. Low and high linear energy transfer radiation sensitization of HCC cells by metformin. J Radiat Res (2014) 55(3):432–42. 10.1093/jrr/rrt131 PMC4014154 24375278

[320] Zannella VE Pra AD Muaddi H McKee TD Stapleton S Sykes J. Reprogramming metabolism with metformin improves tumor oxygenation and radiotherapy response. Clin Cancer Res (2013) 19(24):6741–50. 10.1158/1078-0432.CCR-13-1787 24141625

[321] De Bruycker S Vangestel C Staelens S Wyffels L Detrez J Verschuuren M. Effects of metformin on tumor hypoxia and radiotherapy efficacy: a [18F]HX4 PET imaging study in colorectal cancer xenografts. EJNMMI Res (2019) 9(1):74. 10.1186/s13550-019-0543-4 31375940 PMC6677842

[322] Jeong YK Kim MS Lee JY Kim EH Ha H. Metformin radiosensitizes p53-deficient colorectal cancer cells through induction of G2/M arrest and inhibition of DNA repair proteins. PloS One (2015) 10(11):e0143596. 10.1371/journal.pone.0143596 26599019 PMC4657889

[323] Dowling RJO Lam S Bassi C Mouaaz S Aman A Kiyota T. Metformin Pharmacokinetics in Mouse Tumors: Implications for Human Therapy. Cell Metab (2016) 23(4):567–8. 10.1016/j.cmet.2016.03.006 27076069

[324] Janzer A German NJ Gonzalez-Herrera KN Asara JM Haigis MC Struhl K. Metformin and phenformin deplete tricarboxylic acid cycle and glycolytic intermediates during cell transformation and NTPs in cancer stem cells. Proc Natl Acad Sci U States America (2014) 111(29):10574–9. 10.1073/pnas.1409844111 PMC4115496 25002509

[325] Shitara Y Nakamichi N Norioka M Shima H Kato Y Horie T. Role of Organic Cation/Carnitine Transporter 1 in Uptake of Phenformin and Inhibitory Effect on Complex I Respiration in Mitochondria. Toxicol Sci (2012) 132(1):32–42. 10.1093/toxsci/kfs330 23221006

[326] van Gisbergen MW Voets AM Starmans MH de Coo IF Yadak R Hoffmann RF. How do changes in the mtDNA and mitochondrial dysfunction influence cancer and cancer therapy? Challenges, opportunities and models. Mutat Res Rev Mutat Res (2015) 764:16–30. 10.1016/j.mrrev.2015.01.001 26041263

[327] De Mey S Jiang H Corbet C Wang H Dufait I Law K. Antidiabetic biguanides radiosensitize hypoxic colorectal cancer cells through a decrease in oxygen consumption. Front Pharmacol (2018) 9(OCT):1073. 10.3389/fphar.2018.01073 30337872 PMC6178882

[328] Park JH Kim YH Park EH Lee SJ Kim H Kim A. Effects of metformin and phenformin on apoptosis and epithelial-mesenchymal transition in chemoresistant rectal cancer. Cancer Sci (2019) 110(9):2834–45. 10.1111/cas.14124 PMC6726705 31278880

[329] Miskimins WK Ahn HJ Kim JY Ryu S Jung YS Choi JY. Synergistic anti-cancer effect of phenformin and oxamate. PloS One (2014) 9(1):e85576. 10.1371/journal.pone.0085576 24465604 PMC3897486

[330] Molina JR Sun Y Protopopova M Gera S Bandi M Bristow C. An inhibitor of oxidative phosphorylation exploits cancer vulnerability. Nat Med (2018) 24(7):1036–46. 10.1038/s41591-018-0052-4 29892070

[331] Tsuji A Akao T Masuya T Murai M Miyoshi H. IACS-010759, a potent inhibitor of glycolysis-deficient hypoxic tumor cells, inhibits mitochondrial respiratory complex I through a unique mechanism. J Biol Chem (2020) 295(21):7481–91. 10.1074/jbc.RA120.013366 PMC7247293 32295842

[332] Ellinghaus P Heisler I Unterschemmann K Haerter M Beck H Greschat S. BAY 87-2243, a highly potent and selective inhibitor of hypoxia-induced gene activation has antitumor activities by inhibition of mitochondrial complex I. Cancer Med (2013) 2(5):611–24. 10.1002/cam4.112 PMC3892793 24403227

[333] Helbig L Koi L Brüchner K Gurtner K Hess-Stumpp H Unterschemmann K. BAY 87-2243, a novel inhibitor of hypoxia-induced gene activation, improves local tumor control after fractionated irradiation in a schedule-dependent manner in head and neck human xenografts. Radiat Oncol (2014) 9(1):207. 10.1186/1748-717X-9-207 25234922 PMC4262387

[334] Schöckel L Glasauer A Basit F Bitschar K Truong H Erdmann G. Targeting mitochondrial complex I using BAY 87-2243 reduces melanoma tumor growth. Cancer Metab (2015) 3(1):11–1. 10.1186/s40170-015-0138-0 PMC4615872 26500770

[335] Knecht W Henseling J Löffler M. Kinetics of inhibition of human and rat dihydroorotate dehydrogenase by atovaquone, lawsone derivatives, brequinar sodium and polyporic acid. Chem Biol Interact (2000) 124(1):61–76. 10.1016/s0009-2797(99)00144-1 10658902

[336] Biagini GA Fisher N Berry N Stocks PA Meunier B Williams DP. Acridinediones: Selective and potent inhibitors of the malaria parasite mitochondrial bc1 complex. Mol Pharmacol (2008) 73(5):1347–55. 10.1124/mol.108.045120 18319379

[337] Nilsen A LaCrue AN White KL Forquer IP Cross RM Marfurt J. Quinolone-3-diarylethers: A new class of antimalarial drug. Sci Trans Med (2013) 5(177):177ra37. 10.1126/scitranslmed.3005029 PMC4227885 23515079

[338] Diepart C Karroum O Magat J Feron O Verrax J Calderon PB. Arsenic trioxide treatment decreases the oxygen consumption rate of tumor cells and radiosensitizes solid tumors. Cancer Res (2012) 72(2):482–90. 10.1158/0008-5472.CAN-11-1755 22139377

[339] Russell N Burnett A Hills R Betteridge S Dennis M Jovanovic J. NCRI AML Working Group. Attenuated arsenic trioxide plus ATRA therapy for newly diagnosed and relapsed APL: long-term follow-up of the AML17 trial. Blood (2018) 132(13):1452–4. 10.1182/blood-2018-05-851824 PMC6225356 30097508

[340] Tu J Tu K Xu H Wang L Yuan X Qin X. Improving tumor hypoxia and radiotherapy resistance via in situ nitric oxide release strategy. Eur J Pharmaceutics Biopharm (2020) 150:96–107. 10.1016/j.ejpb.2020.03.003 32151726

[341] Dong LF Low P Dyason JC Wang XF Prochazka L Witting PK. α-Tocopheryl succinate induces apoptosis by targeting ubiquinone-binding sites in mitochondrial respiratory complex II. Oncogene (2008) 27(31):4324–35. 10.1038/onc.2008.69 PMC2668987 18372923

[342] Kulikov AV Vdovin AS Zhivotovsky B Gogvadze V. Targeting mitochondria by α-tocopheryl succinate overcomes hypoxia-mediated tumor cell resistance to treatment. Cell Mol Life Sci (2014) 71(12):2325–33. 10.1007/s00018-013-1489-8 PMC11113468 24142346

[343] Gong Y Agani FH. Oligomycin inhibits HIF-1α expression in hypoxic tumor cells. Am J Physiol - Cell Physiol (2005) 288(5):57–5. 10.1152/ajpcell.00443.2004 15840558

[344] Sun RC Board PG Blackburn AC. Targeting metabolism with arsenic trioxide and dichloroacetate in breast cancer cells. Mol Cancer (2011) 10:142–2. 10.1186/1476-4598-10-142 PMC3240126 22093145

[345] Riobó NA Clementi E Melani M Boveris A Cadenas E Moncada S. Nitric oxide inhibits mitochondrial NADH:ubiquinone reductase activity through peroxynitrite formation. Biochem J (2001) 359(1):139–45. 10.1042/bj3590139 PMC1222129 11563977

[346] Cleeter MWJ Cooper JM Darley-Usmar VM Moncada S Schapira AHV. Reversible inhibition of cytochrome c oxidase, the terminal enzyme of the mitochondrial respiratory chain, by nitric oxide. Implications for neurodegenerative diseases. FEBS Lett (1994) 345(1):50–4. 10.1016/0014-5793(94)00424-2 8194600

[347] Holtsberg FW Ensor CM Steiner MR Bomalaski JS Clark MA. Poly(ethylene glycol) (PEG) conjugated arginine deiminase: Effects of PEG formulations on its pharmacological properties. J Controlled Release (2002) 80(1–3):259–71. 10.1016/S0168-3659(02)00042-1 11943403

[348] Lutgens LCHW Deutz NEP Gueulette J Cleutjens JPM Berger MPF Wouters BG. Citrulline: A physiologic marker enabling quantitation and monitoring of epithelial radiation-induced small bowel damage. Int J Radiat Oncol Biol Phys (2003) 57(4):1067–74. 10.1016/S0360-3016(03)00781-8 14575838

[349] Qiu F Chen YR Liu X Chu CY Shen LJ Xu J. Cancer: Arginine starvation impairs mitochondrial respiratory function in ASS1-deficient breast cancer cells. Sci Signaling (2014) 7(319):ra31. 10.1126/scisignal.2004761 PMC4229039 24692592

[350] Syed N Langer J Janczar K Singh P Lo Nigro C Lattanzio L. Epigenetic status of argininosuccinate synthetase and argininosuccinate lyase modulates autophagy and cell death in glioblastoma. Cell Death Dis (2013) 4(1):e458. 10.1038/cddis.2012.197 23328665 PMC3563985

[351] Allen MD Luong P Hudson C Leyton J Delage B Ghazaly E. Prognostic and therapeutic impact of argininosuccinate synthetase 1 control in bladder cancer as monitored longitudinally by PET imaging. Cancer Res (2014) 74(3):896–907. 10.1158/0008-5472.CAN-13-1702 24285724

[352] Tytell AA Neuman RE. Growth response of stable and primary cell cultures to l-ornithine, l-citrulline, and l-arginine. Exp Cell Res (1960) 20(1):84–91. 10.1016/0014-4827(60)90225-1 13840125

[353] Morris SM. Arginine Metabolism Revisited. J Nutr (2016) 146(12):2579S–86S. 10.3945/jn.115.226621 27934648

[354] Tomlinson BK Thomson JA Bomalaski JS Diaz M Akande T Mahaffey N. Phase i trial of arginine deprivation therapy with ADI-PEG 20 plus docetaxel in patients with advanced malignant solid tumors. Clin Cancer Res (2015) 21(11):2480–6. 10.1158/1078-0432.CCR-14-2610 PMC4452427 25739672

[355] Beddowes E Spicer J Chan PY Khadeir R Garcia Corbacho J Repana D. Phase 1 dose-escalation study of pegylated arginine deiminase, cisplatin, and pemetrexed in patients with argininosuccinate synthetase 1–deficient thoracic cancers. J Clin Oncol (2017) 35(16):1778–85. 10.1200/JCO.2016.71.3230 PMC6141244 28388291

[356] Przystal JM Hajji N Khozoie C Renziehausen A Zeng Q Abaitua F. Efficacy of arginine depletion by ADI-PEG20 in an intracranial model of GBM. Cell Death Dis (2018) 9(12):1–10. 10.1038/s41419-018-1195-4 30546006 PMC6294248

[357] Ph 1-2 Study ADI-PEG 20 Plus FOLFOX in Subjects With Advanced GI Malignancies Focusing on Hepatocellular Carcinoma, Full Text View - ClinicalTrials.gov: NCT02102022.

[358] Ph 2/3 Study in Subjects With MPM to Assess ADI-PEG 20 With Pemetrexed and Cisplatin, Full Text View - ClinicalTrials.gov: NCT02709512.

[359] Wright C Iyer AKV Kaushik V Azad N. Anti-Tumorigenic Potential of a Novel Orlistat-AICAR Combination in Prostate Cancer Cells. J Cell Biochem (2017) 118(11):3834–45. 10.1002/jcb.26033 PMC5603373 28387458

[360] Chuang HY Lee YP Lin WC Lin YH Hwang JJ. Fatty Acid Inhibition Sensitizes Androgen-Dependent and -Independent Prostate Cancer to Radiotherapy via FASN/NF-kappaB Pathway. Sci Rep (2019) 9(1):13284. 10.1038/s41598-019-49486-2 31527721 PMC6746859

[361] Agostini M Almeida LY Bastos DC Ortega RM Moreira FS Seguin F. The fatty acid synthase inhibitor orlistat reduces the growth and metastasis of orthotopic tongue oral squamous cell carcinomas. Mol Cancer Ther (2014) 13(3):585–95. 10.1158/1535-7163.MCT-12-1136 24362464

[362] Grabacka M Placha W Plonka PM Pajak S Urbanska K Laidler P. Inhibition of melanoma metastases by fenofibrate. Arch Dermatol Res (2004) 296(2):54–8. 10.1007/s00403-004-0479-y 15278363

[363] Liu J Ge YY Zhu HC Yang X Cai J Zhang C. Fenofibrate increases radiosensitivity in head and neck squamous cell carcinoma via inducing G2/M arrest and apoptosis. Asian Pacific J Cancer Prev (2014) 15(16):6649–55. 10.7314/APJCP.2014.15.16.6649 25169503

[364] Kim W Lee S Seo D Kim D Kim K Kim E. Cellular Stress Responses in Radiotherapy. Cells (2019) 8(9):1105. 10.3390/cells8091105 PMC6769573 31540530

[365] van Gisbergen MW Voets AM Biemans R Hoffmann RF Drittij-Reijnders MJ Haenen G. Distinct radiation responses after in vitro mtDNA depletion are potentially related to oxidative stress. PloS One (2017) 12(8):e0182508. 10.1371/journal.pone.0182508 28771582 PMC5542624

[366] Glasauer A Chandel NS. Targeting antioxidants for cancer therapy. Biochem Pharmacol (2014) 92(1):90–101. 10.1016/j.bcp.2014.07.017 25078786

[367] Zhang Y Martin SG. Redox proteins and radiotherapy. Clin Oncol (R Coll Radiol) (2014) 26(5):289–300. 10.1016/j.clon.2014.02.003 24581945

[368] Lei G Zhang Y Koppula P Liu X Zhang J Lin SH. The role of ferroptosis in ionizing radiation-induced cell death and tumor suppression. Cell Res (2020) 30(2):146–62. 10.1038/s41422-019-0263-3 PMC7015061 31949285

[369] Lang X Green MD Wang W Yu J Choi JE Jiang L. Radiotherapy and immunotherapy promote tumoral lipid oxidation and ferroptosis via synergistic repression of SLC7A11. Cancer Discovery (2019) 9(12):1673–85. 10.1158/2159-8290.CD-19-0338 PMC6891128 31554642

[370] Cobler L Zhang H Suri P Park C Timmerman LA. xCT inhibition sensitizes tumors to ?-radiation via glutathione reduction. Oncotarget (2018) 9(64):32280–97. 10.18632/oncotarget.25794 PMC6122354 30190786

[371] Ye LF Chaudhary KR Zandkarimi F Harken AD Kinslow CJ Upadhyayula PS. Radiation-Induced Lipid Peroxidation Triggers Ferroptosis and Synergizes with Ferroptosis Inducers. ACS Chem Biol (2020) 15(2):469–84. 10.1021/acschembio.9b00939 PMC7180072 31899616

[372] Sappington DR Siegel ER Hiatt G Desai A Penney RB Jamshidi-Parsian A. Glutamine drives glutathione synthesis and contributes to radiation sensitivity of A549 and H460 lung cancer cell lines. Biochim Biophys Acta - Gen Subj (2016) 1860(4):836–43. 10.1016/j.bbagen.2016.01.021 PMC4768472 26825773

[373] Huang Q Stalnecker C Zhang C McDermott LA Iyer P O’Neill J. Characterization of the interactions of potent allosteric inhibitors with glutaminase C, a key enzyme in cancer cell glutamine metabolism. J Biol Chem (2018) 293(10):3535–45. 10.1074/jbc.M117.810101 PMC5846160 29317493

[374] Shukla K Ferraris DV Thomas AG Stathis M Duvall B Delahanty G. Design, synthesis, and pharmacological evaluation of bis-2-(5- phenylacetamido-1,2,4-thiadiazol-2-yl)ethyl sulfide 3 (BPTES) analogs as glutaminase inhibitors. J Medicinal Chem (2012) 55(23):10551–63. 10.1021/jm301191p PMC3539823 23151085

[375] Meric-Bernstam F Lee RJ Carthon BC Iliopoulos O Mier JW Patel MR. CB-839, a glutaminase inhibitor, in combination with cabozantinib in patients with clear cell and papillary metastatic renal cell cancer (mRCC): Results of a phase I study. J Clin Oncol (2019) 37(7_suppl):549–9. 10.1200/JCO.2019.37.7_suppl.549

[376] Study of the Glutaminase Inhibitor CB-839 in Solid Tumors, Full Text View - ClinicalTrials.gov.

[377] Vartak S Robbins MEC Spector AA. Polyunsaturated fatty acids increase the sensitivity of 36B10 rat astrocytoma cells to radiation-induced cell kill. Lipids (1997) 32(3):283–92. 10.1007/s11745-997-0035-y 9076665

[378] Colas S Paon L Denis F Prat M Louisot P Hoinard C. Enhanced radiosensitivity of rat autochthonous mammary tumors by dietary docosahexaenoic acid. Int J Cancer (2004) 109(3):449–54. 10.1002/ijc.11725 14961586

[379] Wen B Deutsch E Opolon P Auperin A Frascogna V Connault E. n-3 Polyunsaturated fatty acids decrease mucosal/epidermal reactions and enhance antitumour effect of ionising radiation with inhibition of tumour angiogenesis. Br J Cancer (2003) 89(6):1102–7. 10.1038/sj.bjc.6601136 PMC2376938 12966433

[380] Marín A Martín M Liñán O Alvarenga F López M Fernández L. Bystander effects and radiotherapy. Rep Pract Oncol Radiother (2015) 20(1):12–21. 10.1016/j.rpor.2014.08.004 25535579 PMC4268598

[381] Fuzissaki MDA Paiva CE M.A.D. Oliveira Lajolo Canto PP Paiva Maia YCD. The Impact of Radiodermatitis on Breast Cancer Patients’ Quality of Life During Radiotherapy: A Prospective Cohort Study. J Pain Symptom Manage (2019) 58(1):92–99.e1. 10.1016/j.jpainsymman.2019.03.017 30974233

[382] Giatromanolaki A Sivridis E Maltezos E Koukourakis MI. Down-regulation of intestinal-type alkaline phosphatase in the tumor vasculature and stroma provides a strong basis for explaining amifostine selectivity. Semin Oncol(2002) 29(6 Suppl 19):14–21. 10.1053/sonc.2002.37356 12577238

[383] Grdina DJ Shigematsu N Dale P Newton GL Aguilera JA Fahey RC. Thiol and disulfide metabolites of the radiation protector and potential chemopreventive agent WR-2721 are linked to both its anti-cytotoxic and anti-mutagenic mechanisms of action. Carcinogenesis (1995) 16(4):767–74. 10.1093/carcin/16.4.767 7728953

[384] Dziegielewski J Baulch JE Goetz W Coleman MC Spitz DR Murley JS. WR-1065, the active metabolite of amifostine, mitigates radiation-induced delayed genomic instability. Free Radical Biol Med (2008) 45(12):1674–81. 10.1016/j.freeradbiomed.2008.09.004 PMC2629584 18845240

[385] Koukourakis MI Giatromanolaki A Zois CE Kalamida D Pouliliou S Karagounis IV. Normal tissue radioprotection by amifostine via Warburg-Type effects. Sci Rep (2016) 6:30986. 10.1038/srep30986 27507219 PMC4978965

[386] Savoye C Swenberg C Hugot S Sy D Sabattier R Charlier M. Thiol WR-1065 and disulphide WR-33278, two metabolites of the drug Ethyol (WR-2721), protect DNA against fast neutron-induced strand breakage. Int J Radiat Biol (1997) 71(2):193–202. 10.1080/095530097144319 9120355

[387] Cakmak Arslan G Severcan F. The effects of radioprotectant and potential antioxidant agent amifostine on the structure and dynamics of DPPC and DPPG liposomes. Biochim Biophys Acta - Biomembr (2019) 1861(6):1240–51. 10.1016/j.bbamem.2019.04.009 31028720

[388] Kouloulias VE Kouvaris JR Pissakas G Kokakis JD Antypas C Mallas E. A Phase II Randomized Study of Topical Intrarectal Administration of Amifostine for the Prevention of Acute Radiation-Induced Rectal Toxicity. Strahlentherapie und Onkologie (2004) 180(9):557–62. 10.1007/s00066-004-1226-1 15378186

[389] Kouloulias VE Kouvaris JR Pissakas G Mallas E Antypas C Kokakis JD. Phase II multicenter randomized study of amifostine for prevention of acute radiation rectal toxicity: Topical intrarectal versus subcutaneous application. Int J Radiat Oncol Biol Phys (2005) 62(2):486–93. 10.1016/j.ijrobp.2004.10.043 15890591

[390] Soref CM Hacker TA Fahl WE. A new orally active, aminothiol radioprotector-free of nausea and hypotension side effects at its highest radioprotective doses. Int J Radiat Oncol Biol Phys (2012) 82(5):e701–7. 10.1016/j.ijrobp.2011.11.038 22330992

[391] Everett WH Curiel DT. Gene therapy for radioprotection. Cancer Gene Ther (2015) 22(4):172–80. 10.1038/cgt.2015.8 PMC4608443 25721205

[392] Epperly MW Dixon T Wang H Schlesselman J Franicola D Greenberger JS. Modulation of radiation-induced life shortening by systemic intravenous MnSOD-plasmid liposome gene therapy. Radiat Res (2008) 170(4):437–43. 10.1667/RR1286.1 PMC2593730 19024650

[393] Epperly MW Defilippi S Sikora C Gretton J Kalend A Greenberger JS. Intratracheal injection of manganese superoxide dismutase (MnSOD) plasmid/liposomes protects normal lung but not orthotopic tumors from irradiation. Gene Ther (2000) 7(12):1011–8. 10.1038/sj.gt.3301207 10871749

[394] Epperly MW Sacher JR Krainz T Zhang X Wipf P Liang M. Effectiveness of analogs of the GS-Nitroxide, JP4-039, as total body irradiation mitigators. Vivo (2017) 31(1):39–44. 10.21873/invivo.11022 PMC5267945 28064218

[395] Gokhale A Rwigema J-C Epperly MW Glowacki J Wang H Wipf P. Small molecule GS-nitroxide ameliorates ionizing irradiation-induced delay in bone wound healing in a novel murine model. Vivo (Athens Greece) (2010) 24(4):377–85. PMC2916688 20668303

[396] Epperly MW Goff JP Li S Gao X Wipf P Dixon T. Intraesophageal administration of GS-nitroxide (JP4-039) protects against ionizing irradiation-induced esophagitis. Vivo (2010) 24(6):811–9. PMC3521523 21164038

[397] Wei L Leibowitz BJ Epperly M Bi C Li A Steinman J. The GS-nitroxide JP4-039 improves intestinal barrier and stem cell recovery in irradiated mice. Sci Rep (2018) 8(1):1–12. 10.1038/s41598-018-20370-9 29391546 PMC5794877

[398] Kunwar A Bag PP Chattopadhyay S Jain VK Priyadarsini KI. Anti-apoptotic, anti-inflammatory, and immunomodulatory activities of 3,3′-diselenodipropionic acid in mice exposed to whole body γ-radiation. Arch Toxicol (2011) 85(11):1395–405. 10.1007/s00204-011-0687-0 21380500

[399] Yusup G Akutsu Y Mutallip M Qin W Hu X Komatsu-Akimoto A. A COX-2 inhibitor enhances the antitumor effects of chemotherapy and radiotherapy for esophageal squamous cell carcinoma. Int J Oncol (2014) 44(4):1146–52. 10.3892/ijo.2014.2300 24535229

[400] Elshawi OE Nabeel AI. Modulatory effect of a new benzopyran derivative via COX-2 blocking and down regulation of NF-κB against γ-radiation induced- intestinal inflammation. J Photochem Photobiol B: Biol (2019) 192:90–6. 10.1016/j.jphotobiol.2019.01.006 30710830

[401] Liang L Hu D Liu W Williams JP Okunieff P Ding I. Celecoxib Reduces Skin Damage After Radiation. Am J Clin Oncol (2003) 26(Supplement 2):S114–21. 10.1097/01.COC.0000074149.95710.40 12902868

[402] Raju U Ariga H Dittmann K Nakata E Ang KK Milas L. Inhibition of DNA repair as a mechanism of enhanced radioresponse of head and neck carcinoma cells by a selective cyclooxygenase-2 inhibitor, celecoxib. Int J Radiat Oncol Biol Phys (2005) 63(2):520–8. 10.1016/j.ijrobp.2005.06.007 16168844

[403] Zhang P He D Song E Jiang M Song Y. Celecoxib enhances the sensitivity of nonsmall- cell lung cancer cells to radiationinduced apoptosis through downregulation of the Akt/mTOR signaling pathway and COX-2 expression. PloS One (2019) 14(10):e0223760. 10.1371/journal.pone.0223760 31613929 PMC6793859

[404] Bi N Liang J Zhou Z Chen D Fu Z Yang X. Effect of Concurrent Chemoradiation With Celecoxib vs Concurrent Chemoradiation Alone on Survival Among Patients With Non-Small Cell Lung Cancer With and Without Cyclooxygenase 2 Genetic Variants: A Phase 2 Randomized Clinical Trial. JAMA Netw Open (2019) 2(12):e1918070–e1918070. 10.1001/jamanetworkopen.2019.18070 31851351 PMC6991217

[405] Cho YJ Yi CO Jeon BT Jeong YYE Kang GM Lee JE. Curcumin attenuates radiation-induced inflammation and fibrosis in rat lungs. Korean J Physiol Pharmacol (2013) 17(4):267–74. 10.4196/kjpp.2013.17.4.267 PMC3741482 23946685

[406] Amini P Saffar H Nourani MR Motevaseli E Najafi M Taheri RA. Curcumin mitigates radiation-induced lung pneumonitis and fibrosis in rats. Int J Mol Cell Med (2018) 7(4):212–9. 10.22088/IJMCM.BUMS.7.4.212 PMC6709933 31516880

[407] Abadi SHMH Shirazi A Alizadeh AM Changizi V Najafi M Khalighfard S. The Effect of Melatonin on Superoxide Dismutase and Glutathione Peroxidase Activity, and Malondialdehyde Levels in the Targeted and the Non-targeted Lung and Heart Tissues after Irradiation in Xenograft Mice Colon Cancer. Curr Mol Pharmacol (2018) 11(4):326–35. 10.2174/1874467211666180830150154 30173656

[408] Najafi M Shirazi A Motevaseli E Geraily G Amini P Tooli LF. Melatonin Modulates Regulation of NOX2 and NOX4 Following Irradiation in the Lung. Curr Clin Pharmacol (2019) 14(3):224–31. 10.2174/1574884714666190502151733 31057124

[409] Reiter RJ Rosales-Corral SA Zhou X Tan D-X. Role of SIRT3/SOD2 signaling in mediating the antioxidant actions of melatonin in mitochondria. Curr Trends Endocrinol (2017) 9:45–49.

[410] Rezapoor S Shirazi A Abbasi S Bazzaz J Izadi P Rezaeejam H. Modulation of radiation-induced base excision repair pathway gene expression by melatonin. J Med Phys (2017) 42(4):245–50. 10.4103/jmp.JMP_9_17 PMC5744453 29296039

[411] Valizadeh M Shirazi A Izadi P Bazzaz JT Rezaeejam H. Expression Levels of Two DNA Repair-related Genes under 8 Gy Ionizing Radiation and 100 Mg/Kg Melatonin Delivery In Rat Peripheral Blood. J Biomed Phys Eng (2017) 7(1):27–7. PMC5401131 28451577

[412] Fernandez-Gil B Abdel Moneim AE Ortiz F Shen YQ Soto-Mercado V Mendivil-Perez M. Melatonin protects rats from radiotherapyinduced small intestine toxicity. PloS One (2017) 12(4):e0174474. 10.1371/journal.pone.0174474 28403142 PMC5389624

[413] Najafi M Cheki M Hassanzadeh G Amini P Shabeeb D Eleojo Musa A. Protection from radiation-induced damage in rat’s ileum and colon by combined regimens of melatonin and metformin: A histopathological study. Anti Inflammatory Anti Allergy Agents Medicinal Chem (2019) 18:180–189. 10.2174/1871523018666190718161928 PMC7475942 31438832

[414] Martın M Macıas M León J Escames G Khaldy H Acuña-Castroviejo DO. Melatonin increases the activity of the oxidative phosphorylation enzymes and the production of ATP in rat brain and liver mitochondria. Int J Biochem Cell Biol (2002) 34(4):348–57. 10.1016/S1357-2725(01)00138-8 11854034

[415] Alonso-González C González A Martínez-Campa C Gómez-Arozamena J Cos S. Melatonin sensitizes human breast cancer cells to ionizing radiation by downregulating proteins involved in double-strand DNA break repair. J Pineal Res (2015) 58(2):189–97. 10.1111/jpi.12205 25623566

[416] Reiter RJ Sharma R Ma Q Rorsales-Corral S de Almeida Chuffa LG. Melatonin inhibits Warburg-dependent cancer by redirecting glucose oxidation to the mitochondria: a mechanistic hypothesis. Cell Mol Life Sci (2020) 77(13):2527–42. 10.1007/s00018-019-03438-1 PMC11104865 31970423

[417] Kouhi Habibi N Shabestani Monfared A Ebrahimnejad Gorji K Karimi M Moghadamnia AA Tourani M. The protective effects of melatonin on blood cell counts of rectal cancer patients following radio-chemotherapy: a randomized controlled trial. Clin Trans Oncol (2019) 21(6):745–52. 10.1007/s12094-018-1977-2 30421178

[418] Lee YY Kao CL Chiou SH Tsai PH Tsai TH Wu WF. Caffeic acid phenethyl ester preferentially enhanced radiosensitizing and increased oxidative stress in medulloblastoma cell line. Child’s Nervous System (2008) 24(9):987–94. 10.1007/s00381-008-0636-2 18470517

[419] Liang Y Feng G Wu L Zhong S Gao X Tong Y. Caffeic acid phenethyl ester suppressed growth and metastasis of nasopharyngeal carcinoma cells by inactivating the NF-κB pathway. Drug Design Dev Ther (2019) 13:1335–45. 10.2147/DDDT.S199182 PMC6499142 31118570

[420] Khoram NM Bigdeli B Nikoofar A Goliaei B. Caffeic acid phenethyl ester increases radiosensitivity of estrogen receptor- positive and -negative breast cancer cells by prolonging radiation-induced DNA Damage. J Breast Cancer (2016) 19(1):18–25. 10.4048/jbc.2016.19.1.18 27066092 PMC4822103

[421] Singh PK Krishnan S. Vitamin E Analogs as Radiation Response Modifiers. Evid Based Complement Alternat Med (2015) 2015:741301. 10.1155/2015/741301 26366184 PMC4558447

[422] Karim R Somani S Al Robaian M Mullin M Amor R McConnell G. Tumor regression after intravenous administration of targeted vesicles entrapping the vitamin E α-tocotrienol. J Controlled Release (2017) 246:79–87. 10.1016/j.jconrel.2016.12.014 27993600

[423] Cremona CA Behrens A. ATM signalling and cancer. Oncogene (2014) 33(26):3351–60. 10.1038/onc.2013.275 23851492

[424] Yin H Glass J. The phenotypic radiation resistance of CD44 +/CD24 -or low breast cancer cells is mediated through the enhanced activation of ATM signaling. PloS One (2011) 6(9):e24080. 10.1371/journal.pone.0024080 21935375 PMC3174160

[425] Scholz A Heinze S Detjen KM Peters M Welzel M Hauff P. Activated signal transducer and activator of transcription 3 (STAT3) supports the malignant phenotype of human pancreatic cancer. Gastroenterology (2003) 125(3):891–905. 10.1016/S0016-5085(03)01064-3 12949733

[426] Quoc Trung L Espinoza JL Takami A Nakao S. Resveratrol Induces Cell Cycle Arrest and Apoptosis in Malignant NK Cells via JAK2/STAT3 Pathway Inhibition. PloS One (2013) 8(1):e55183. 10.1371/journal.pone.0055183 23372833 PMC3555980

[427] Pawlik TM Keyomarsi K. Role of cell cycle in mediating sensitivity to radiotherapy. Int J Radiat Oncol Biol Phys (2004) 59(4):928–42. 10.1016/j.ijrobp.2004.03.005 15234026

[428] Duan L Maki CG. The IGF-1R/AKT pathway determines cell fate in response to p53. Trans Cancer Res (2016) 5(6):664–75. 10.21037/tcr.2016.09.16 PMC5617345 28966916

[429] Tan Y Wei X Zhang W Wang X Wang K Du B. Resveratrol enhances the radiosensitivity of nasopharyngeal carcinoma cells by downregulating E2F1. Oncol Rep (2017) 37(3):1833–41. 10.3892/or.2017.5413 28184930

[430] Lee SR Jin H Kim WT Kim WJ Kim SZ Leem SH. Tristetraprolin activation by resveratrol inhibits the proliferation and metastasis of colorectal cancer cells. Int J Oncol (2018) 53(3):1269–78. 10.3892/ijo.2018.4453 29956753

[431] Wong JV Dong P Nevins JR Mathey-Prevot B You L. Network calisthenics: control of E2F dynamics in cell cycle entry. Cell Cycle (2011) 10(18):3086–94. 10.4161/cc.10.18.17350 PMC3218619 21900750

[432] da Costa Araldi IC Bordin FPR Cadoná FC Barbisan F Azzolin VF Teixeira CF. The in vitro radiosensitizer potential of resveratrol on MCF-7 breast cancer cells. Chemico Biological Interact (2018) 282:85–92. 10.1016/j.cbi.2018.01.013 29336987

[433] Wu Z Liu B Cailing E Liu J Zhang Q Liu J. Resveratrol inhibits the proliferation of human melanoma cells by inducing G1/S cell cycle arrest and apoptosis. Mol Med Rep (2015) 11(1):400–4. 10.3892/mmr.2014.2716 25333673

[434] Yu XD Yang JL Zhang WL Liu DX. Resveratrol inhibits oral squamous cell carcinoma through induction of apoptosis and G2/M phase cell cycle arrest. Tumor Biol (2016) 37(3):2871–7. 10.1007/s13277-015-3793-4 26409447

[435] Carsten RE Bachand AM Bailey SM Ullrich RL. Resveratrol Reduces Radiation-Induced Chromosome Aberration Frequencies in Mouse Bone Marrow Cells. Radiat Res (2008) 169(6):633–8. 10.1667/RR1190.1 PMC2692544 18494544

[436] Zhang H Yan H Zhou X Wang H Yang Y Zhang J. The protective effects of Resveratrol against radiation-induced intestinal injury. BMC Complement Altern Med (2017) 17(1):410. 10.1186/s12906-017-1915-9 28814292 PMC5559783

[437] Thekkekkara D Basavan D Chandna S Nanjan MJ. A combination of resveratrol and 3,3′-diindolylmethane, a potent radioprotector. Int J Radiat Biol (2018) 94(6):558–68. 10.1080/09553002.2018.1467063 29671693

[438] Lee JH Guo Z Myler LR Zheng S Paull TT. Direct activation of ATM by resveratrol under oxidizing conditions. PloS One (2014) 9(6):e97969. 10.1371/journal.pone.0097969 24933654 PMC4059639

[439] Chimento A De Amicis F Sirianni R Sinicropi MS Puoci F Casaburi I. Progress to Improve Oral Bioavailability and Beneficial Effects of Resveratrol. Int J Mol Sci (2019) 10(6):1381. 10.3390/ijms20061381 PMC6471659 30893846

[440] Shi G Rao L Yu H Xiang H Yang H Ji R. Stabilization and encapsulation of photosensitive resveratrol within yeast cell. Int J Pharmaceutics (2008) 349(1-2):83–93. 10.1016/j.ijpharm.2007.07.044 17869035

[441] Ho VW Leung K Hsu A Luk B Lai J Shen SY. A low carbohydrate, high protein diet slows tumor growth and prevents cancer initiation. Cancer Res (2011) 71(13):4484–93. 10.1158/0008-5472.CAN-10-3973 21673053

[442] Abdelwahab MG Fenton KE Preul MC Rho JM Lynch A Stafford P. The ketogenic diet is an effective adjuvant to radiation therapy for the treatment of malignant glioma. PloS One (2012) 7(5):e36197. 10.1371/journal.pone.0036197 22563484 PMC3341352

[443] van der Louw EJTM Reddingius RE Olieman JF Neuteboom RF Catsman-Berrevoets CE. Ketogenic diet treatment in recurrent diffuse intrinsic pontine glioma in children: A safety and feasibility study. Pediatr Blood Cancer (2019) 66(3):e27561–1. 10.1002/pbc.27561 30484948

[444] Lee C Raffaghello L Brandhorst S Safdie FM Bianchi G Martin-Montalvo A. Fasting cycles retard growth of tumors and sensitize a range of cancer cell types to chemotherapy. Sci Trans Med (2012) 4(124):124ra27. 10.1126/scitranslmed.3003293 PMC3608686 22323820

[445] Allen BG Bhatia SK Buatti JM Brandt KE Lindholm KE Button AM. Ketogenic diets enhance oxidative stress and radio-chemo-therapy responses in lung cancer xenografts. Clin Cancer Res (2013) 19(14):3905–13. 10.1158/1078-0432.CCR-12-0287 PMC3954599 23743570

[446] Sperry J Belle JEL Condro MC Guo L Braas D Vanderveer-Harris N. Metabolism of fatty acids and ketone bodies for glioblastoma growth: Implications for Ketogenic Diet Therapy. bioRxiv (2019) p:659474–4. 10.1101/659474

[447] Shi Y Felley-Bosco E Marti TM Orlowski K Pruschy M Stahel RA. Starvation-induced activation of ATM/Chk2/p53 signaling sensitizes cancer cells to cisplatin. BMC Cancer (2012) 12:571–1. 10.1186/1471-2407-12-571 PMC3527202 23211021

[448] Kwon M Kim RB Roh JL Lee SW Kim SB Choi SH. Prevalence and clinical significance of cancer cachexia based on time from treatment in advanced-stage head and neck squamous cell carcinoma. Head Neck (2017) 39(4):716–23. 10.1002/hed.24672 28000343

[449] Zahra A Fath MA Opat E Mapuskar KA Bhatia SK Ma DC. Consuming a Ketogenic Diet while Receiving Radiation and Chemotherapy for Locally Advanced Lung Cancer and Pancreatic Cancer: The University of Iowa Experience of Two Phase 1 Clinical Trials. Radiat Res (2017) 187(6):743–54. 10.1667/RR14668.1 PMC5510645 28437190

[450] Klement RJ Champ CE Kammerer U Koebrunner PS Krage K Schafer G. Impact of a ketogenic diet intervention during radiotherapy on body composition: III-final results of the KETOCOMP study for breast cancer patients. Breast Cancer Res (2020) 22(1):94. 10.1186/s13058-020-01331-5 32819413 PMC7441712

[451] Cuyàs E Fernández-Arroyo S Buxó M Pernas S Dorca J Álvarez I. Metformin induces a fasting- and antifolate-mimicking modification of systemic host metabolism in breast cancer patients. Aging (2019) 11(9):2874–88. 10.18632/aging.101960 PMC6535060 31076561

